# Pyridoxal and Salicylaldehyde Derivatives: Synthesis, Characterization, and Antifungal Potential Against Opportunistic Yeast Pathogens

**DOI:** 10.3390/molecules30051165

**Published:** 2025-03-05

**Authors:** Jairo Camacho, Carlos A. Bejarano, John E. Diaz, Yerly Vargas-Casanova, Silvia Katherine Carvajal, Valentina Diaz Santoyo, Claudia M. Parra-Giraldo, Alix E. Loaiza

**Affiliations:** 1 Departamento de Química, Facultad de Ciencias, Pontificia Universidad Javeriana, Cra. 7 No. 40-62, Bogotá 110231, Colombia; jairo_camachoo@javeriana.edu.co (J.C.); claudia.parra@universidadeuropea.es (C.M.P.-G.); 2 Departamento de Química, Facultad de Ciencias, Universidad Antonio Nariño, Cra. 3 Este No. 47 A-15, Bogotá 110231, Colombia; cbejaranoc@uan.edu.co (C.A.B.); jdiaz77@uan.edu.co (J.E.D.); 3 Departamento de Microbiología, Facultad de Ciencias, Pontificia Universidad Javeriana, Cra. 7 No. 40-62, Bogotá 110231, Colombia; yerly.vargas@itp.edu.co (Y.V.-C.); sicarvajal@udca.edu.co (S.K.C.); valentina_diaz@javeriana.edu.co (V.D.S.); 4 Facultad de Ingeniería y Ciencias Básicas, Instituto Tecnológico del Putumayo, Mocoa 860001, Colombia; 5 Departamento de Biomedicina, Facultad de Ciencias de la Salud y Biociencias, Universidad Europea de Madrid, 28670 Madrid, Spain

**Keywords:** pyridoxal, salicylaldehyde, radical cyclizations, click chemistry, antifungal activity, *C. albicans*, *C. auris*, *C. neoformans*

## Abstract

This study reports the synthesis, characterization, and antifungal evaluation of a series of pyridoxal and salicylaldehyde derivatives, using synthetic methodologies such as radical cyclizations and click chemistry. Compounds **6a** and **6b**, featuring a fused dihydrobenzoxepine-pyridine scaffold, demonstrated effective fungicidal activity with MIC values of 19 µg/mL against *Cryptococcus neoformans* 2807. Similarly, compound **6b** exhibited notable activity with a MIC of 75 µg/mL against *Candida auris* PUJ-HUSI 537. Both compounds outperformed fluconazole (FLC) in these strains. In silico ADMET profiling revealed favorable pharmacokinetic properties, including blood–brain barrier penetration and drug-likeness parameters consistent with Lipinski’s rule of five. Cytotoxicity assays on human fibroblasts confirmed the low toxicity of compound **6a** at the tested concentrations. These results highlight the potential of the fused dihydrobenzoxepine-pyridine scaffold as a promising antifungal candidate for further investigations.

## 1. Introduction

Invasive fungal diseases (IFDs) have emerged as a significant public health challenge in recent decades, responsible for over 1.7 million deaths worldwide annually [1]. *Candida* spp. and *Cryptococcus* spp. are major pathogens causing these diseases: *C. albicans* being the primary etiological agent, a commensal colonizer of the skin and mucous membranes, which can cause invasive candidiasis (IC) if the surrounding environment favors it [2,3]. *C. auris* is another troubling pathogen, noted for its rapid spread in healthcare settings, high mortality rates, and resistance to multiple drugs, particularly in intensive care units (ICUs) [4]. *C. neoformans*, an opportunistic fungus, can lead to pulmonary cryptococcosis with potential dissemination to the central nervous system (CNS), causing meningitis, especially in immunocompromised patients [5]. These infections highlight the urgent need for new therapeutic approaches due to the limited efficacy, toxicity, and challenges in penetrating the blood–brain barrier associated with current antifungal therapies.

IC poses significant clinical challenges, predominantly caused by *C. albicans* and increasingly by *C. auris*, both of which exhibit varying degrees of resistance to standard antifungal therapies [6]. Current treatment options typically include echinocandins such as caspofungin, micafungin, and anidulafungin, which inhibit β-(1,3)-D-glucan synthesis in the fungal cell wall and are considered first-line agents [7]. However, the emergence of resistance to echinocandins among *Candida* spp., including *C. albicans* and *C. auris* strains, necessitates alternative therapeutic strategies. Triazoles like FLC and voriconazole, along with amphotericin B formulations, are commonly used alternatives, though their efficacy can be compromised by resistance patterns [8,9,10]. Combination therapies, which use different classes of antifungals or include novel agents, are increasingly explored to improve treatment outcomes against multidrug-resistant IC. Novel antifungal agents with improved pharmacokinetic profiles and activity against resistant strains are particularly promising in the ongoing effort to manage and treat IC effectively.

On the other hand, in cryptococcosis, a combination of flucytosine and FLC or amphotericin B is frequently employed for the treatment of cryptococcal meningitis [11]. These antifungal agents can penetrate brain tissue effectively; however, flucytosine is associated with significant adverse effects, including hepatotoxicity and myelotoxicity. Additionally, *C. neoformans* can develop increased tolerance to FLC, promoting the selection of heteroresistant clones. Amphotericin B, another pivotal antifungal in cryptococcosis treatment, faces limitations in CNS absorption due to its large molecular size [12]. Efforts to enhance amphotericin B formulations for improved CNS penetration are ongoing; nonetheless, challenges such as high cost and supply chain issues restrict widespread availability. Consequently, therapeutic options for *C. neoformans* infections remain limited, highlighting the critical need to explore and develop new therapeutic strategies to fight against IFDs [13,14,15,16,17,18,19].

Benzoxepines and triazoles are of significant interest due to their remarkable biological activities, including antifungal, anthelmintic, antibacterial, anticancer, and effects on the central nervous system [20,21,22,23,24,25,26,27]. In contrast, dihydrobenzoxepines fused with a pyridine motif remain underexplored, to the best of our knowledge, and offer a promising scaffold for the development of novel antifungal agents. In this context, and with the aim of contributing to the development of new treatments for IFDs, our research group designed synthetic routes to obtain compounds with these structural features, using pyridoxal and salicylaldehyde as starting materials. The synthesized compounds were evaluated against strains of *C. albicans*, *C. auris*, and *C. neoformans*.

## 2. Results and Discussion

### 2.1. Chemistry

#### 2.1.1. Synthesis of Pyridoxal Derivatives **2**–**5**

**Scheme 1:**
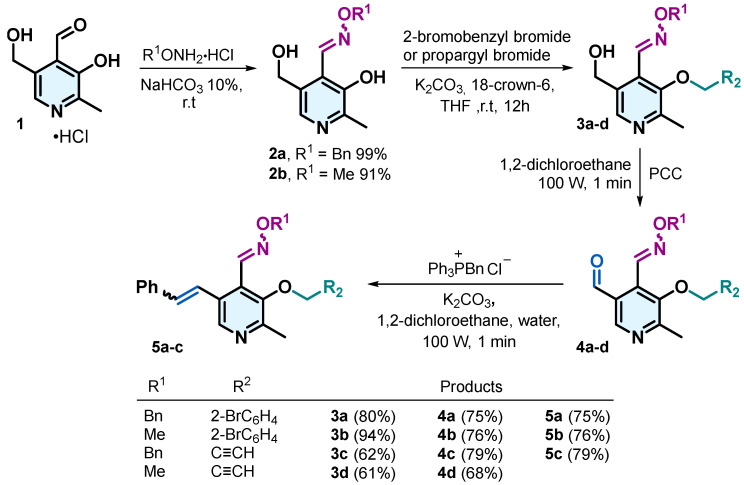
Synthesis of pyridoxal derivatives **2**–**5**.

Oxime ethers **2a** and **2b** were synthesized by condensing the aldehyde group of pyridoxal **1** with *O*-methylhydroxylamine or *O*-benzylhydroxylamine hydrochloride and 10% aqueous NaHCO_3_, achieving yields exceeding 90%. The hydroxyl group attached to the pyridine ring in compounds **2a** and **2b** was alkylated with 2-bromobenzyl bromide or propargyl bromide using K_2_CO_3_ and 18-crown-6 ether in dry THF, yielding compounds **3a**–**d** with yields above 60%. Subsequently, the hydroxymethyl group in compounds **3** was oxidized to an aldehyde using pyridinium chlorochromate (PCC) in 1,2-dichloroethane under microwave irradiation, ref. [28] affording compounds **4a**–**d** with yields ranging from 68% to 75%. Compounds **4a**–**c** were then subjected to a Wittig reaction with benzyltriphenylphosphonium chloride in the presence of K_2_CO_3_, 1,2-dichloroethane and water under microwave irradiation [28]. This reaction yielded compounds **5a**–**c** as an *E/Z* mixture of isomers, with yields ranging from 58% to 89% (Scheme 1).

#### 2.1.2. Synthesis of Dihydrobenzoxepines **6a** and **6b**

**Scheme 2:**
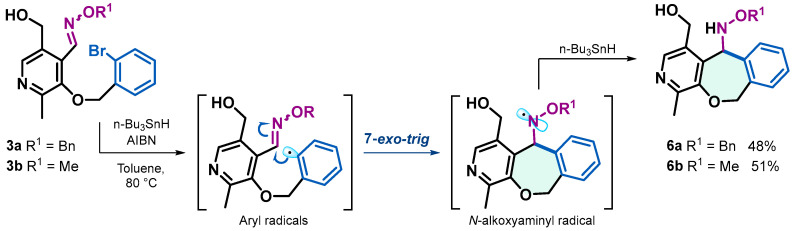
Synthesis of dihydrobenzoxepines **6a** and **6b** through 7-*exo-trig* radical cyclization reactions.

The synthesis of compounds with a dihydrobenzoxepine core fused to pyridine **6a** and **6b** was performed via radical cyclization reactions starting from **3a** and **3b**. For this reaction, 1.5 equivalents of AIBN as a radical initiator and 3.0 equivalents of n-Bu_3_SnH as a chain transfer agent and 1 equivalent of the starting material were used, in toluene at 80 °C for 16 h under an argon atmosphere, with yields of 48% and 51%. Initially, tri-*n*-butyltin radicals abstract the bromine atom from oxime ethers **3a** and **3b**, generating aryl radicals, which undergo 7-*exo-trig* cyclization on oxime carbon generating *N*-alkoxyaminyl radicals, which are subsequently reduced, resulting in the formation of dihydrobenzoxepines fused to pyridine **6a** and **6b** (Scheme 2). The mechanisms of similar processes have been studied and reported by our research group. In these studies, both the cyclization and reduction rate constants of *N*-alkoxyaminyl radicals were determined to be 1.0 × 10^8^ s^−1^ and 3.4 × 10^0^ M^−1^ s^−1^, respectively [29,30].

#### 2.1.3. Synthesis of Triazoles **7**

**Scheme 3:**
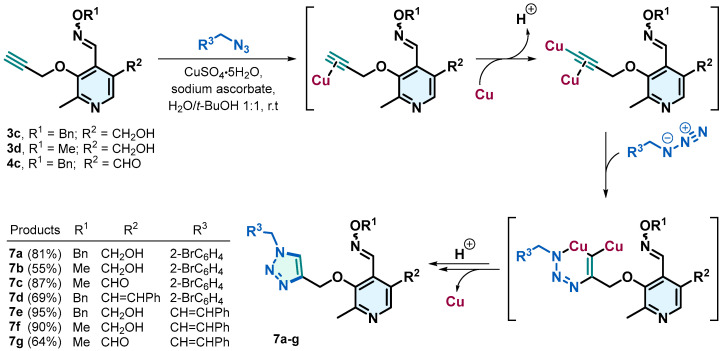
Copper(I)-catalyzed synthesis of 1,4-Disubstituted 1,2,3-Triazoles.

Due to the background and relevance of derivatives containing an azole group, we were interested in obtaining the triazoles **7a**–**7g** starting from compounds **3c**, **3d**, and **4c**, through azide-alkyne cycloadditions (Scheme 3). These reactions were carried out using sodium ascorbate as a reducing agent and the salt of Cu(II) as a catalyst in a water/*t*-BuOH mixture (Scheme 3). The presence of Cu(I), generated in situ from the Cu(II) salt, activates the terminal alkyne, facilitating its attack by the azide through a mechanism involving the stabilization of a metallacyclic intermediate and a strong polarization of the alkyne by Cu(I) [31]. This process allows the formation of a single product with 1,4-disubstituted regioselectivity, achieving yields between 55% and 95%.

#### 2.1.4. Synthesis of Triazoles **9**

**Scheme 4:**
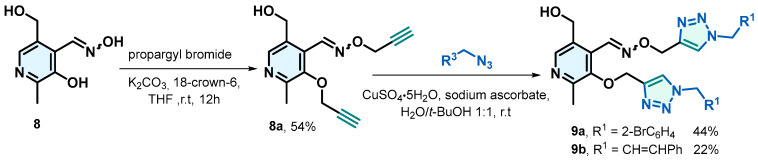
Synthesis of oxime ether **8a** and triazoles **9a** and **9b**.

Initially, the synthesis of **8a** was carried out starting from the free oxime of pyridoxal **8**, where the hydroxyl groups were alkylated with propargyl bromide using K_2_CO_3_ and 18-crown-6 ether in dry THF. After 12 h of stirring, compound **8a** was obtained with a yield of 54%. This compound was subjected to the same conditions previously described for the formation of triazoles **7**, yielding **9a** and **9b** with low yields of 44% and 22%, respectively (Scheme 4).

#### 2.1.5. Synthesis of Salicylaldehyde Derivatives **11**–**21**

**Scheme 5:**
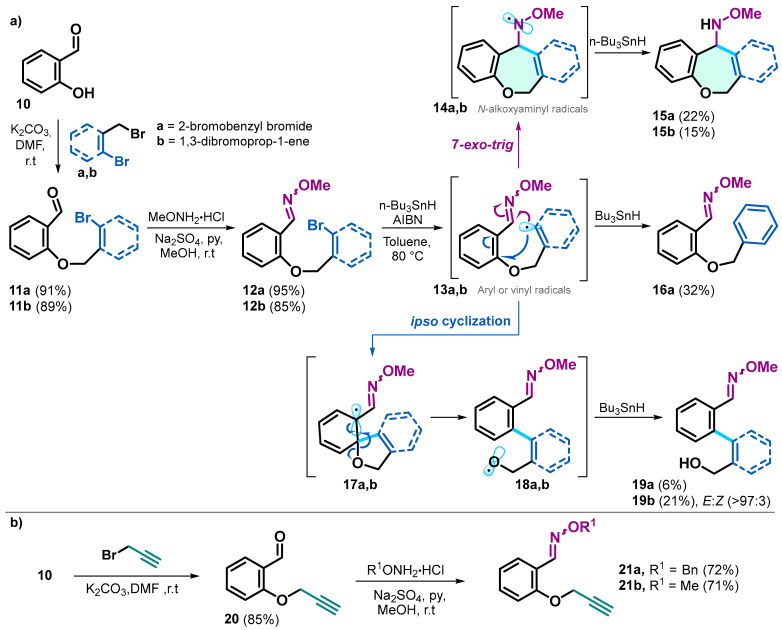
(**a**) synthetic route and radical reaction products of salicylaldehyde derivatives **12a** and **12b**. (**b**) synthesis of oxime ethers **21a** and **21b** derived from salicylaldehyde.

The synthesis of derivatives **11**–**21** was carried out following previously described methodologies [29]. Compounds **11a** and **11b** were obtained by reacting salicylaldehyde **10** with 2-bromobenzyl bromide or 1,3-dibromo-prop-1-ene in the presence of K_2_CO_3_ in DMF at room temperature. These compounds were then treated with *O*-methylhydroxylamine hydrochloride, Na_2_SO_4_, and pyridine in methanol at room temperature to yield compounds **12a** and **12b**. Subsequently, these compounds were treated with AIBN/n-Bu_3_SnH at 80 °C in toluene under an argon atmosphere to obtain derivatives **15a**, **15b**, **16a**, **19a**, and **19b**. The reaction of the brominated oxime ethers **12a** and **12b** with n-Bu_3_SnH and AIBN led to the generation of aryl and vinyl radicals, which underwent a 7-*exo-trig* cyclization, resulting in the formation of products **15a** and **15b**. Alternatively, reduction of the C–Br bond produced compound **16a**, while the reduced product derived from **12b** was observed in the reaction crude but could not be isolated. The formation of compounds **19a** and **19b** is attributed to the ring-opening of an intermediate ipso-cyclization, generated via a side reaction pathway (Scheme 5a).

On the other hand, the synthesis of **21** was carried out through an *O*-alkylation of **10** with propargyl bromide. Subsequently, this reaction product was condensed with *O*-benzylhydroxylamine hydrochloride or *O*-methylhydroxylamine hydrochloride to generate **21a** and **21b** (Scheme 5b).

### 2.2. In Vitro Antifungal Activity Against Strains of C. albicans, C. neoformans, and C. auris

**Figure 1:**
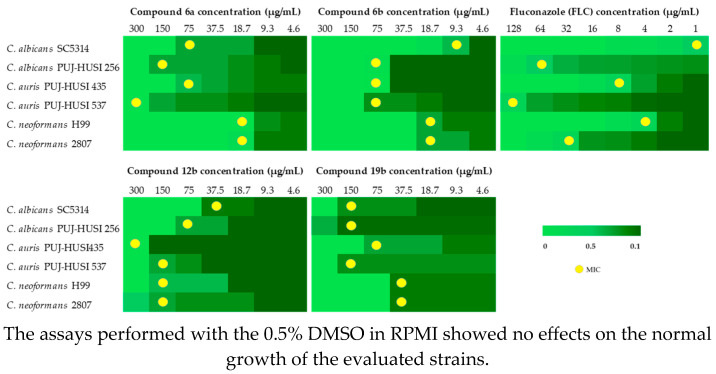
MIC values of compounds **6a**, **6b**, **12b**, and **19b**.

The in vitro antifungal activity of all synthesized compounds was evaluated against various yeast strains. The results indicated that compound **6a** exhibited the highest antifungal activity against *C. neoformans* H99 and 2807, with a MIC of 19 µg/mL in both cases. In contrast, compound **6b** displayed the best activity against *C. albicans* SC5314, with a MIC of 9.3 µg/mL. Similarly, the salicylaldehyde derivatives **12b** and **19b** also demonstrated notable antifungal activity: compound **12b** exhibited greater activity against *C. albicans* SC5314 with a MIC of 38 µg/mL, while compound **19b** showed a MIC of 38 µg/mL against *C. neoformans*. It is worth highlighting that compound **6b** exhibited a lower MIC compared to the reference drug FLC, with values of 75 µg/mL against *C. auris* PUJ-HUSI 537 and 19 µg/mL against *C. neoformans* 2807, whereas FLC showed values of 128 µg/mL and 32 µg/mL, respectively. Additionally, the data revealed that many of the evaluated compounds showed high minimum fungicidal concentration (MFC) values (see Supplementary Material), suggesting limited efficacy in eradicating fungal strains at the tested concentrations. In contrast, compounds **6a** and **6b** demonstrated a significant reduction in MFC, particularly against *C. neoformans* and *C. auris*, indicating their promising fungicidal potential (Figure 1).

Given the well-known biocidal activity of organotin (IV) compounds [32] and the potential presence of these compounds or their byproducts in derivatives obtained via radical reactions, the antifungal activity of n-Bu_3_SnH was evaluated, yielding MIC values below 4.6 µg/mL. To ensure that the observed antifungal activity was not influenced by residual organotin compounds, the purity of compounds **6a**, **6b**, **12b**, and **19b** was rigorously assessed using GC-MS, confirming the absence of n-Bu_3_SnH traces. Finally, the remaining derivatives exhibited no antifungal activity against the tested yeast strains at the evaluated concentrations (MIC ≥ 300 µg/mL).

### 2.3. Structure–Activity Relationships (SAR) Analysis

The antifungal activity evaluation revealed that various structural modifications to pyridoxal, including the transformation of the aldehyde group into an oxime ether, the alkylation of the hydroxyl group attached to the pyridine ring, the oxidation of the hydroxymethyl group to an aldehyde, the alkenylation at position 5, and the incorporation of 1,4-disubstituted 1,2,3-triazole moieties, did not produce any significant enhancement in antifungal activity. The activity remained comparable to that of unmodified pyridoxal, with MIC values exceeding 300 µg/mL. This suggests that these structural modifications do not play a pivotal role in improving the antifungal properties of the compounds.

**Figure 2:**
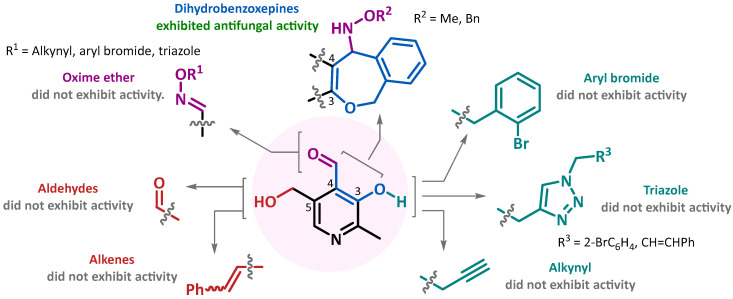
Summarization of structure–activity relationship (SAR) analysis for the pyridoxal derivates.

In contrast, the formation of a dihydrobenzoxepine nucleus fused to the pyridine ring led to a significant enhancement in antifungal activity. Compound **6a** exhibited a MIC of 19 µg/mL against *C. neoformans* 2807, while compound **6b** showed MIC values of 19 µg/mL against both *C. auris* and *C. neoformans*. Interestingly, the antifungal activity was notably higher when the alkoxyamine substituent in compounds **6** featured a methyl group, with a MIC of 75 µg/mL, rather than a benzyl group, also showing a MIC of 300 µg/mL, in the case of *C. auris* PUJ-HUSI 537 (Figure 2).

On the other hand, the antifungal activity of the salicylaldehyde-derived compounds **15a** and **15b** was compared with the pyridoxal-derived compounds **6a** and **6b** to evaluate the impact of the benzene versus the pyridine moiety. The MIC values for compounds **15a** and **15b** demonstrated that the absence of the pyridine moiety led to a significant reduction in antifungal activity. These findings underscore the crucial role of the benzoxepine-pyridine hybrid structure in compounds **6a** and **6b** for their observed antifungal efficacy.

This structural analysis provides valuable insights for researchers, serving as a guide for the discovery and optimization of compounds with antifungal potential. Additionally, the findings regarding *C. auris* and *C. neoformans* are noteworthy, as the MIC values for compounds **6a** and **6b** were lower than those for the reference drug, positioning them as promising candidates for developing novel antifungal therapies. Furthermore, this approach not only opens new avenues for future investigations but also highlights the antifungal potential of these fused cores and underlines the relevance of radical-based synthetic methods as tools for synthetizing innovative scaffolds.

### 2.4. Cytotoxic Activity

**Figure 3:**
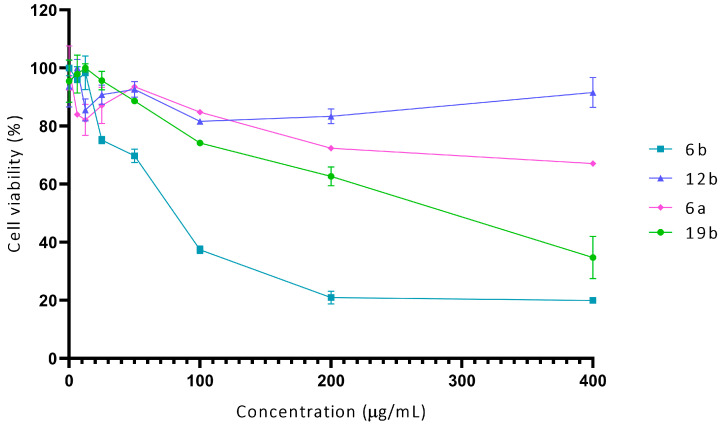
Cytotoxic effect of compounds **6a**, **6b**, **12b**, and **19b** against primary cell culture of fibroblasts.

To complement these studies, the cytotoxicity of compounds **6a**, **6b**, **12b**, and **19b** was evaluated in a human fibroblast primary cell culture using the MTT assay. The selected compounds were tested at concentrations up to 400 μg/mL for 2 h. Compound **6b** exhibited the highest toxicity, with an IC_50_ estimated at approximately 50–75 µg/mL. In contrast, compounds **6a** and **12b** showed minimal toxicity, maintaining over 65% cell viability even at 400 µg/mL, indicating no IC_50_ within the tested range. Compound **19b** displayed moderate toxicity, with an IC_50_ value of approximately 200 µg/mL (Figure 3). The results obtained with molecules **12b** and **6a** are comparable to the cytotoxicity of FLC reported in previous studies (500 µg/mL and 94% viability) under similar methodological conditions against fibroblasts [33].

### 2.5. ADMET Profile for Compounds ***6a***, ***6b***, ***12b***, and ***19b***

A computational analysis was performed using the SWISSADME web platform (http://www.swissadme.ch/index.php, accessed on 20 August 2024) to predict the pharmacokinetic properties of the most bioactive molecules **6a**, **6b**, **12b**, and **19b**. The analysis was based on Lipinski’s rule of five, which indicates a high potential for absorption and epithelial permeability [34]. Additional parameters were also evaluated, ensuring the molecules met complementary drug-likeness criteria, such as saturation (sp^3^-hybridized carbons) > 0.25, molecular flexibility (<9 rotatable bonds), lipophilicity (XLOGP3 values between −0.7 and +5.0), polarity or topological polar surface area (TPSA) within the range of 20–130 Å^2^, solubility (log S < 6), and molecular weight (50–500 g/mol). Notably, for compound **12b**, the fraction of sp^3^-hybridized carbons was calculated as 0.18, slightly below the recommended threshold. Nevertheless, this deviation was not regarded as critical, since the compound fulfilled all other evaluated parameters, further supporting its potential for future development (see Supplementary Material).

On the other hand, the boiled egg simulator indicated that all the evaluated compounds can penetrate the blood–brain barrier (BBB). Regarding their interaction with permeability glycoprotein (PGP), compounds **6a** and **6b** were classified as PGP^+^ substrates, indicating they are more easily expelled by cells. This feature potentially reduces their accumulation in target tissues, which may decrease therapeutic efficacy but also mitigate toxicity. In contrast, compounds **12b** and **19b** were identified as PGP^−^, suggesting a higher likelihood of retention in target tissues, potentially increasing therapeutic efficacy but also posing a greater risk of toxicity. Based on SWISSADME calculations, bioavailability radar plots indicated that these compounds exhibit favorable pharmacokinetic profiles and are likely to reach the CNS by BBB. Considering these findings and the urgent need for novel strategies and alternatives for the antifungal treatment of cryptococcosis, particularly meningitis, these compounds represent promising and innovative prototypes for further research in the development of antifungal therapies for cryptococcosis [12].

## 3. Materials and Methods

### 3.1. General Information

The melting points were determined in open capillary tubes on a digital fusiometer IA-9100 brand electrothermal. The reactions were monitored by TLC on 0.2 mm silica gel F_254_ plates (Merck, Milwaukee, WI, USA); the spots were visualized under UV light (254 nm). Flash chromatography column (FCC) was performed with silica gel (Merck with a pore size 60°A and 70–230 mesh). The chemical structures of intermediate and final products were elucidated by nuclear magnetic resonance spectra (^1^H NMR and ^13^C NMR) which were determined on a Bruker Avance spectrometer (Rheinstetten, Germany) 300 and 75 MHz/400 and 101 MHz. The spectra were recorded in a CDCl_3_ solution using a chloroform peak (7.26 ppm for ^1^H and 77.16 ppm for ^13^C) as a reference. High-resolution mass spectra (HRMS) were recorded on a Shimadzu (Columbia, MD, USA) LC-MS QTOF 9030 Nexera X2 system using electrospray ionization (ESI) (see Supplementary Materials).

### 3.2. Synthetic Procedures

#### 3.2.1. General Procedure for the Synthesis of Compounds **2a** and **2b**

To a mixture of pyridoxal hydrochloride (1.0 equiv.) and *O*-benzylhydroxylamine hydrochloride or methoxyamine hydrochloride (1.5 equiv.), a 10% aqueous NaHCO_3_ solution (3.0 equiv.) was added dropwise. The mixture was stirred at room temperature for 10 min. The resulting product (*E* and *Z* isomer mixture) was washed with water and filtered.

#### 3.2.2. General Procedure for the Synthesis of Compounds **3a**–**d**

A mixture of compounds **2a** or **2b** (1.0 equiv.) with 2-bromobenzyl bromide or propargyl bromide (1.5 equiv.), K_2_CO_3_ (1.5 equiv.), and 18-crown-6 ether (1.5 equiv.) in THF (0.25 M), under an argon atmosphere, was stirred at room temperature for 12 h. The reaction mixture was quenched with water, which was extracted with ethyl acetate, and the organic layer was dried over Na_2_SO_4_. The solvent was removed under reduced pressure, and the crude product was purified by FCC on silica gel (70–230 mesh) using hexane/ethyl acetate (7:3) as the eluent. This process yielded compounds **3a**–**d**.

#### 3.2.3. General Procedure for the Synthesis of Compounds **4a**–**d**

A mixture of compounds **3a** or **3d** (1.0 equiv.), with pyridinium chlorochromate (PCC) (1.5 equiv.), in 1,2-dichloroethane (0.20 M) was stirred and irradiated with microwaves for 1 min at a power of 100 W and a maximum temperature of 70 °C. The reaction mixture was quenched with water, which was extracted with ethyl acetate, and the organic layer was dried over Na_2_SO_4_. The solvent was removed under reduced pressure, and the crude product was purified by FCC on silica gel 70–230 mesh (hexane/acetate 7:3) to yield compounds **4a**–**d**.

#### 3.2.4. General Procedure for the Synthesis of Compounds **5a**–**c**

A mixture of compounds **4a**–**c** (1.0 equiv.), benzyltriphenylphosphonium chloride (1.5 equiv.), K_2_CO_3_ (1.5 equiv.), and a few drops of water (sufficient to dissolve the phosphonium salt) in 1,2-dichloroethane (0.70 M) was stirred and irradiated with microwaves for 1 min at 100 W, with a maximum temperature of 70 °C. Water was then added to the reaction mixture, which was extracted with ethyl acetate. The organic layer was dried over Na_2_SO_4_, and the solvent was removed under reduced pressure. The crude product was purified by FCC silica gel, 70–230 mesh using hexane/ethyl acetate (8:2) as the eluent, yielding compounds **5a**–**c**.

#### 3.2.5. General Procedure for the Synthesis of Compounds **6a** and **6b**

A flask was charged with **3a** or **3b** (1.0 equiv.), AIBN (1.5 equiv.), and n-Bu_3_SnH (3.0 equiv.) with toluene to reach a 0.10 M solution. The mixture was degassed with argon by cycles of freezing and thawing with liquid nitrogen, and the contents were placed into a mineral oil bath heated at 80 °C for 16 h. After removal of toluene under reduced pressure, the crude product was purified by FCC on silica gel 230–450 mesh (hexane/toluene 9:1) to generate compounds **6a** and **6b**. The purity of the compounds was verified by GC-MS to rule out the presence of trace amounts of organotin compounds.

#### 3.2.6. General Procedure for the Synthesis of Compounds **7a**–**g**

A mixture of compounds **3c**, **3d** or **4c** (1.0 equiv.) with 1-(azidomethyl)-2-bromobenzene or (*E*)-3-phenyl-2-propenyl-azide (1.5 equiv.), sodium ascorbate (0.1 equiv.), and CuSO_4_·5H_2_O (0.01 equiv.) in a water/*t*-BuOH 1:1 solution (0.05 M) was stirred at room temperature for 15 h. Water was then added to the reaction mixture and extracted with ethyl acetate, and the organic layer was dried over Na_2_SO_4_. The solvent was removed under reduced pressure, and the crude product was purified by FCC on silice gel, 70–230 mesh (hexane/acetate 7:3), to yield the compounds **7a**–**g**.

#### 3.2.7. General Procedure for the Synthesis of Compound **8a**

A mixture of compound **8** (1.0 equiv.) with propargyl bromide (2.4 equiv.), K_2_CO_3_ (2.4 equiv.), and 18-crown-6 ether (2.4 equiv.), in dry THF (0.25 M), under an argon atmosphere was stirred at room temperature for 12 h. The reaction mixture was quenched with water, which was extracted with ethyl acetate, and the organic layer was dried over Na_2_SO_4_. The solvent was removed under reduced pressure, and the crude product was purified by FCC on silica gel, 70–230 mesh (hexane/acetate 7:3), to yield the compound **8a**.

#### 3.2.8. General Procedure for the Synthesis of Compounds **9a** and **9b**

A mixture of compound **8a** (1.0 equiv.) with 1-(azidomethyl)-2-bromobenzene or (*E*)-3-phenyl-2-propenyl-azide (3.0 equiv.), sodium ascorbate (0.2 equiv.), and CuSO_4_·5H_2_O (0.02 equiv.) in a water/t-BuOH 1:1 solution (0.05 M) was stirred at room temperature for 16 h. Water was then added to the reaction mixture, which was extracted with ethyl acetate, and the organic layer was dried over Na_2_SO_4_. The solvent was removed under reduced pressure, and the crude product was purified by FCC on silica gel, 70–230 mesh (hexane/acetate 7:3), to yield the compounds **9a** and **9b**.

#### 3.2.9. General Procedure for the Synthesis of Compounds **11a** and **11b**

A mixture of **10** (1.0 equiv.), 2-bromobenzyl bromide or 1,3-dibromoprop-1-ene (1.2 equiv.), and K_2_CO_3_ (1.2 equiv.) in DMF (1.0M) was stirred at room temperature for 2 h. Subsequently, the reaction mixture was quenched with water and extracted with CH_2_Cl_2_. The organic phase was dried over Na_2_SO_4_, the solvent was removed under reduced pressure, and the purification by FCC on silica gel 70–230 mesh (hexane/dichloromethane 1:1) yielded compounds **11a** and **11b**.

#### 3.2.10. General Procedure for the Synthesis of Compounds **12a** and **12b**

A mixture of 11a or 11b (1.0 equiv.), O-methylhydroxylamine hydrochloride (1.1 equiv.), Na_2_SO_4_ (1.4 equiv.), and pyridine (2.0 equiv.) in MeOH (0.5M) was stirred for 12 h at room temperature. Subsequently, extractions were performed with water/dichloromethane 1:2. The organic phases were dried over Na_2_SO_4_ and evaporated under reduced pressure. Purification by FCC on silica gel 70–230 mesh (hexane/dichloromethane 7:3) yielded the compounds **12a** and **12b**.

#### 3.2.11. General Procedure for the Synthesis of Compounds **15a**, **15b**, **16a**, **19a,** and **19b**

A mixture of brominated oxime ether **12a** or **12b** (1.0 equiv.), n-Bu_3_SnH (3.0 equiv.), and AIBN (1.0 equiv.) in toluene (0.05 M) was degassed with argon through freeze-pump-thaw cycles with liquid nitrogen and placed in a mineral oil bath heated to 80 °C. The reaction mixture was stirred until the starting material was consumed (12 h). The reaction mixture was allowed to cool to room temperature, and the solvent was evaporated under reduced pressure. The crude reaction mixture from **12a** was purified by FCC (cyclohexane/ether 98:2), and the products **15a**, **16a**, and **19a** were isolated. The crude reaction mixture from **12b** was purified by preparative plate chromatography (silica gel, 1000 μm and 10 cm × 20 cm) with dichloromethane/hexane 4:6, isolating compounds **15b** and **19b**. The purity of the compounds was verified by GC-MS to rule out the presence of trace amounts of organotin compounds.

#### 3.2.12. General Procedure for the Synthesis of Compound **20**

A solution of 3-bromopropyne (1.5 equiv.) with 80 wt.% in toluene and **10** (1.0 equiv.) in DMF (0.50 M) was stirred at room temperature (1.5 equiv.) for 16 h, after which it was diluted with water and extracted with ethyl acetate. The organic extracts were dried with Na_2_SO_4_ and evaporated under reduced pressure. The solvent was removed under reduced pressure, and the crude product was purified by FCC on silice gel 70–230 mesh (hexane/dichloromethane 7:3) yielding the compound **20**.

#### 3.2.13. General Procedure for the Synthesis of Compounds **21a** and **21b**

A mixture of **20** (1.0 equiv.) with (1.1 equiv.) *O*-benzylhydroxylamine hydrochloride or methoxyamine hydrochloride (1.4 equiv.) was stirred at room temperature in methanol and then was added Na_2_SO_4_ (1.4 equiv.) and pyridine (2.0 equiv.) for 4 h, after which it was diluted with water and extracted with ethyl acetate. The organic extracts were dried with Na_2_SO_4_ and evaporated under reduced pressure. The solvent was removed under reduced pressure, and the crude product was purified by FCC on silice gel 70–230 mesh (hexane/dichloromethane 9:1) to yield the compounds **21a** and **21b**.

### 3.3. Antifungal Activity

#### 3.3.1. Fungal Strains

The reference strain *C. albicans* ATCC SC5314 and the clinical isolates, *C. albicans* 256-PUJ-HUSI (FLC-resistant), *C. auris* 435-PUJ-HUSI (antifungal sensitive), and *C. auris* 537-PUJ-HUSI (FLC and amphotericin B (AmB)-resistant), were obtained from the strain bank of the Micosis Humanas y Proteómica (MICOH-P) research group [4,35]. Subsequently, strains were grown on Sabouraud dextrose agar (SDA, Difco brand) plates and incubated overnight at 37 °C and stored at 4 °C for further use. The reference strain var. serotype A H99 (H99W) was obtained from the Madhani 2007 collection (http://www.fgsc.net, accessed on 10 March 2023). The clinical isolate (strain 2807) was obtained from the collection of the Microbiology Group of the National Institute of Health in Colombia. Subsequently, strains were grown on Sabouraud dextrose agar (SDA, Difco brand) plates and incubated 24–48 h at 37 °C and stored at 4 °C for further use.

#### 3.3.2. Antifungal Activity Assays

MICs were determined according to the guidelines of the Clinical Laboratory Standards Institute (CLSI BMD-M27-A3) with modifications [4,35]. The molecules were dissolved in dimethyl sulfoxide (DMSO), and later a serial dilution was made in Roswell Park Memorial Institute 1640 medium (4.6 to 300 µg/mL) in a 96-well microdilution plate (100 µL). Subsequently, 100 µL of the adjusted inoculum (0.5–2.5 × 10^3^ cells/mL) was added and incubated at 37 °C; past this time, visual and spectrophotometric reading (595 nm) was performed at 48 h (72 h). The controls used were as follows: growth control in DMSO, a known antifungal (FLC 1 to 64 µg/mL), and RPMI, as a sterility control. The MIC was defined as the minimum concentration of treatment that inhibited 50% of growth compared to the growth control. To determine the MFC, from the concentrations where no growth was observed, including the controls, a subculture was made on SA and incubated for 24 h at 37 °C. The MFC was defined as the minimum concentration of treatment that eliminated 100% of the growth compared to the control (*n* = 3).

### 3.4. Viability Test by Means of MTT (3-(4,5-dimethylthiazol-2-yl)-2,5-diphenyltetrazolium Bromide)

Cytotoxicity assays were performed using the MTT assay that allows the measurement of cellular metabolic activity as an indicator of cell viability. This assay was performed as previously described [36]. Briefly, human fibroblast primary cell culture (100 µL and 2.5 × 10^3^ cells/well) were seeded in 96-well plates at 37 °C in a 5% CO_2_-humidified atmosphere for 12 h, allowing for cell adhesion. After cell adherence, the culture medium was removed, and the cells were treated with the synthesized compounds **6a**, **6b**, **12b**, and **19b** for 2 h at 37 °C, and cell viability was determined using the MTT assay. For this, 10 µL of MTT solution (5 mg. mL^−1^) was added to each well, and the plates were incubated for 4 h at 37 °C. The formazan crystals were dissolved in DMSO (100 µL), and the absorbance (570 nm) was recorded on a Bio-Rad, Hercules, CA, USA, 680 microplate reader. The negative control was as follows: cells incubated with medium, (*n* = 3).

### 3.5. In Silico ADMET Analysis

Several medicinal chemistry parameters were obtained using the web platform http://www.swissadme.ch, accessed on 20 August 2024. The physicochemical properties of the compounds exhibiting the best activities were calculated, and predictive models suggested their potential viability as drug candidates. Additionally, the platform allowed the generation of radar plots to observe their biodisponibility. The properties that a compound must fulfill are as follows: (1) lipophilicity, XLOGP3 [−0.7, +5.0]; (2) molecular weight [150–500] (g/mol); (3) polarity or topological polar surface area (TPSA) [20, 130] Å^2^; (4) solubility (log S) < 6; (5) saturation (sp^3^-hybridized carbons) > 0.25; and (6) flexibility < 9 rotatable bonds.

## 4. Conclusions

A series of pyridoxal and salicylaldehyde derivatives were synthesized and evaluated against various strains of *C. albicans*, *C. auris*, and *C. neoformans.* Compounds **6a** and **6b**, obtained through 7-*exo-trig* radical cyclization reactions, exhibited superior activity against the drug-resistant strains of *C. auris* PUJ-HUSI 537 and *C. neoformans* 2807 compared to fluconazole. The fused dihydrobenzoxepine-pyridine scaffold was identified as a critical structural feature for enhancing antifungal efficacy, as confirmed by structure–activity relationship analysis. Additionally, in silico ADMET profiling revealed favorable pharmacokinetic properties, including blood–brain barrier penetration, supporting their potential for treating fungal infections of the central nervous system. Cytotoxicity assays confirmed the low toxicity of compound **6a** at the tested concentrations, further reinforcing its safety profile. These results highlight the potential of the fused dihydrobenzoxepine-pyridine scaffold as a promising antifungal candidate for further investigations.

## Data Availability

The original contributions presented in the study are included in the Article/Supplementary Materials; further inquiries can be directed at the corresponding authors.

## Supplementary Materials

The following supporting information can be downloaded at: https://www.mdpi.com/article/10.3390/molecules30051165/s1. Spectroscopic data and references are available. Table S1. In vitro antifungal activity of pyridoxal derivatives against *Candida* spp. and *C. neoformans*. Table S2. In vitro antifungal activity of salicylaldehyde derivatives against *Candida* spp. and *C. neoformans*. Figure S1. Bioavailability radars for the compounds that showed activity. Figure S2. The BOILED-EGG chart for the studied compounds. Refs. [29,37] are cited in the Supplementary Materials.
