# Supplementary material for: Pyridoxal and Salicylaldehyde Derivatives: Synthesis, Characterization, and Antifungal Potential Against Opportunistic Yeast Pathogens
Source: Molecules. 2025 Mar 5;30(5):1165. doi: 10.3390/molecules30051165 (PMC11901757; doi:10.3390/molecules30051165)
Supplement: Supplementary file 1 [file molecules-30-01165-s001.zip › molecules-3480259-supplementary.pdf]

Pyridoxal and Salicylaldehyde Derivatives: Synthesis, Characterization, and Antifungal Potential Against Opportunistic Yeast Pathogens

Jairo Camacho[1,2], Carlos A. Bejarano[2], John E. Diaz[2], Yerly Vargas-Casanova[3,4], Silvia Katherine Carvajal[3], Valentina Diaz Santoyo[3], Claudia M. Parra-Giraldo[1,5], Alix E. Loaiza[1]*

[1] Departamento de Química, Facultad de Ciencias, Pontificia Universidad Javeriana Cra. 7 No. 40-62, 110231, Bogotá, Colombia E-mail: aloaiza@javeriana.edu.co

[2] Departamento de Química, Facultad de Ciencias, Universidad Antonio Nariño Cra. 3 Este No. 47 A-15, 110231, Bogotá, Colombia

[3] Departamento de Microbiología, Facultad de Ciencias, Pontificia Universidad Javeriana Cra. 7 No. 40-62, 110231, Bogotá, Colombia

[4] Facultad de Ingeniería y Ciencias Básicas, Instituto Tecnológico del putumayo, Mocoa 860001, Colombia

[5] Departamento de Biomedicina, Facultad de Ciencias de la Salud y biociencias, Universidad Europea de Madrid, 28670 Madrid, España

Supplementary Material

Table of contents

1. Spectroscopic data 2
2. Copies of NMR spectra 7
3. Antifungal activity assays 35
4. ADMET analysis 36

References 36

1. Spectroscopic data

(*E*)-3-hydroxy-5-(hydroxymethyl)-2-methylisonicotinaldehyde *O*-benzyl oxime (2a)
White solid (989.0 mg, 99%), m.p. 138-139 °C. ^{1}H NMR (400 MHz, $CDCl_3$) δ (ppm): 8.70 (s, 1H), 7,85 (s, 1H), 7.43- 7.35 (m, 5H), 5.23 (s, 2H), 4.68 (s, 2H), 2.48 (s, 3H). ^{13}C NMR (101 MHz, $CDCl_3$) δ (ppm) 151.1, 149.4, 148.6, 138.8, 136.0, 131.3, 128.8, 128.7, 119.7, 77.6, 60.7, 19.0. HRMS (ESI) calcd. for $C_{15}H_{16}N_2O_3$ (M + H^+): 273.12337, found: 273.12062.

(*E*)-3-hydroxy-5-(hydroxymethyl)-2-methylisonicotinaldehyde *O*-methyl oxime (2b)
Solid beige (968.0 mg, 91%), m.p. 153-154 °C. ^{1}H NMR (300 MHz, $CDCl_3$) δ (ppm): 8.65 (s, 1H), 7.89 (s, 1H), 4.71 (s, 2H), 4.05 (s, 3H), 2.52 (s, 3H). ^{13}C NMR (75 MHz, $CDCl_3$) δ (ppm): 151.1, 149.8, 148.3, 139.3, 130.9, 119.4, 63.2, 60.9, 19.3. HRMS (ESI) calcd. for $C_9H_{12}N_2O_3$ (M + H^+): 197.09207, found: 197.09000.

(*E*)-3-((2-bromobenzyl)oxy)-5-(hydroxymethyl)-2-methylisonicotinaldehyde *O*-benzyl oxime (3a)
Solid beige (2100.0 mg, 80%), m.p. 121-122 °C. ^{1}H NMR (400 MHz, $CDCl_3$) δ (ppm): 8.52 (s, 1H), 8.35 (s, 1H), 7.60 (dd, *J* = 8.0, 1.2 Hz, 1H), 7.48 (dd, *J* = 7.7, 1.8 Hz 1H), 7.40-7.33 (m, 6H), 7.22 (td, *J* = 7.7, 1,8 Hz, 1H), 5.22 (s, 2H), 4.92 (s, 2H), 4.51, (s, 2H), 2,56 (s, 3H). ^{13}C NMR (101 MHz, $CDCl_3$) δ (ppm): 153.5, 151.9, 145.8, 145.2, 136.6, 135.3, 133.1, 132.8, 131.9, 130.2, 130.0, 128.8, 128.7, 128.6, 127.9, 123.3, 77.3, 75.9, 61.6, 19.4. HRMS (ESI) calcd. for $C_{20}H_{21}BrN_2O_3$ (M + H^+): 441.08083, found: 441.08013.

(*E*)-3-((2-bromobenzyl)oxy)-5-(hydroxymethyl)-2-methylisonicotinaldehyde *O*-methyl oxime (3b)
Solid beige (550.0 mg, 94%), m.p. 148-149 °C. ^{1}H NMR (400 MHz, $CDCl_3$) δ (ppm): 8.45 (s, 1H), 8.39 (s, 1H), 7.62 (dd, *J* = 7.9, 1.3 Hz, 1H), 7.49 (dd, *J* = 7.7, 1.7 Hz, 1H), 7.37 (td, *J* = 7.5, 1.3 Hz, 1H), 7.28-7.23 (m, 1H), 4.96 (s, 2H), 4.62, (s, 2H), 4.02 (s, 3H) 2,58 (s, 3H). ^{13}C NMR (101 MHz, $CDCl_3$) δ (ppm): 153.8, 151.7, 146.3, 144.7, 135.4, 133.1, 132.5, 131.7, 130.2, 130.1, 127.9, 123.4, 75.9, 62.9, 61.6, 19.7. HRMS (ESI) calcd. for $C_{16}H_{17}N_2O_3$ (M + H^+): 365.04953, found: 365.04902.

(*E*)-5-(hydroxymethyl)-2-methyl-3-(prop-2-yn-1-yloxy)isonicotinaldehyde *O*-benzyl oxime (3c)
Solid beige (1280.0 mg, 62%), m.p. 153-154 °C. ^{1}H NMR (300 MHz, $CDCl_3$) δ (ppm): 8.62 (s, 1H), 8.31 (s, 1H), 7.49 – 7.30 (m, 5H), 5.26 (s, 2H), 4.55 (d, *J* = 2.5 Hz, 2H), 4.51 (s, 2H), 2.55 (s, 3H), 2.45 (t, *J* = 2.5 Hz, 1H). ^{13}C NMR (75 MHz, $CDCl_3$) δ 153.6, 151.2, 146.4, 145.9, 136.6, 132.4, 132.0, 128.8, 128.7, 128.7, 77.5, 77.3, 61.9, 61.6, 19.8. HRMS (ESI) calcd. for $C_{18}H_{18}N_2O_3$ (M + H^+): 311.13902, found: 311.13906.

(*E*)-5-(hydroxymethyl)-2-methyl-3-(prop-2-yn-1-yloxy)isonicotinaldehyde *O*-methyl oxime (3d)
Solid beige (304.0 mg, 61%), m.p. 129-130 °C. ^{1}H NMR (300 MHz, $CDCl_3$) δ (ppm): 8.57 (s, 1H), 8.36 (s, 1H), 4.61 (d, *J* = 7.5 Hz, 2H), 4.59 (d, *J* = 2.4 Hz, 2H), 4.08 (s, 3H), 2.59-2.54 (m, 4H). ^{13}C NMR (75 MHz, $CDCl_3$) δ (ppm): 153.7, 151.3, 146.5, 145.2, 132.5, 132.0, 77.5, 77.4, 63.0, 61.9, 61.7, 19.9. HRMS (ESI) calcd. for $C_{12}H_{14}N_2O_3$ (M + H^+): 235.10772, found: 235.10774.

(*E*)-4-(((benzyloxy)imino)methyl)-5-((2-bromobenzyl)oxy)-6-methylnicotinaldehyde (4a)
Solid beige (1990.0 mg, 75%), m.p. 97-98 °C. ^{1}H NMR (400 MHz, $CDCl_3$) δ (ppm): 10.18 (s, 1H), 8.75 (s, 1H), 8.48 (s, 1H), 7.59 (dd, *J* = 8.0, 1.3 Hz, 1H), 7.48 (dd, *J* = 7.7, 1.7 Hz, 1H), 7.38 – 7.32 (m, 6H), 7.23 (td, *J* = 7.7, 1.8 Hz, 1H), 5.19 (s, 2H), 4.96 (s, 2H), 2.60 (s, 3H). ^{13}C NMR (101 MHz, $CDCl_3$) δ (ppm): 191.1, 159.1, 151.1, 145.5, 142.4, 136.7, 135.2, 133.6, 133.1, 130.2, 130.0, 128.8, 128.7, 128.6, 128.5, 127.9, 123.2, 77.5, 75.8, 20.2. HRMS (ESI) calcd. for $C_{22}H_{19}BrN_2O_3$ (M + H^+): 439.06518, found: 439.06607.

(*E*)-5-((2-bromobenzyl)oxy)-4-((methoxyimino)methyl)-6-methylnicotinaldehyde (4b)
Pale yellow oil (158.6mg, 76%). ^{1}H NMR (300 MHz, $CDCl_3$) δ (ppm): 10.35 (s, 1H), 8.77 (s, 1H), 8.40 (s, 1H), 7.61 (dd, *J* = 7.9, 1.3 Hz, 1H), 7.51 (dd, *J* = 7.7, 1.8 Hz, 1H), 7.38 (td, *J* = 7.5, 1.3 Hz, 1H), 7.26, (td, *J* = 7.7, 1.8, Hz 1H), 4.99 (s, 2H), 3.97 (s, 3H), 2.62 (s, 3H). ^{13}C NMR (75 MHz, $CDCl_3$) δ (ppm): 191.2, 159.3, 150.0, 145.6, 141.8, 135.2, 133.6, 133.1, 130.3, 130.1,

128.6, 127.9, 123.3, 75.8, 63.2, 20.3. HRMS (ESI) calcd. for $C_{16}H_{15}BrN_2O_3$ (M + H^+): 363.03388, found: 363.03464.

(*E*)-4-(((benzyloxy)imino)methyl)-6-methyl-5-(prop-2-yn-1-yloxy)nicotinaldehyde (4c)
Brown oil (315.0 mg, 79%). ^{1}H NMR (400 MHz, $CDCl_3$) δ (ppm): 10.20 (s, 1H), 8.73 (s, 1H), 8.58 (s, 1H), 7.43 – 7.27 (m, 5H), 5.25 (s, 2H), 4.57 (d, *J* = 2.5 Hz, 2H), 2.63 (s, 3H), 2.45 (t, *J* = 2.4 Hz, 1H). ^{13}C NMR (101 MHz, $CDCl_3$) δ (ppm): 191.1, 159.2, 150.3, 145.9, 142.9, 136.8, 133.7, 128.8, 128.7, 128.5, 128.5, 77.6, 77.6, 77.2, 61.7, 20.5. HRMS (ESI) calcd. for $C_{18}H_{16}N_2O_3$ (M + H^+): 309.12337, found: 309.12314.

(*E*)-4-((methoxyimino)methyl)-6-methyl-5-(prop-2-yn-1-yloxy)nicotinaldehyde (4d)
Brown oil (49.0 mg, 68%). ^{1}H NMR (300 MHz, $CDCl_3$) δ (ppm): 10.38 (s, 1H), 8.76 (s, 1H), 8.54 (s, 1H), 4.65 (d, *J* = 2.4 Hz, 2H), 4.06 (s, 3H), 2.66 (s, 3H), 2.57 (t, *J* = 2.4 Hz, 1H). ^{13}C NMR (75 MHz, $CDCl_3$) δ (ppm): 191.1, 159.3, 150.4, 145.9, 142.3, 133.8, 128.6, 77.7, 77.3, 63.3, 61.9, 20.5. HRMS (ESI) calcd. for $C_{12}H_{12}N_2O_3$ (M + H^+): 233.09207, found: 233.09194.

(*E*)-3-((2-bromobenzyl)oxy)-2-methyl-5-(*E*)-styryl)isonicotinaldehyde *O*-benzyl oxime (5a)
Pale yellow oil (584.4 mg, 75%). ^{1}H NMR (400 MHz, $CDCl_3$) δ (ppm): 8.53 (s, 1H), 8.43 (s, 1H), 7.48-7.53 (m, 2H), 7.46 (d, *J* = 16.3 Hz, 1H), 7.32 – 7.21 (m, 11H), 7.15 (td, *J* = 7.7, 1.8 Hz, 1H), 6.93 (d, *J* = 16.3 Hz, 1H), 5.12 (s, 2H), 4.86 (s, 2H), 2.45 (s, 3H). ^{13}C NMR (101 MHz, $CDCl_3$) δ (ppm): 151.9, 151.3, 144.8, 143.3, 137.4, 137.2, 135.8, 132.9, 132.1, 131.1, 130.3, 129.9, 129.7, 128.8, 128.6, 128.3, 128.2, 128.1, 127.8, 127.0, 124.8, 122.9, 76.8, 75.5, 19.4. HRMS (ESI) calcd. for $C_{29}H_{25}BrN_2O_2$ (M + H^+): 513.11722, found: 513.11723.

(*E*)-3-((2-bromobenzyl)oxy)-2-methyl-5-(*E*)-styryl)isonicotinaldehyde *O*-methyl oxime (5b)
Pale yellow oil. (316.0 mg, 76%). ^{1}H NMR (300 MHz, $CDCl_3$) δ (ppm): 8.63 (s, 1H), 8.41 (s, 1H), 7.66 (d, *J* = 16.4 Hz, 1H), 7.62 – 7.50 (m, 4H), 7.42 – 7.34 (m, 3H), 7.32 – 7.21 (m, 2H), 7.04 (d, *J* = 16.3 Hz, 1H), 4.96 (s, 2H), 3.99 (s, 3H), 2.56 (s, 3H). ^{13}C NMR (75 MHz, $CDCl_3$) δ (ppm): 151.9, 151.2, 144.0, 143.2, 137.3, 135.8, 132.9, 131.9, 131.0, 130.3, 129.9, 129.8, 128.9, 128.2, 127.8, 126.9, 124.9, 122.9, 75.5, 62.7, 19.5. HRMS (ESI) calcd. for $C_{23}H_{21}BrN_2O_2$ (M + H^+): 481.07684, found: 481.07751.

(*E*)-2-methyl-3-(prop-2-yn-1-yloxy)-5-(*E*)-styrylisonicotinaldehyde *O*-benzyl oxime (5c)
Pale yellow solid (60.2 mg, 79%), m.p. 74-75 °C. ^{1}H NMR (300 MHz, $CDCl_3$) δ (ppm): 8.59 (s, 1H), 8.56 (s, 1H), 7.54 (d, *J* = 16.3 Hz, 1H), 7.42 – 7.28 (m, 10H), 6.99 (d, *J* = 16.3 Hz, 1H), 5.27 (s, 2H), 4.55 (d, *J* = 2.4 Hz, 2H), 2.58 (s, 3H), 2.50 (t, *J* = 2.4 Hz, 1H). ^{13}C NMR (101 MHz, $CDCl_3$) δ (ppm): 152.0, 150.6, 145.1, 143.6, 137.5, 137.1, 132.1, 131.0, 130.4, 128.9, 128.7, 128.4, 128.2, 128.2, 127.0, 124.9, 77.9, 77.0, 76.9, 61.7, 19.7. HRMS (ESI) calcd. for $C_{25}H_{22}N_2O_2$ (M + H^+): 383.17540, found: 383.17537.

(5-((benzyloxy)amino)-1-methyl-5,10-dihydrobenzo[5,6]oxepino[2,3-*c*]pyridin-4-yl)methanol (6a)
Yellow oil. (35.0 mg, 48%). ^{1}H NMR (300 MHz, $CDCl_3$) δ (ppm): 8.00 (s, 1H), 7.39 – 7.33 (m, 3H), 7.28 – 7.26 (m, 3H), 7.19 (dd, *J* = 7.1, 2.0 Hz, 1H), 7.16 – 7.11 (m, 2H), 6.11 (d, *J* = 13.5 Hz, 1H), 5.34 (s, 1H), 4.89 (d, *J* = 10.2 Hz, 1H), 4.84 (d, *J* = 9.0 Hz, 1H), 4.62 (d, *J* = 12.3 Hz, 1H), 4.53 – 4.43 (m, 2H), 2.44 (s, 3H). ^{13}C NMR (75 MHz, $CDCl_3$) δ (ppm): 152.6, 152.4, 142.3, 137.3, 136.5, 135.9, 133.3, 133.1, 131.7, 129.1, 128.9, 128.5, 128.4, 128.1, 127.9, 76.9, 71.2, 64.3, 61.5, 19.7. HRMS (ESI) calcd. for $C_{22}H_{22}N_2O_3$ (M + H^+): 363.17032, found: 363.17057.

(5-(methoxyamino)-1-methyl-5,10-dihydrobenzo[5,6]oxepino[2,3-*c*]pyridin-4-yl)methanol (6b)
Yellow oil. (40.0 mg, 51%). ^{1}HNMR (300 MHz, $CDCl_3$) δ (ppm): 8.04 (s, 1H), 7.43 – 7.40 (m, 1H), 7.35-7.30 (m, 2H), 7.19 (dd, *J* = 6.5, 2.3 Hz, 1H), 6.09 (d, *J* = 13.8 Hz, 1H), 5.37 (s, 1H), 4.95 (d, *J* = 13.8 Hz, 1H), 4.89 (d, *J* = 12.5 Hz, 1H), 4.69 (d, *J* = 12.5 Hz, 1H), 3.37 (s, 3H), 2.48 (s, 3H). ^{13}C NMR (75 MHz, $CDCl_3$) δ (ppm): 152.8, 151.8, 141.5, 136.3, 135.4, 135.0, 134.2, 131.8, 129.2, 128.7, 127.8, 71.6, 64.2, 62.6, 61.3, 19.1. HRMS (ESI) calcd. for $C_{16}H_{18}N_2O_3$ (M + H^+): 287.13902, found: 287.13907.

(*E*)-3-((1-(2-bromobenzyl)-1*H*-1,2,3-triazol-4-yl)methoxy)-5-(hydroxymethyl)-2-methylisonicotinaldehyde *O*-benzyl oxime (7a)
Pale pink solid (205.0 mg, 81%), m.p 92-93 °C. ^{1}H NMR (300 MHz, $CDCl_3$) δ (ppm): 8.51 (s, 1H), 8.29 (s, 1H), 7.62 (dd, *J* =

7.8, 1.5 Hz, 1H), 7.56 (s, 1H), 7.46 – 7.19 (m, 7H), 7.16 (dd, *J* = 7.4, 1.9 Hz, 1H), 5.66 (s, 2H), 5.23 (s, 2H), 4.98 (s, 2H), 4.48 (d, *J* = 5.4 Hz, 2H), 3.78 (bs, OH), 2.51 (s, 3H). ^{13}C NMR (75 MHz, $CDCl_3$) δ (ppm): 153.8, 151.7, 146.3, 145.5, 143.2, 136.5, 134.0, 133.4, 132.5, 131.5, 130.7, 130.6, 128.9, 128.7, 128.7, 128.5, 123.7, 123.5, 77.3, 67.8, 61.6, 54.1, 19.8. HRMS (ESI) calcd. for $C_{25}H_{24}BrN_5O_3$ (M + H$^+$): 522.11353, found: 522.11328.

(*E*)-3-((1-(2-bromobenzyl)-1*H*-1,2,3-triazol-4-yl)methoxy)-5-(hydroxymethyl)-2-methylisonicotinaldehyde *O*-methyl oxime (7b)

Pale pink solid (47.0 mg, 55%), m.p 129-130 °C. ^{1}H NMR (300 MHz, $CDCl_3$) ;δ (ppm): 8.44 (s, 1H), 8.32 (s, 1H), 7.65 – 7.56 (m, 2H), 7.37 – 7.13 (m, 3H), 5.67 (s, 2H), 4.99 (s, 2H), 4.57 (s, 2H), 4.01 (s, 3H), 2.51 (s, 3H). ^{13}C NMR (75 MHz, $CDCl_3$) δ (ppm): 153.6, 151.5, 146.2, 144.8, 143.1, 133.9, 133.4, 132.5, 131.5, 130.7, 130.6, 128.4, 123.6, 123.5, 67.6, 62.9, 61.5, 54.0, 19.7. HRMS (ESI) calcd. for $C_{19}H_{20}BrN_5O_3$ (M + H$^+$): 446.08223, found: 446.08207.

(*E*)-5-((1-(2-bromobenzyl)-1*H*-1,2,3-triazol-4-yl)methoxy)-4-((methoxyimino)methyl)-6-ethylnicotinaldehyde (7c)

Yellow oil (50.0 mg, 87%). ^{1}H NMR (300 MHz, $CDCl_3$) δ (ppm): 10.31 (s, 1H), 8.73 (s, 1H), 8.39 (s, 1H), 7.62 (dd, *J* = 7.8, 1.5 Hz, 1H), 7.60 (s, 1H), 7.32 (td, 7.4, 1.4 Hz, 1H), 7.28 – 7.23 (m, 1H), 7.19 (td, *J* = 7.8, 1.9 Hz, 1H), 5.67 (s, 2H), 5.06 (s, 2H), 3.99 (s, 3H), 2.59 (s, 3H). ^{13}C NMR (75 MHz, $CDCl_3$) δ (ppm): 191.2, 159.3, 152.6, 150.7, 145.8, 142.9, 142.0, 133.8, 133.4, 130.8, 130.6, 128.5, 128.4, 123.7, 123.6, 67.5, 63.3, 54.1, 20.4. HRMS (ESI) calcd. for $C_{19}H_{18}BrN_5O_3$ (M + H$^+$): 444.06658, found: 444.06613.

(*E*)-3-((1-(2-bromobenzyl)-1*H*-1,2,3-triazol-4-yl)methoxy)-2-methyl-5-((*Z*)-styryl)isonicotinaldehyde *O*-benzyl oxime (7d)

Yellow oil (64.0 mg, 69%). ^{1}H NMR (300 MHz, $CDCl_3$) δ (ppm): 8.33 (s, 1H), 8.10 (s, 1H), 7.62 (dd, *J* = 7.8, 1.4 Hz, 1H), 7.55 (s, 1H), 7.38 – 7.08 (m, 15H), 6.69 (d, *J* = 12.1 Hz, 1H), 6.59 (d, *J* = 12.1 Hz, 1H), 5.65 (s, 2H), 5.15 (s, 2H), 5.03 (s, 2H), 2.51 (s, 3H). ^{13}C NMR (75 MHz, $CDCl_3$) δ (ppm): 152.1, 150.4, 146.2, 144.4, 143.8, 137.3, 136.3, 134.0, 133.3, 132.1, 131.0, 130.9, 130.5, 130.3, 129.0, 128.7, 128.5, 128.4, 128.3, 128.2, 127.5, 125.9, 123.5, 123.4, 76.7, 67.1, 53.9, 19.6. HRMS (ESI) calcd. for $C_{32}H_{28}BrN_5O_2$ (M + H$^+$): 594.14991, found: 594.14960.

(*E*)-3-((1-cinnamyl-1*H*-1,2,3-triazol-4-yl)methoxy)-5-(hydroxymethyl)-2-methylisonicotinaldehyde *O*-benzyl oxime (7e)

Beige solid (48.1 mg, 95%). m.p. 101-102 °C. ^{1}H NMR (300 MHz, $CDCl_3$) δ (ppm): 8.39 (s, 1H), 8.18 (s, 1H), 7.50 (s, 1H), 7.31 – 7.14 (m, 10H), 6.56 (d, *J* = 15.8 Hz, 1H), 6.21 (dt, *J* = 15.8, 6.7 Hz, 1H), 5.09 (s, 2H), 5.01 (dd, *J* = 6.8, 1.4 Hz, 2H), 4.87 (s, 2H), 4.36 (s, 2H), 2.42 (s, 3H). ^{13}C NMR (75 MHz, $CDCl_3$) δ (ppm): 153.6, 151.5, 146.0, 145.3, 143.1, 136.5, 135.8, 135.3, 132.4, 131.4, 128.8, 128.7, 128.7, 128.6, 128.6, 126.8, 122.9, 121.5, 77.1, 67.7, 61.5, 52.5, 19.5. HRMS (ESI) calcd. for $C_{26}H_{25}N_5O_3$ (M + H$^+$): 456.20302, found: 456.19989.

(*E*)-3-((1-cinnamyl-1*H*-1,2,3-triazol-4-yl)methoxy)-5-(hydroxymethyl)-2-methylisonicotinaldehyde *O*-methyl oxime (7f)

Beige solid (60.6 mg, 90%). m.p. 115-116 °C. ^{1}H NMR (300 MHz, $CDCl_3$) δ (ppm): 8.43 (s, 1H), 8.32 (s, 1H), 7.64 (s, 1H), 7.42 – 7.28 (m, 5H), 6.69 (d, *J* = 15.8 Hz, 1H), 6.33 (dt, *J* = 15.8, 6.7 Hz, 1H), 5.15 (dd, *J* = 6.7, 1.4 Hz, 2H), 5.00 (s, 2H), 4.57 (s, 2H), 3.99 (s, 3H), 2.54 (s, 3H). ^{13}C NMR (75 MHz, $CDCl_3$) δ (ppm): 153.6, 151.5, 146.2, 144.7, 143.1, 135.9, 135.4, 132.5, 131.6, 128.9, 128.8, 126.8, 123.0, 121.5, 67.7, 62.8, 61.5, 52.6, 19.7. HRMS (ESI) calcd. for $C_{20}H_{21}N_5O_3$ (M + H$^+$): 380.17172, found: 380.16898.

(*E*)-5-((1-cinnamyl-1*H*-1,2,3-triazol-4-yl)methoxy)-4-((methoxyimino)methyl)-6-methylnicotinaldehyde (7g)

Yellow oil (38.6 mg, 64%). ^{1}H NMR (300 MHz, $CDCl_3$) δ (ppm): 10.32 (s, 1H), 8.73 (s, 1H), 8.38 (s, 1H), 7.67 (s, 1H), 7.40 – 7.31 (m, 5H), 6.71 (d, *J* = 16.3 Hz, 1H), 6.35 (dt, *J* = 15.8, 6.7 Hz, 1H), 5.18 (dd, *J* = 6.7, 1.4 Hz, 2H), 5.08 (s, 2H), 3.98 (s, 3H), 2.61 (s, 3H). ^{13}C NMR (75 MHz, $CDCl_3$) δ (ppm): 191.2, 159.3, 150.8, 145.7, 143.0, 142.0, 135.9, 135.3, 133.4, 128.9, 128.8, 128.5, 126.8, 123.1, 121.5, 67.6, 63.1, 52.6, 20.4. HRMS (ESI) calcd. for $C_{20}H_{19}N_5O_3$ (M + H$^+$): 378.15607 found: 378.15403.

(*E*)-5-(hydroxymethyl)-2-methyl-3-(prop-2-yn-1-yloxy)isonicotinaldehyde-*O*-prop-2-yn-1-yl oxime (8a)

Beige solid (38.5 mg, 54%). m.p. 96-97 °C. ^{1}H NMR (400 MHz, $CDCl_3$) δ (ppm): 8.64 (s, 1H), 8.39 (s, 1H), 4.83 (d, *J* = 2.4 Hz, 2H), 4.66 (s, 2H), 4.60 (d, *J* = 2.4 Hz, 2H), 2.61–2.57 (m, 5H). ^{13}C NMR (101 MHz, $CDCl_3$) δ (ppm): 153.4, 151.4, 146.4, 146.0, 132.8, 131.9, 78.5, 77.7, 77.3, 76.0, 62.4, 61.9, 61.6, 19.5. calcd. for $C_{14}H_{14}N_2O_3$ (M + H$^+$): 259.10772 found: 259.10425.

(*E*)-3-((1-(2-bromobenzyl)-1*H*-1,2,3-triazol-4-yl)methoxy)-5-(hydroxymethyl)-2-ethylisonicotionaldehyde-*O*-((1-(2-bromobenzyl)-1*H*-1,2,3-triazol-4-yl)methyl) oxime (9a)

Yellow oil (35.0 mg, 44%). ^{1}H NMR (400 MHz, $CDCl_3$) δ (ppm): 8.43 (s, 1H), 8.34 (s, 1H), 7.72 (s, 1H), 7.63 – 7.58 (m, 3H), 7.34 – 7.16 (m, 6H), 5.67 (s, 2H), 5.65 (s, 2H), 5.31 (s, 2H), 4.97 (s, 2H), 4.53 (s, 2H), 2.53 (s, 3H). ^{13}C NMR (101 MHz, $CDCl_3$) δ (ppm): 153.3, 151.7, 145.7, 145.6, 143.6, 142.9, 134.0, 133.9, 133.4, 133.4, 131.8, 131.4, 130.7, 130.7, 130.7, 130.6, 128.5, 128.4, 123.8, 123.7, 123.7, 123.7, 67.8, 67.7, 61.5, 54.0, 54.0, 19.4. HRMS (ESI) calcd. for $C_{28}H_{26}BrN_8O_3$ (M + H$^+$): 681.05674 found: 681.05634.

(*E*)-3-((1-cinnamyl-1*H*-1,2,3-triazol-4-yl)methoxy)-5-(hydroxymethyl)-2-ethylisonicotinaldehyde *O*-((1-cinnamyl-1*H*-1,2,3-triazol-4-yl)methyl) oxime (9b)

Yellow oil (20.0 mg, 22%). ^{1}H NMR (400 MHz, $CDCl_3$) δ 8.43 (s, 1H), 8.30 (s, 1H), 7.73 (s, 1H), 7.63 (s, 1H), 7.42 – 7.23 (m, 10H), 6.68 (dd, *J* = 15.8, 7.4 Hz, 2H), 6.33 (dtd, *J* = 15.9, 6.7, 2.6 Hz, 2H), 5.31 (s, 2H), 5.13 (dd, *J* = 6.7, 1.4 Hz, 4H), 4.96 (s, 2H), 4.54 (s, 2H), 2.54 (s, 3H). ^{13}C NMR (101 MHz, $CDCl_3$) δ (ppm): 153.5, 151.6, 146.1, 145.7, 143.7, 143.0, 136.0, 135.9, 135.8, 135.5, 132.7, 131.6, 128.9, 128.9, 128.8, 128.7, 126.9, 126.8, 123.3, 123.2, 121.7, 121.6, 67.8, 67.7, 61.5, 52.6, 52.6, 19.6. HRMS (ESI) calcd. for $C_{32}H_{32}BrN_8O_3$ (M + H$^+$): 577.26701 found: 577.26606.

2-((2-bromobenzyl)oxy)benzaldehyde (11a)

White solid (1085.0 mg, 91%). m.p. 91-92 °C. NMR data were consistent with those previously published[1]. ^{1}H NMR (300 MHz, $CDCl_3$) δ (ppm): 10.59 (s, 1H), 7.88 (dd, *J* = 7.5, 1.7 Hz, 1H), 7.62 (dd, *J* = 8.0, 1.3 Hz, 1H), 7.60 – 7.53 (m, 2H), 7.37 (td, *J* = 7.6, 1.3 Hz, 1H), 7.24 (td, *J* = 7.7, 1.7 Hz, 1H), 7.12 – 7.04 (m, 2H), 5.26 (s, 2H). ^{13}C NMR (75 MHz, $CDCl_3$) δ (ppm): 189.4, 160.6, 135.9, 135.3, 132.8, 129.6, 128.8, 128.7, 127.7, 125.3, 122.3, 121.2, 113.0, 69.9.

***(E,Z)* 2-(3-bromoallyloxy)benzaldehyde (11b)**

Pale yellow oil (878.0 mg, 89%). NMR data were consistent with those previously published[1]. ^{1}H NMR (300 MHz, $CDCl_3$), *E,Z* isomers: δ (ppm): 10.51 (s, 2H), 7.88 (d, *J* = 1.9 Hz, 1H), 7.86 (d, *J* = 1.8 Hz, 1H), 7.61–7.52 (m, 2H), 7.12–7.04 (m, 2H), 7.01 (dd, *J* = 8.4, 0.9 Hz, 1H), 6.97 (dd, *J* = 8.5, 0.9 Hz, 1H), 6.62 – 6.44 (m, 4H), 4.90 – 4.82 (m, 2H), 4.64 (d, *J* = 1.2 Hz, 1H), 4.62 (d, *J* = 1.2 Hz, 1H). 13C NMR (75 MHz, CDCl3), *E,Z* isomers: δ (ppm): 189.7, 189.5, 160.5, 160.3, 136.0, 135.9, 131.7, 130.2, 128.8, 128.6, 125.1, 125.1, 121.4, 121.2, 112.7, 112.6, 110.8, 110.2, 68.0, 66.7.

***(E)*-2-((2-bromobenzyl)oxy)benzaldehyde *O*-methyl oxime (12a)**

White solid (132.0 mg, 95%). m.p. 75–76 °C. NMR data were consistent with those previously published[1]. ^{1}H NMR (300 MHz, $CDCl_3$) δ (ppm): 8.57 (s, 1H), 7.85 (dd, *J* = 7.7, 1.8 Hz, 1H), 7.62 (dd, *J* = 7.9, 1.3 Hz, 1H), 7.56 (dd, *J* = 7.7, 1.7 Hz, 1H), 7.40– 7.32 (m, 2H), 7.23 (td, *J* = 7.7, 1.8 Hz, 1H), 7.05 – 6.95 (m, 2H), 5.18 (s, 2H), 4.01 (s, 3H). ^{13}C NMR (75 MHz, $CDCl_3$) δ (ppm): 156.3, 144.7, 135.9, 132.7, 131.1, 129.4, 128.9, 127.6, 126.7, 122.3, 121.4, 121.1, 112.5, 69.8, 62.0.

(*E*)-2-(((*E,Z*)-3-bromoallyl)oxy)benzaldehyde *O*-methyl oxime (12b)

Pale lilac oil (382.0 mg, 85%). NMR data were consistent with those previously published[1]. ^{1}H NMR (300 MHz, $CDCl_3$) δ (ppm): 8.49 (s, 1H), 8.47 (s, 1H), 7.86 – 7.79 (m, 2H), 7.40 – 7.30 (m, 2H), 7.00 (t, *J* = 7.5 Hz, 2H), 6.91 (d, *J* = 8.3 Hz, 1H), 6.87 (d, *J* = 8.3 Hz, 1H), 6.58 – 6.38 (m, 4H), 4.76 (d, *J* = 3.5 Hz, 2H), 4.52 (d, *J* = 5.0 Hz, 2H), 4.00 (s, 3H), 4.00 (s, 3H). ^{13}C NMR (75 MHz, $CDCl_3$). δ (ppm): 156.1, 156.0, 144.6, 144.5, 132.1, 132.1, 131.1, 131.0, 130.8, 126.8, 126.5, 121.5, 121.3, 121.1, 121.0, 112.2, 110.1, 109.6, 67.9, 66.6, 62.0, 61.9.

***N*-(6,11-dihydrodibenzo[*b*,*e*]oxepin-11-yl)-*O*-methylhydroxylamine (15a)**

Pale yellow oil (46.0 mg, 22%). NMR data were consistent with those previously published[1]. ^{1}H NMR (300 MHz, $CDCl_3$) δ (ppm): 7.41 – 7.21 (m, 6H), 6.99 (dd, *J* = 7.4, 1.3 Hz, 1H), 6.94 (dd, *J* = 8.0, 1.3 Hz, 1H), 6.31 (d, *J* = 12.9 Hz, 1H), 4.97 (s,

1H), 4.88 (d, *J* = 12.9 Hz, 1H), 3.29 (s, 3H). ^{13}C NMR (75 MHz, $CDCl_3$) δ (ppm): 158.2, 137.7, 136.5, 132.9, 130.6, 129.9, 128.6, 128.5, 128.4, 124.3, 121.7, 120.6, 71.13, 70.6, 62.6.

N-(2,5-dihydrobenzo[*b*]oxepin-5-yl)-*O*-methylhydroxylamine (15b)

Pale yellow oil (12.4 mg, 15%). NMR data were consistent with those previously published[1]. ^{1}H NMR (300 MHz, $CDCl_3$) δ (ppm): 7.33 – 7.25 (m, 1H), 7.23 (dd, *J* = 7.5, 1.9 Hz, 1H), 7.18 – 7.08 (m, 2H), 6.03 (ddt, *J* = 11.6, 7.0, 2.4 Hz, 1H), 5.73 – 5.66 (m, 1H), 4.80 – 4.66 (m, 1H), 4.57 (d, *J* = 7.0 Hz, ^{1}H), 4.49 – 4.38 (m, 1H), 3.58 (s, 3H). ^{13}C NMR (75 MHz, $CDCl_3$) δ (ppm): 157.3, 135.9, 131.1, 129.2, 129.1, 125.8, 124.4, 121.9, 70.8, 61.7, 61.6.

***(E)*-2-(benzyloxy)benzaldehyde *O*-methyl oxime (16a)**

Pale yellow oil (68.0 mg, 32%). NMR data were consistent with those previously published[1]. ^{1}H NMR (300 MHz, $CDCl_3$) δ (ppm): 8.58 (s, 1H), 7.87 (dd, *J* = 7.6, 1.8 Hz, 1H), 7.49 – 7.31 (m, 6H), 7.05 – 6.96 (m, 2H), 5.12 (s, 2H), 4.01 (s, 3H). ^{13}C NMR (75 MHz, $CDCl_3$) δ (ppm): 156.7, 144.8, 136.6, 131.1, 128.7, 128.0, 127.4, 126.5, 121.1, 112.5, 70.3, 62.0.

(*E*)-2'-(hydroxymethyl)-[1,1'-biphenyl]-2-carbaldehyde *O*-methyl oxime (19a)

Pale yellow oil (12.0 mg, 6%). NMR data were consistent with those previously published[1]. ^{1}H NMR (300 MHz, $CDCl_3$) δ (ppm): 8.01 – 7.95 (m, 1H), 7.76 (s, 1H), 7.60 – 7.55 (m, 1H), 7.47 – 7.40 (m, 3H), 7.36 (td, *J* = 7.5, 1.5 Hz, 1H), 7.27 – 7.23 (m, 1H), 7.16 (dd, *J* = 7.5, 1.5 Hz, 1H), 4.43 (s, 2H), 3.91 (s, 3H). ^{13}C NMR (75 MHz, $CDCl_3$) δ (ppm): 147.1, 140.2, 138.8, 138.0, 130.2, 130.1, 129.4, 128.3, 127.9, 127.8, 127.4, 125.6, 63.0, 61.9.

(1*E*)-2-((*E*,*Z*)-3-hydroxyprop-1-en-1-yl)benzaldehyde *O*-methyl oxime (19b)

Pale yellow oil (17.0 mg, 21%). NMR data were consistent with those previously published[1]. ^{1}H NMR (300 MHz, $CDCl_3$) δ (ppm): 8.42 (s, 1H), 7.74 (dd, *J* = 7.7, 1.6 Hz, 1H), 7.47 (dd, *J* = 7.7, 1.5 Hz, 1H), 7.36 (td, *J* = 7.5, 1.6 Hz, 1H), 7.33 – 7.26 (m, 2H), 6.99 (d, *J* = 15.8 Hz, 1H), 6.25 (dt, *J* = 15.7, 5.5 Hz, 1H), 4.37 (dd, *J* = 5.5, 1.7 Hz, 2H), 4.01 (s, 3H). ^{13}C NMR (75 MHz, $CDCl_3$) δ (ppm): 147.3, 136.3, 132.5, 129.8, 129.3, 127.7, 127.7, 127.1, 63.7, 62.1

(*E*)-2-(prop-2-yn-1-yloxy)benzaldehyde *O*-benzyl oxime (21a)

White solid (600.0 mg, 72%). m.p. 53-54 °C. ^{1}H NMR ^{1}H NMR (400 MHz, $CDCl_3$) δ (ppm): 8.45 (s, 1H), 7.72 (dd, *J* = 7.9, 1.8 Hz, 1H), 7.36 – 7.16 (m, 6H), 6.92 – 6.84 (m, 2H), 5.11 (s, 2H), 4.58 (d, *J* = 2.4 Hz, 2H), 2.39 (t, *J* = 2.4 Hz, 1H). ^{13}C NMR (101 MHz, $CDCl_3$) δ (ppm): 155.6, 145.0, 137.7, 131.0, 128.5, 128.5, 128.0, 126.7, 121.8, 121.7, 112.8, 78.3, 76.4, 76.0, 56.4. Other spectroscopic data were previously reported in the literature[2]

(*E*)-2-(prop-2-yn-1-yloxy)benzaldehyde *O*-methyl oxime (21b)

White solid (500.0 mg, 71%). m.p. 47-48 °C. ^{1}H NMR (400 MHz, $CDCl_3$) δ (ppm): 8.47 (s, 1H), 7.81 (dd, *J* = 8.1, 1.6 Hz, 1H), 7.34 (td, *J* = 8.1, 1.8 Hz, 1H), 7.00-6.93 (m, 2H), 4.73 (d, *J* = 2.4 Hz, 3H), 3.97 (s, 3H), 2.52 (t, *J* = 2.4 Hz, 1H). ^{13}C NMR (101 MHz, $CDCl_3$) δ (ppm): 155.6, 144.7, 131.0, 126.7, 121.9, 121.7, 112.5, 78.3, 76.0, 62.1, 56.5. Other spectroscopic data were previously reported in the literature[2]

2. Copies of NMR spectra

(*E*)-3-hydroxy-5-(hydroxymethyl)-2-methylisonicotinaldehyde *O*-benzyl oxime (2a)

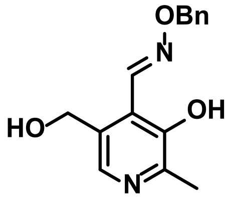

^{1}H NMR (400 MHz, CDCl3)

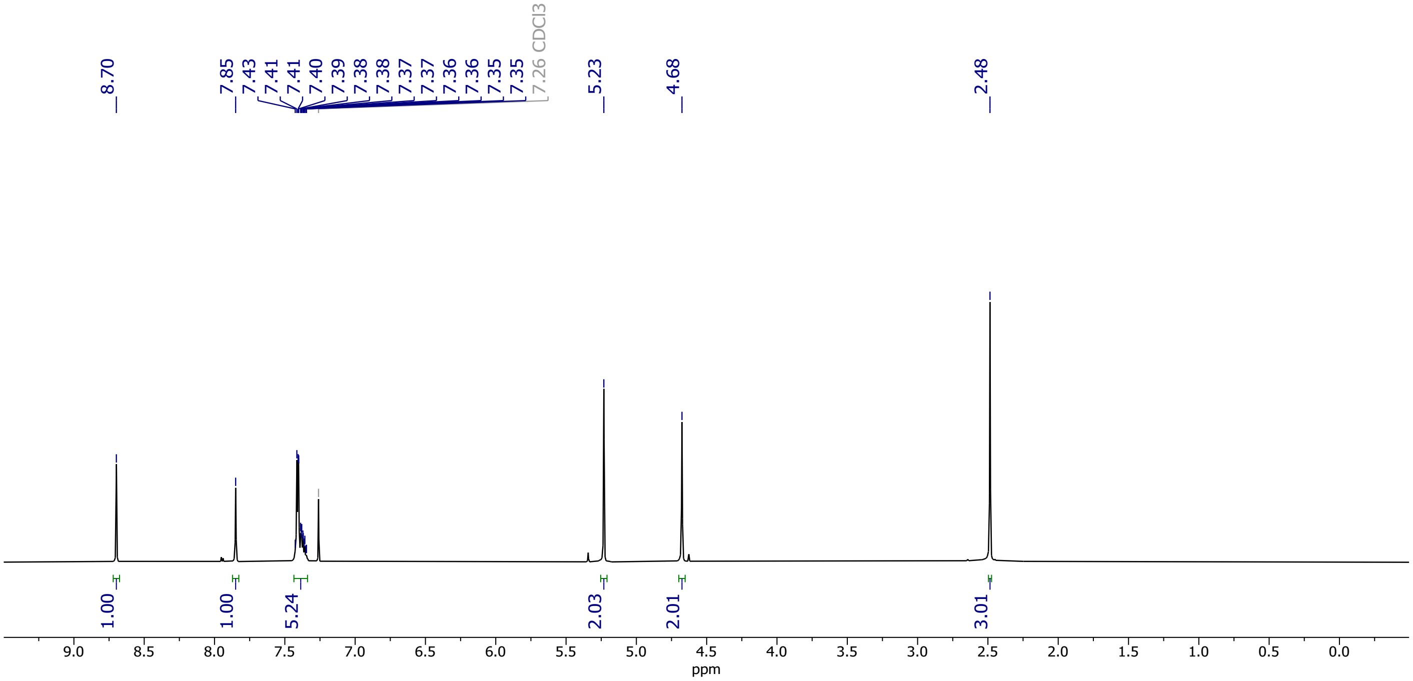

^{13}C NMR (101 MHz, CDCl3)

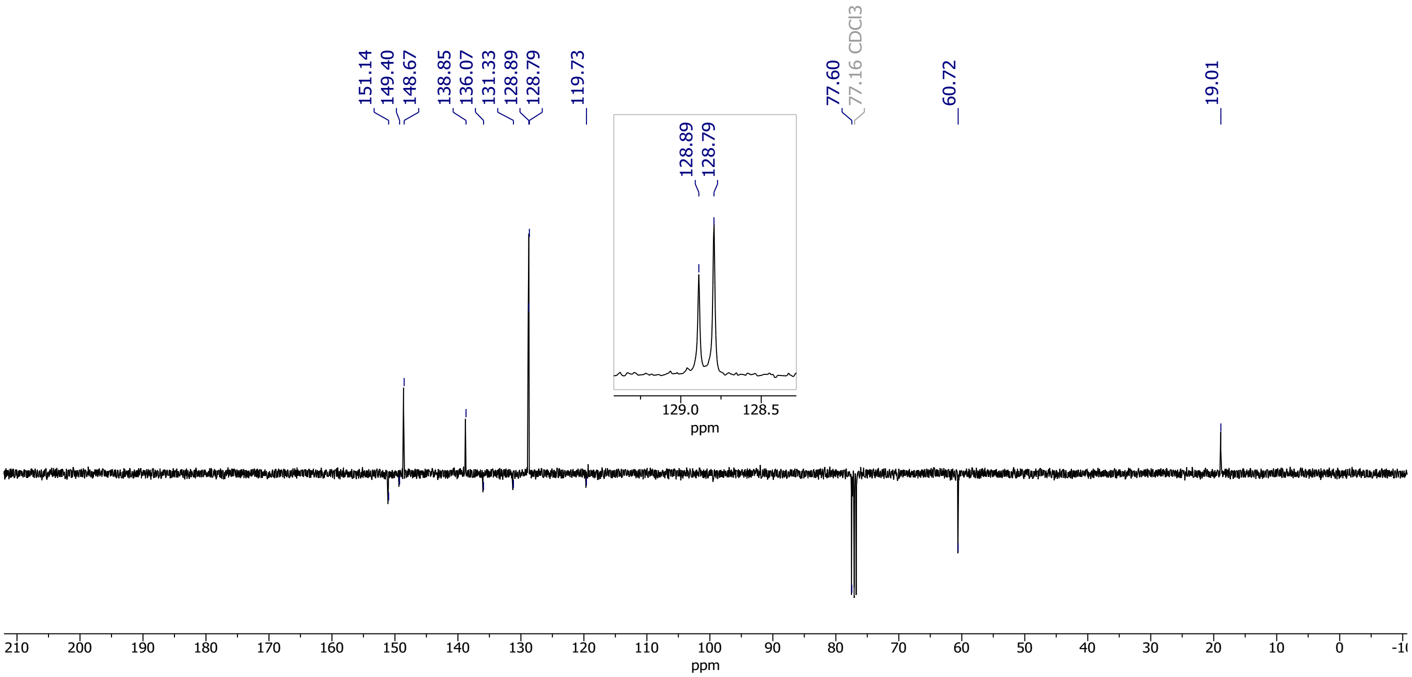

(*E*)-3-hydroxy-5-(hydroxymethyl)-2-methylisonicotinaldehyde *O*-methyl oxime (2b)

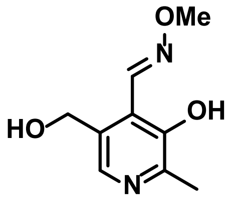

^{1}H NMR (300 MHz, CDCl$_3$)

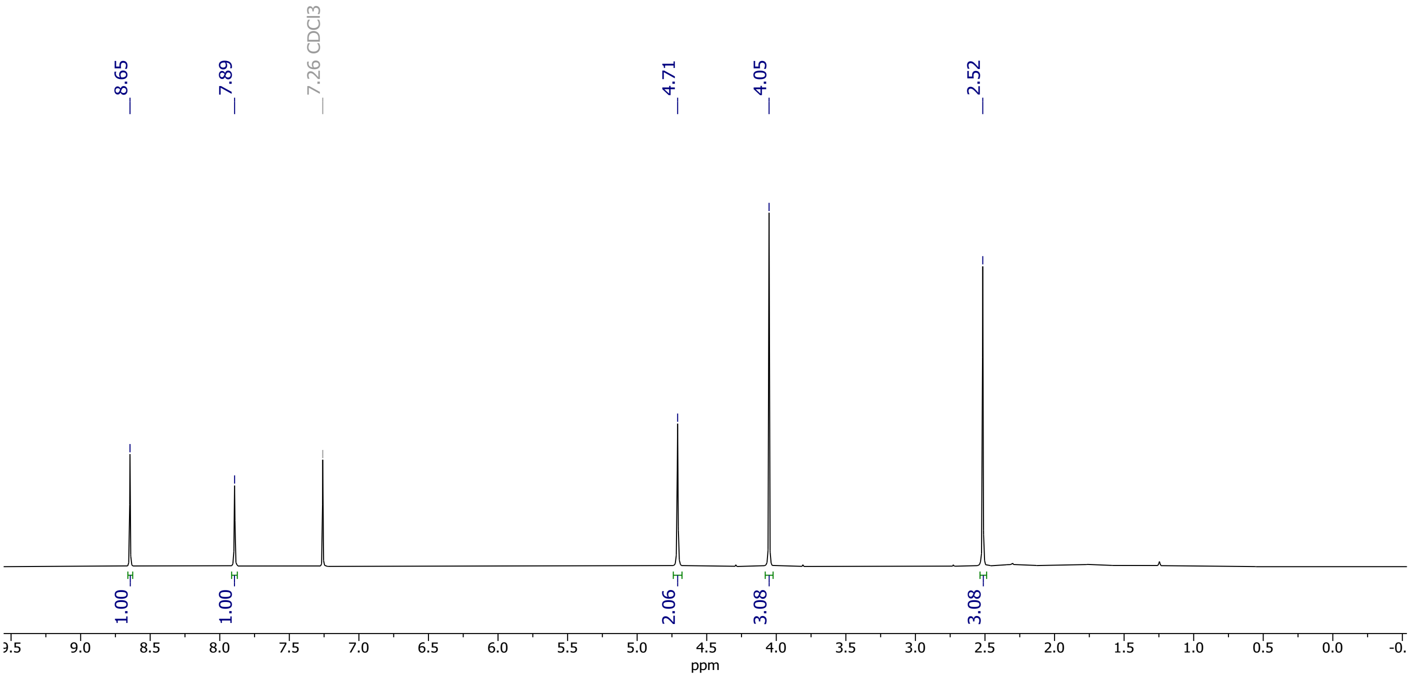

^{13}C NMR (75 MHz, CDCl$_3$)

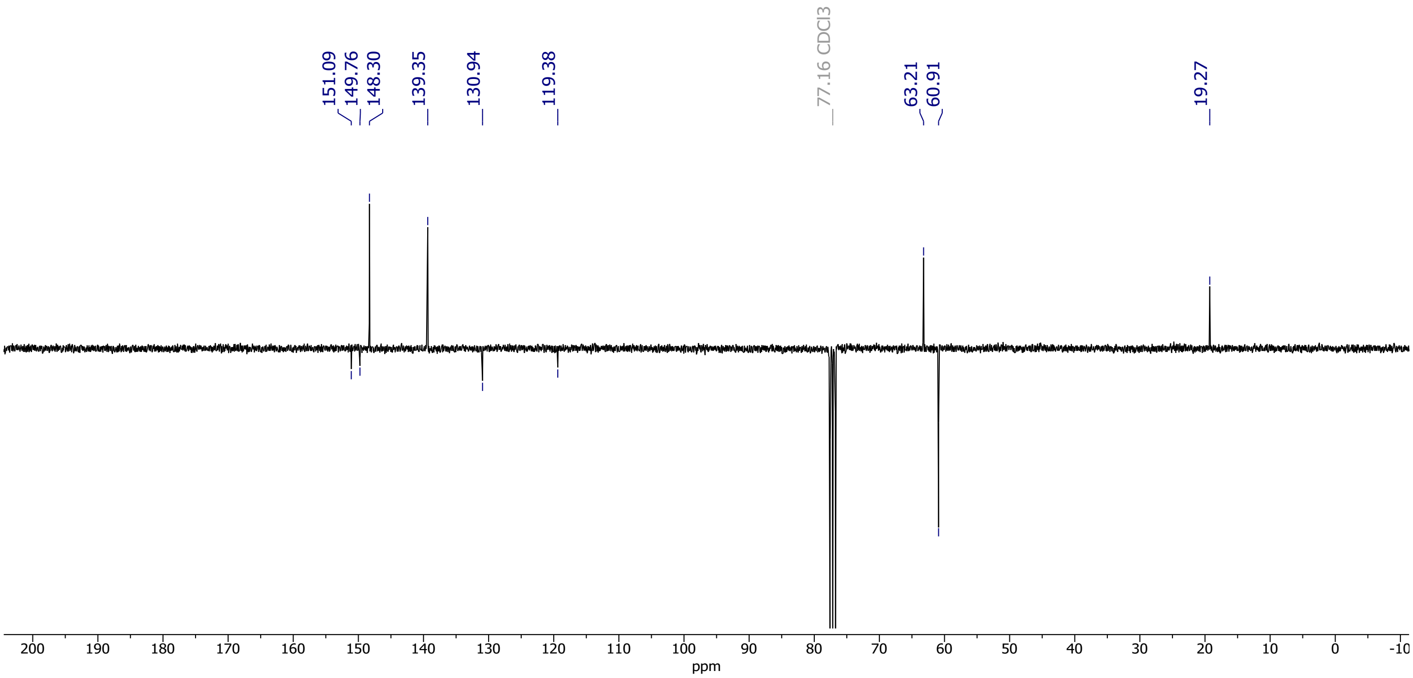

(*E*)-3-((2-bromobenzyl)oxy)-5-(hydroxymethyl)-2-methylisonicotinaldehyde *O*-benzyl oxime (3a)

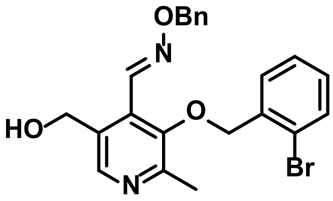

^{1}H NMR (400 MHz, CDCl3)

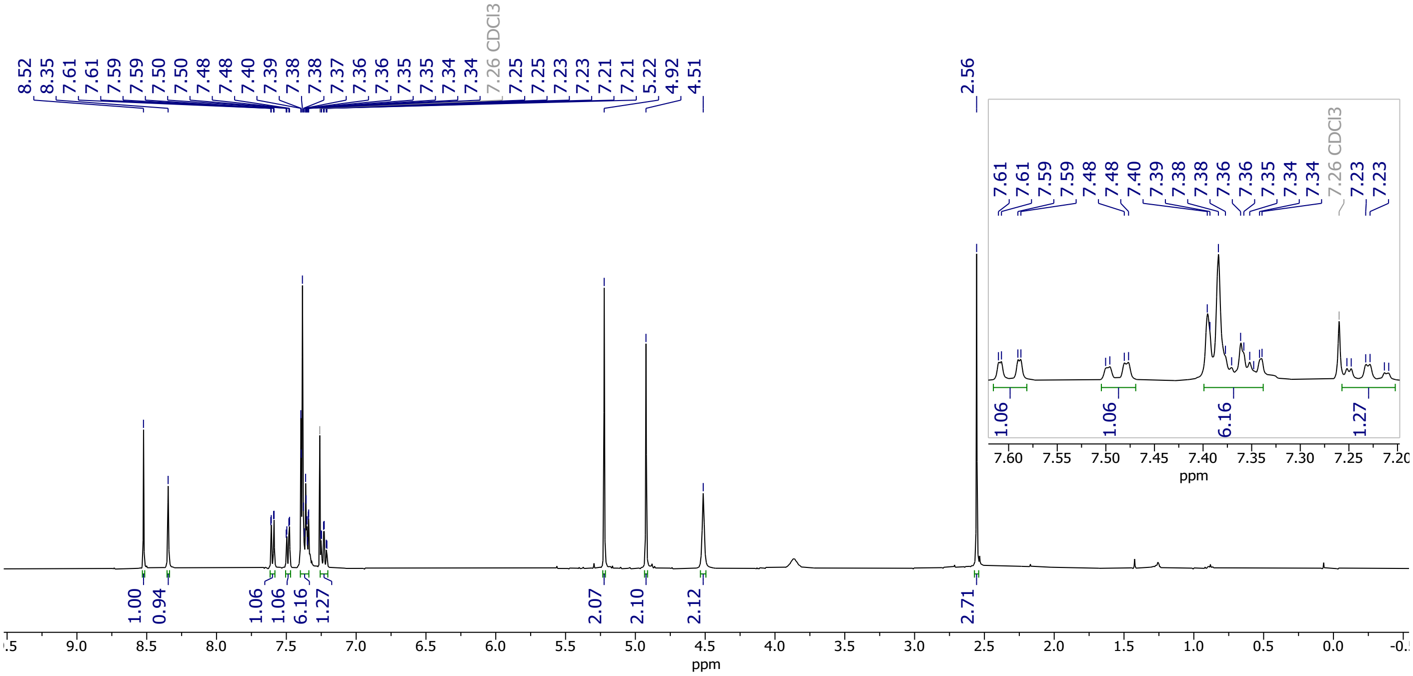

^{13}C NMR (101 MHz, CDCl3)

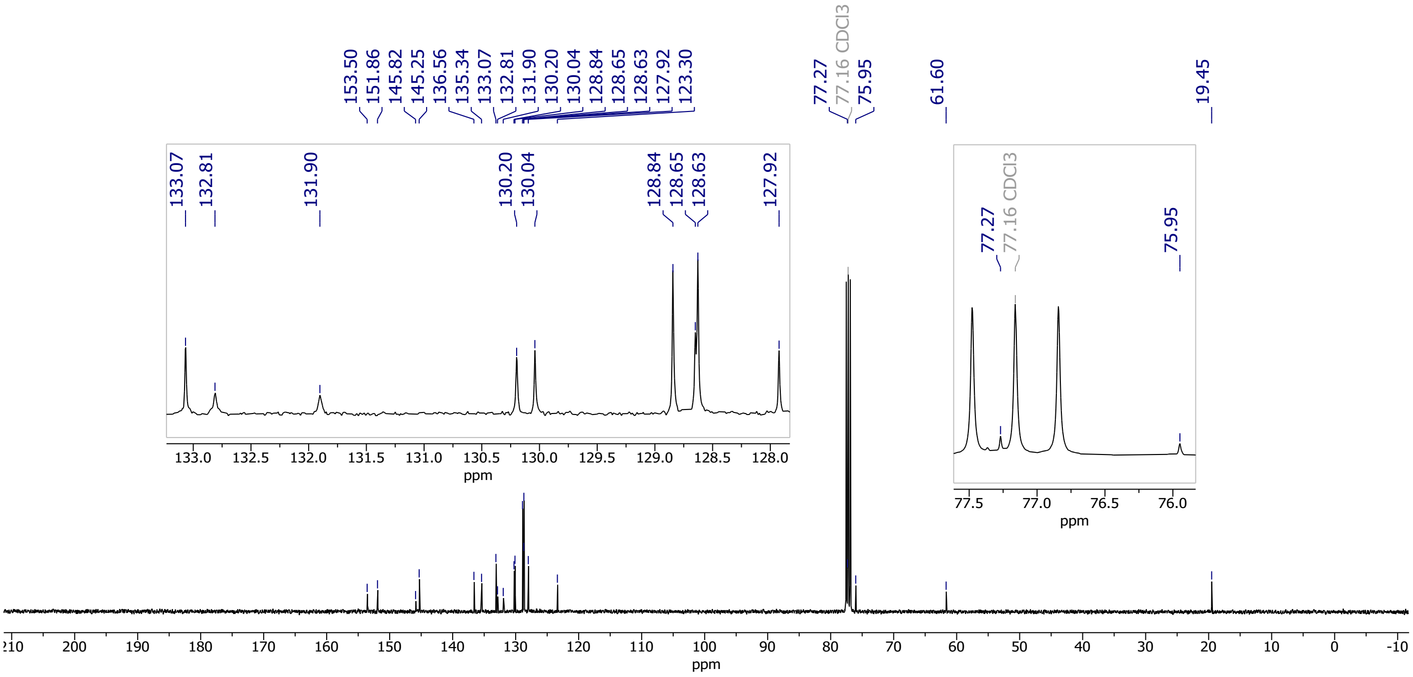

(*E*)-3-((2-bromobenzyl)oxy)-5-(hydroxymethyl)-2-methylisonicotinaldehyde *O*-methyl oxime (3b)

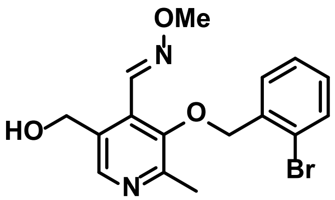

^{1}H NMR (400 MHz, CDCl$_3$)

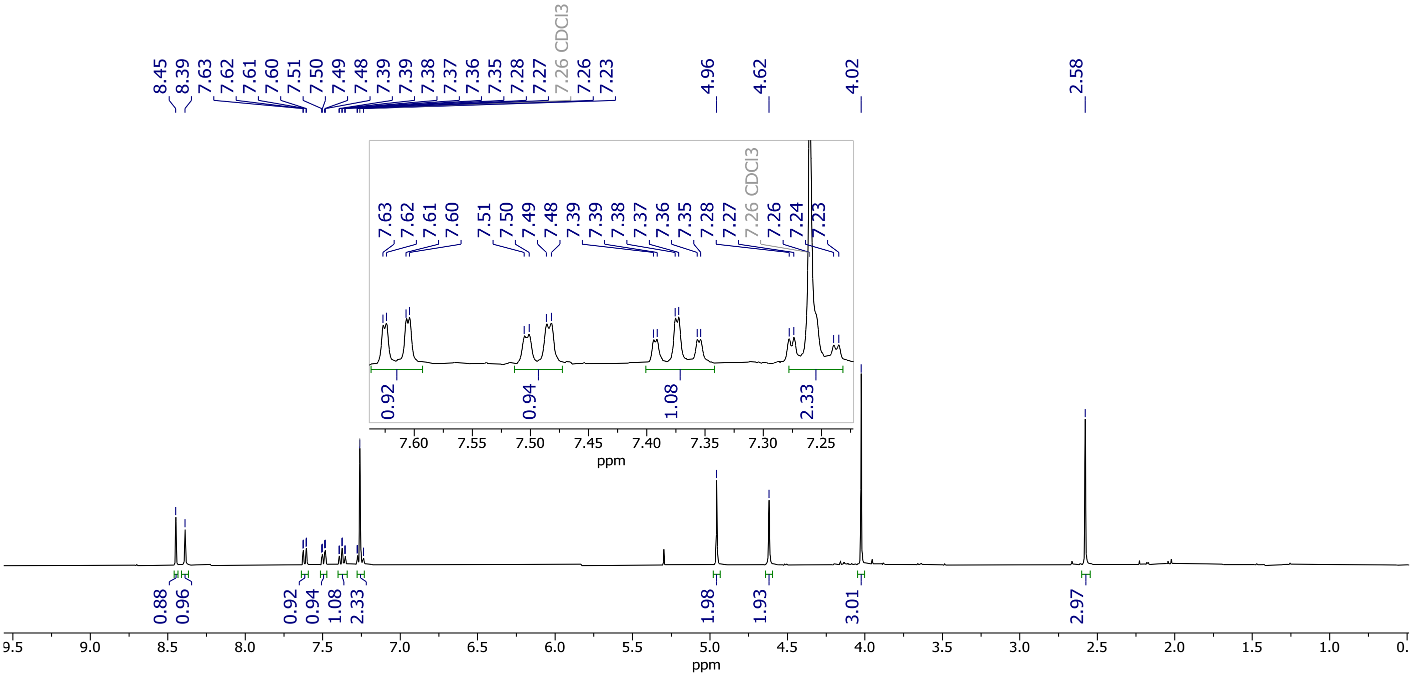

^{13}C NMR (101 MHz, CDCl$_3$)

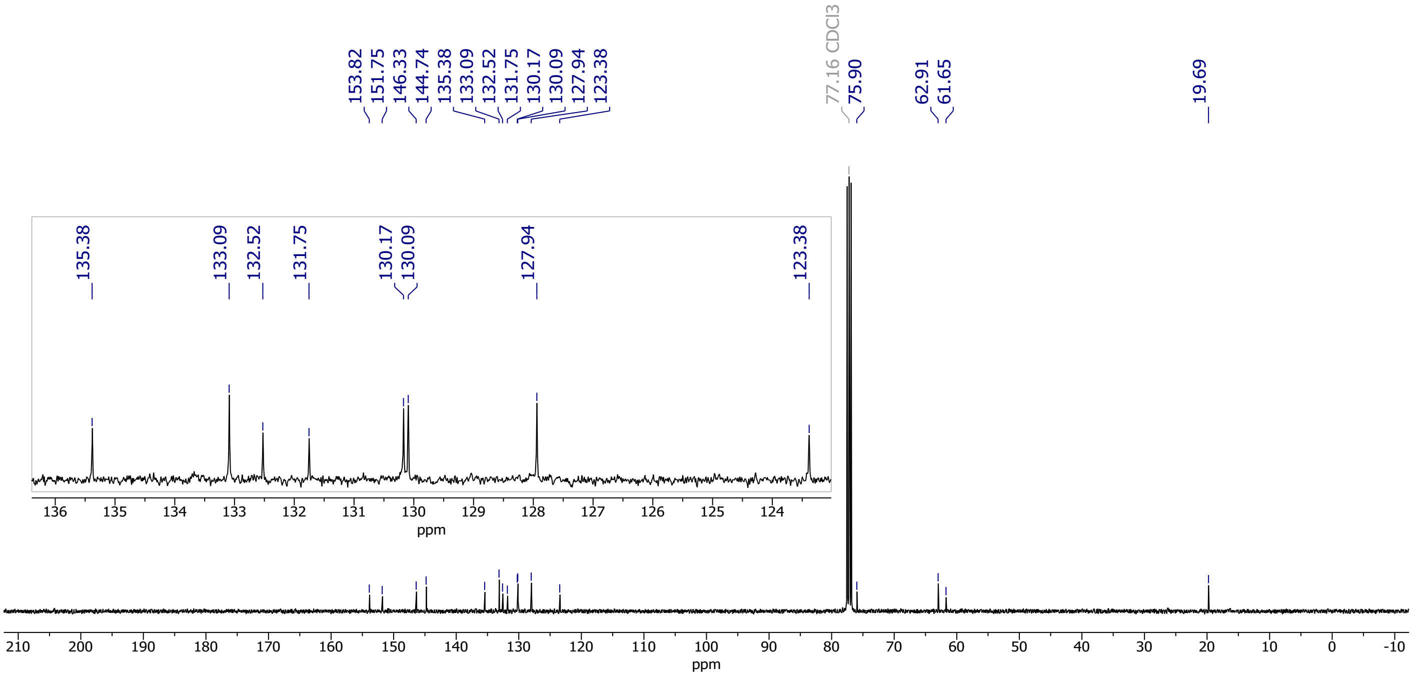

(*E*)-5-(hydroxymethyl)-2-methyl-3-(prop-2-yn-1-yloxy)isonicotinaldehyde *O*-benzyl oxime (3c)

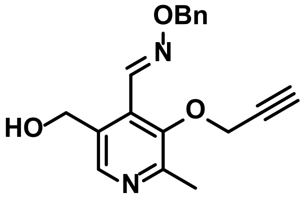

^{1}H NMR (300 MHz, CDCl$_3$)

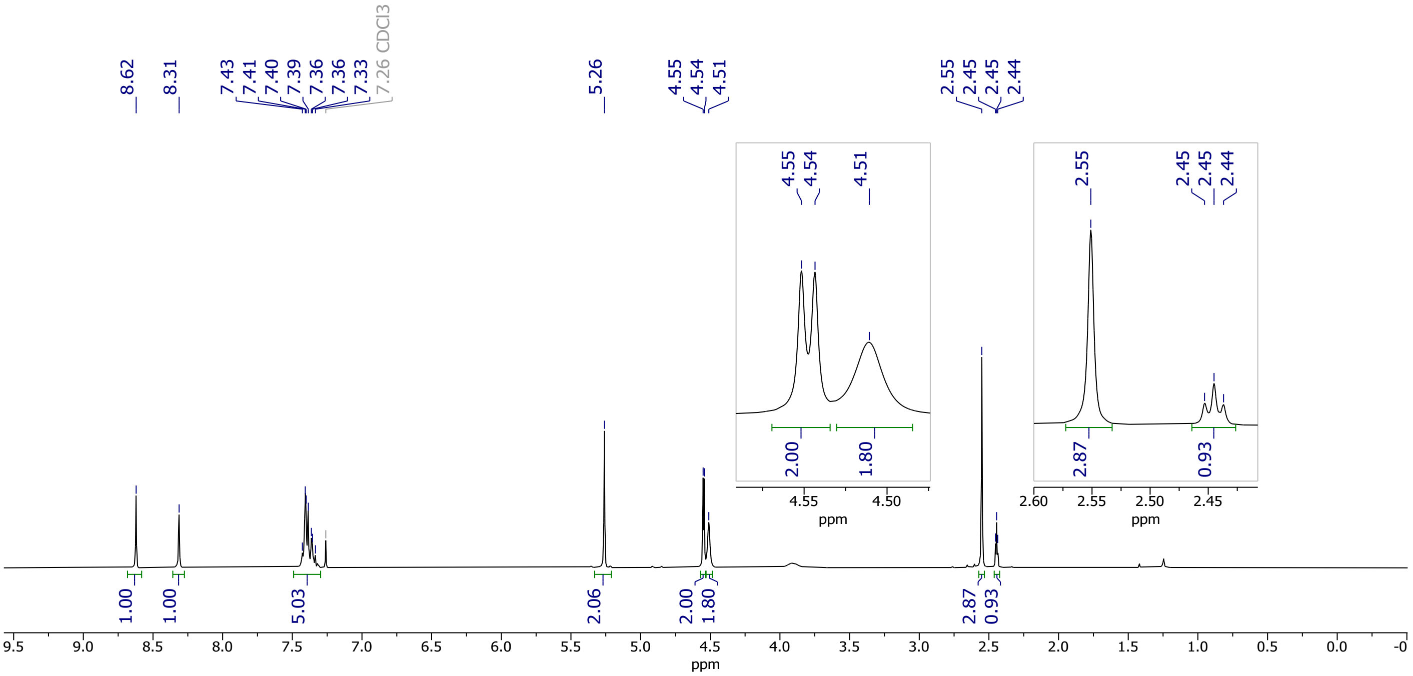

^{13}C NMR (75 MHz, CDCl$_3$)

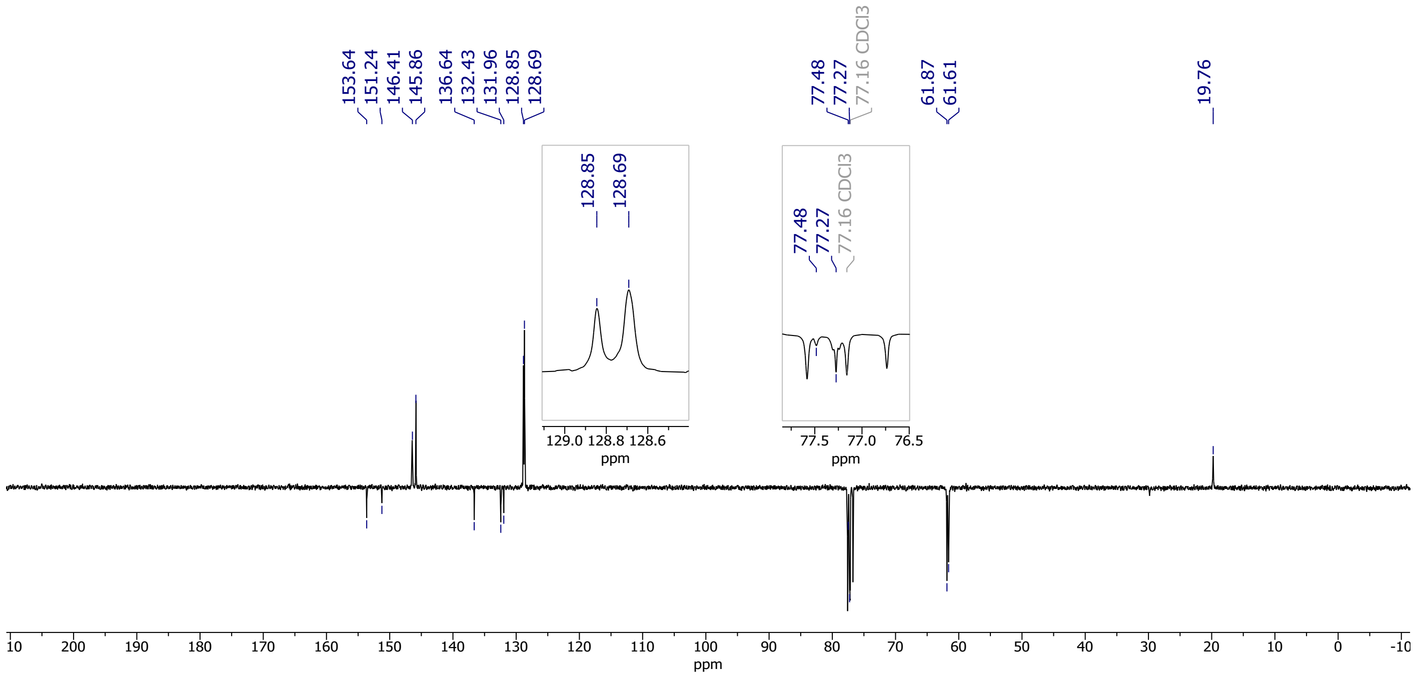

(*E*)-5-(hydroxymethyl)-2-methyl-3-(prop-2-yn-1-yloxy)isonicotinaldehyde *O*-methyl oxime (3d)

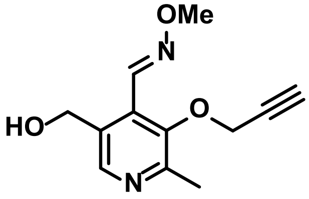

^{1}H NMR (300 MHz, CDCl$_3$)

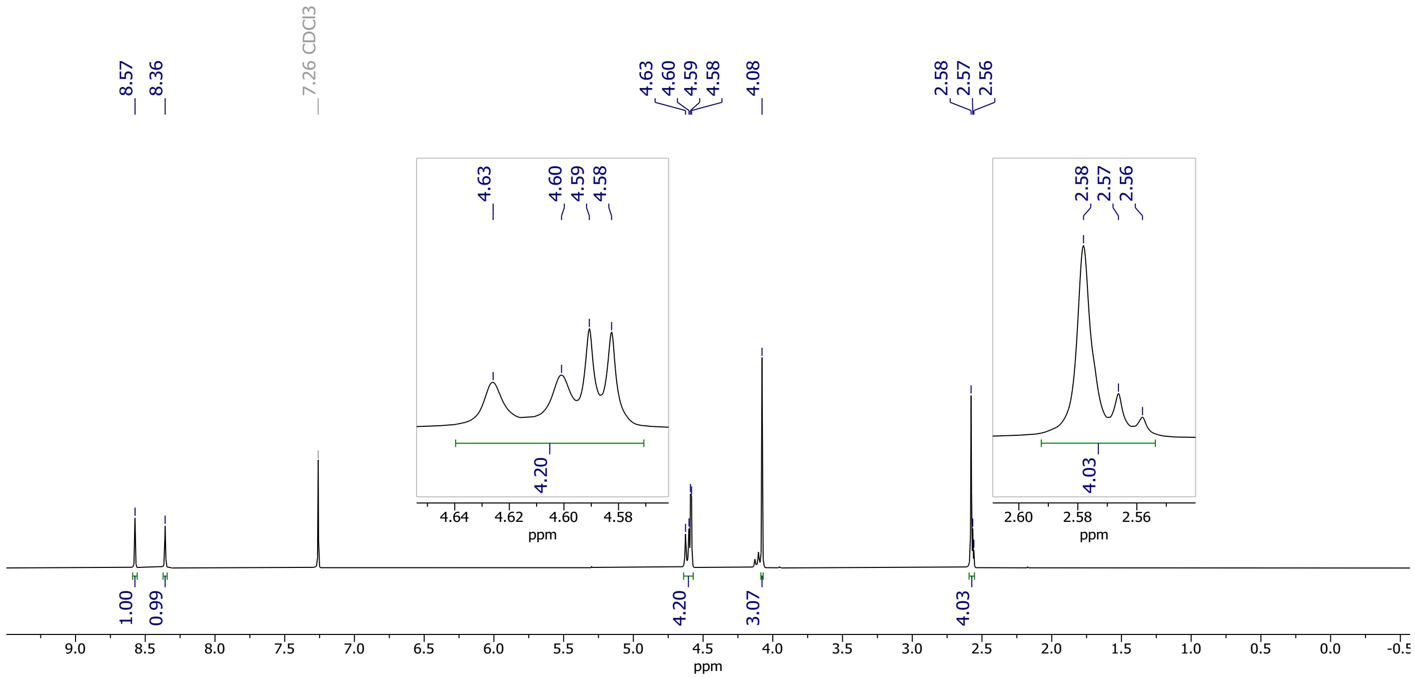

^{13}C NMR (75 MHz, CDCl$_3$)

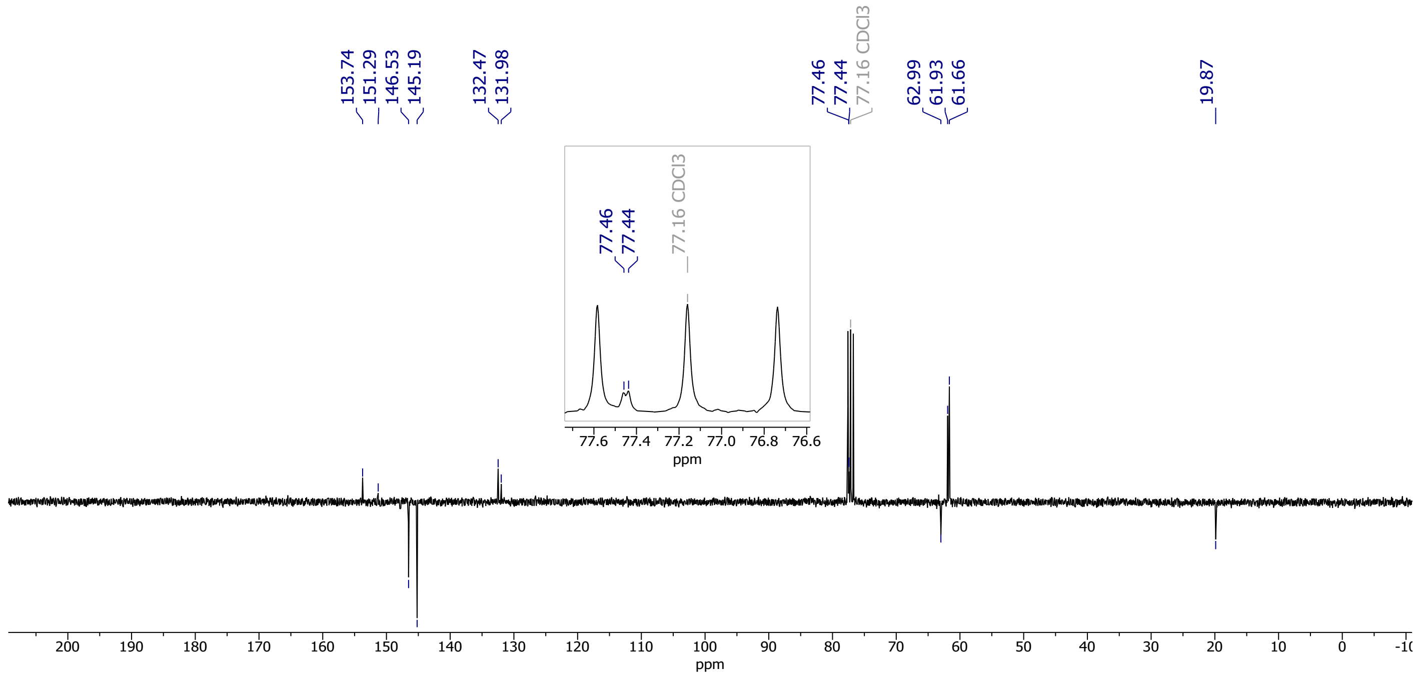

(*E*)-4-(((benzyloxy)imino)methyl)-5-((2-bromobenzyl)oxy)-6-methylnicotinaldehyde (4a)

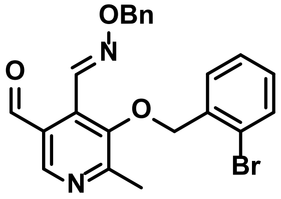

^{1}H NMR (400 MHz, CDCl₃)

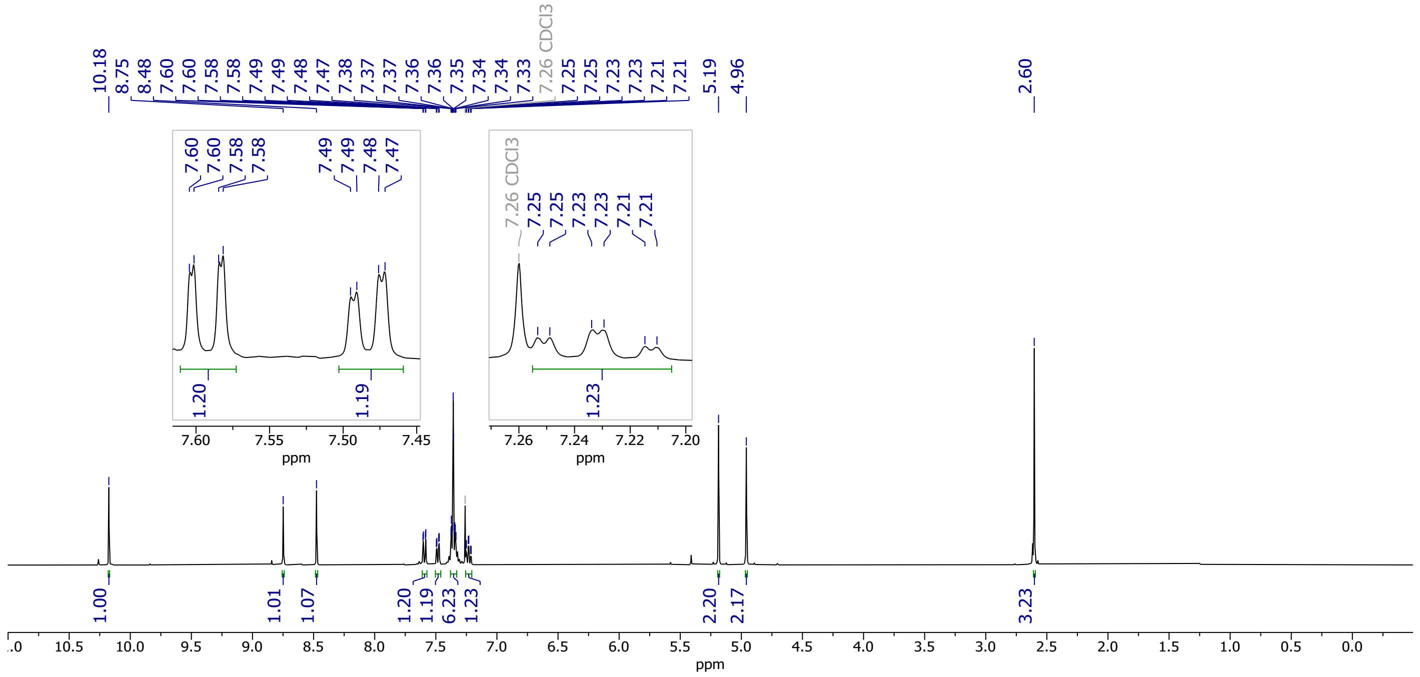

^{13}C NMR (101 MHz, CDCl₃)

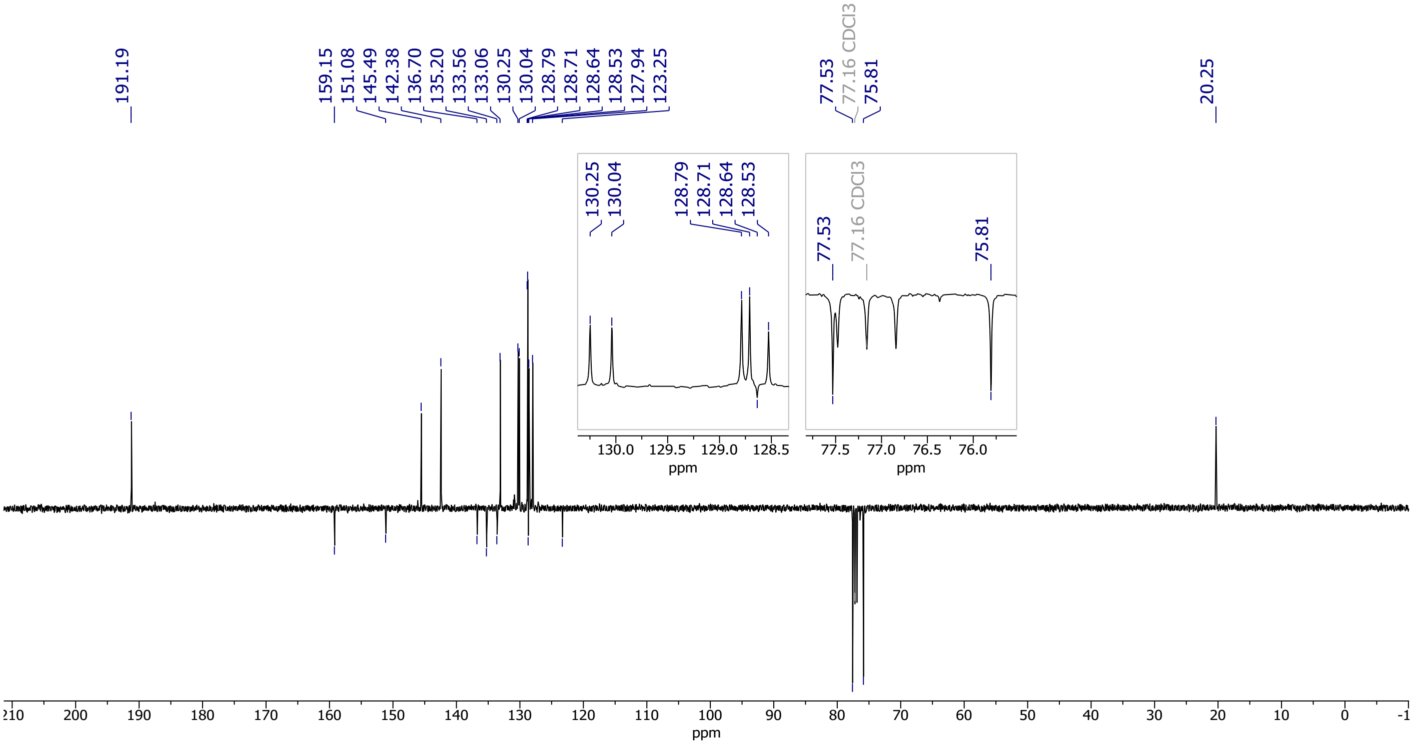

(*E*)-5-((2-bromobenzyl)oxy)-4-((methoxyimino)methyl)-6-methylnicotinaldehyde (4b)

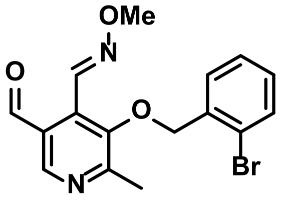

^{1}H NMR (300 MHz, $CDCl_3$)

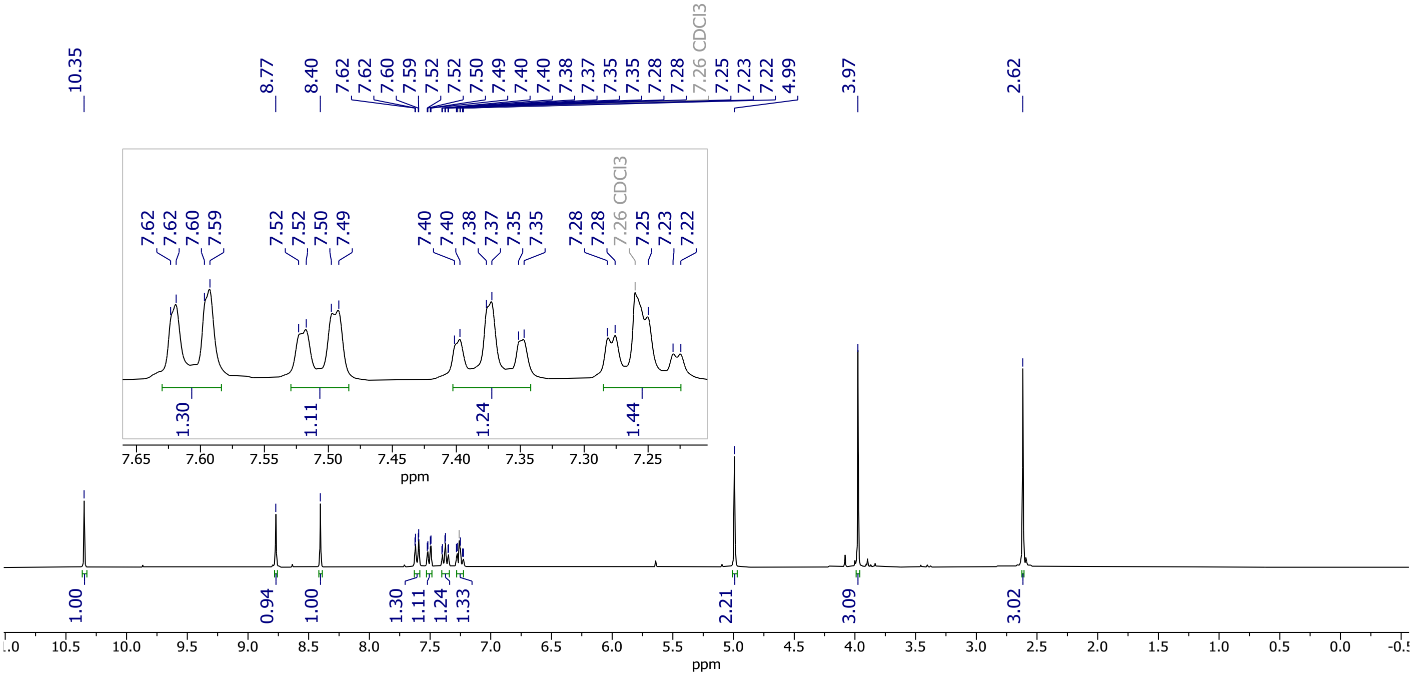

^{13}C NMR (75 MHz, $CDCl_3$)

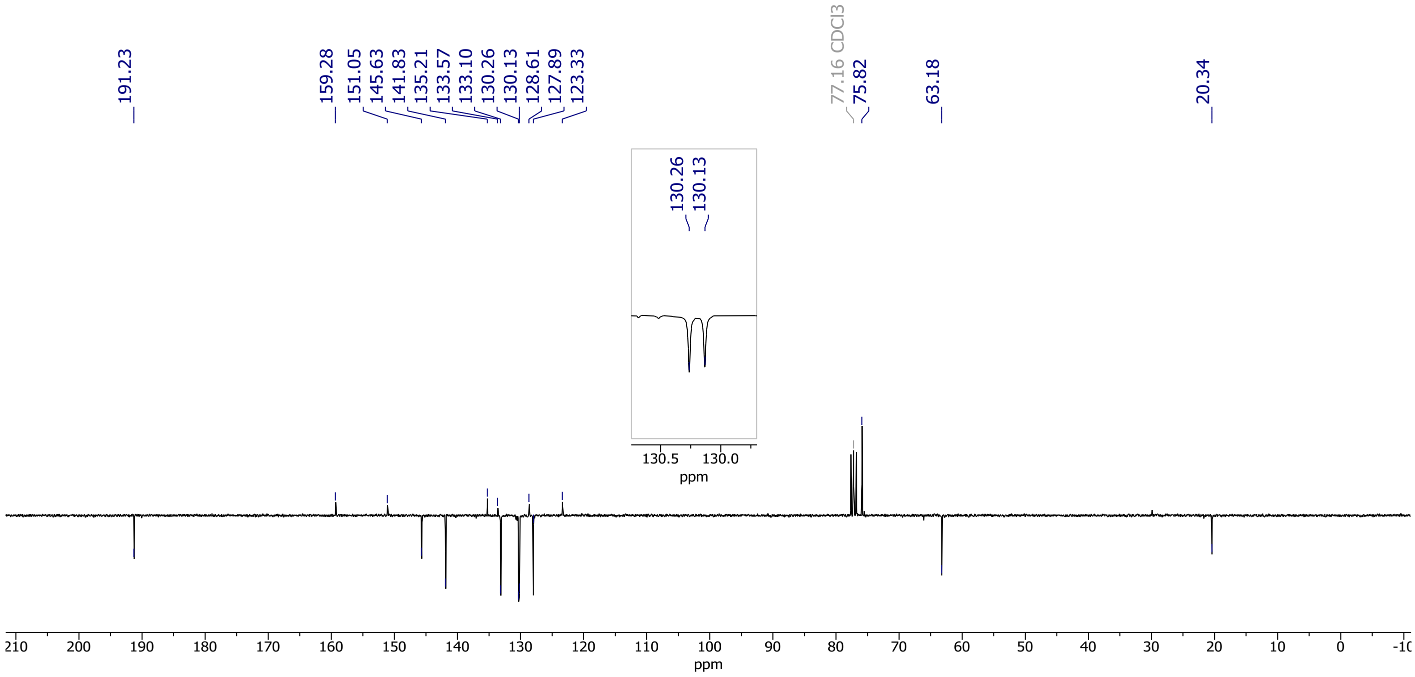

(*E*)-4-(((benzyloxy)imino)methyl)-6-methyl-5-(prop-2-yn-1-yloxy)nicotinaldehyde (4c)

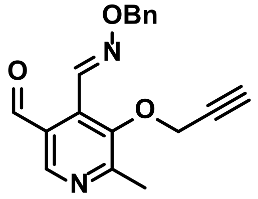

^{1}H NMR (400 MHz, CDCl$_3$)

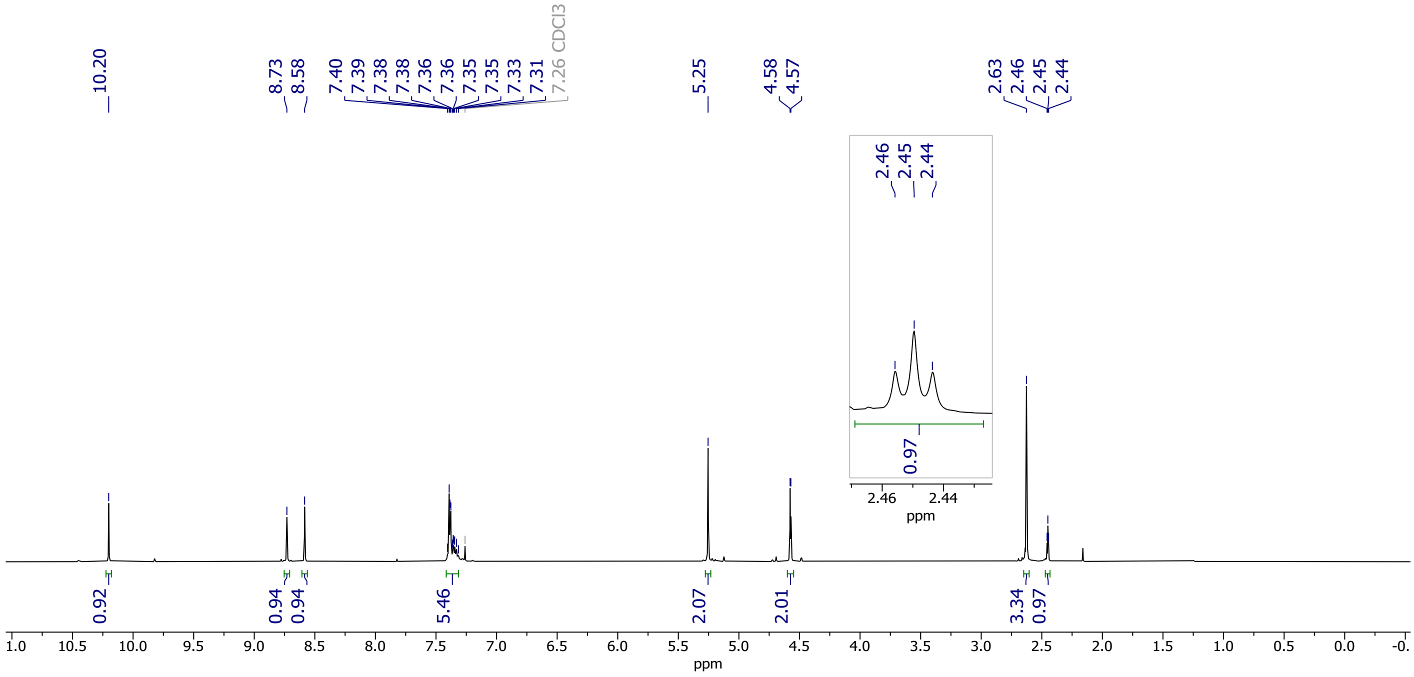

^{13}C NMR (101 MHz, CDCl$_3$)

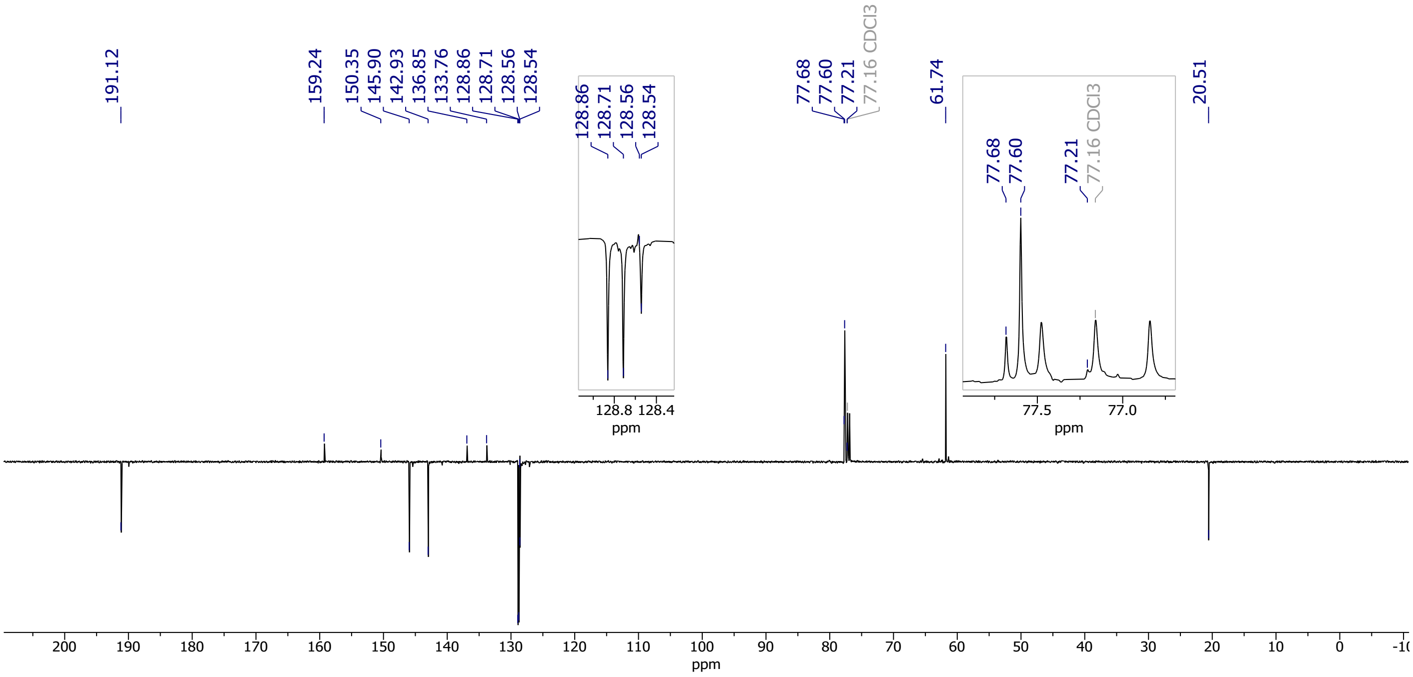

(*E*)-4-((methoxyimino)methyl)-6-methyl-5-(prop-2-yn-1-yloxy)nicotinaldehyde (4d)

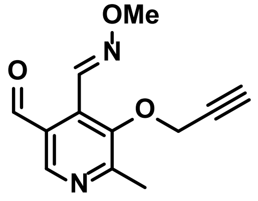

^{1}H NMR (300 MHz, CDCl$_3$)

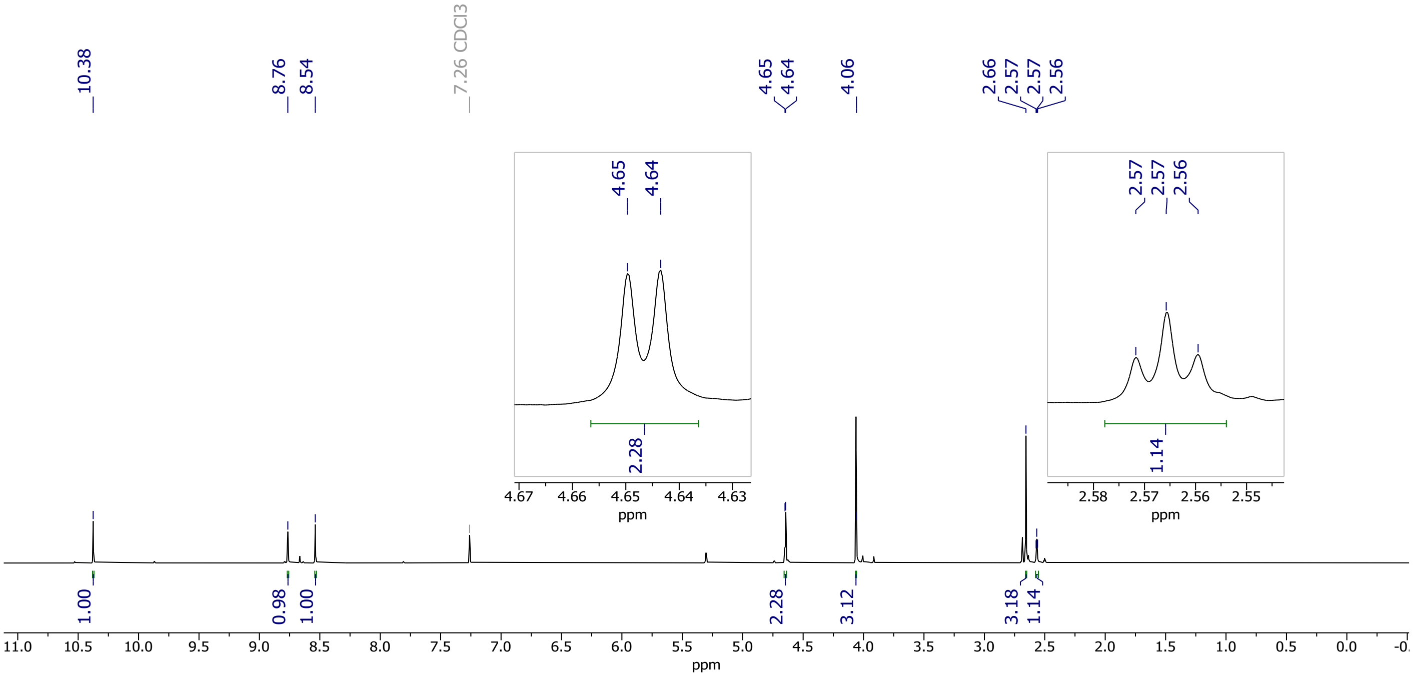

^{13}C NMR (75 MHz, CDCl$_3$)

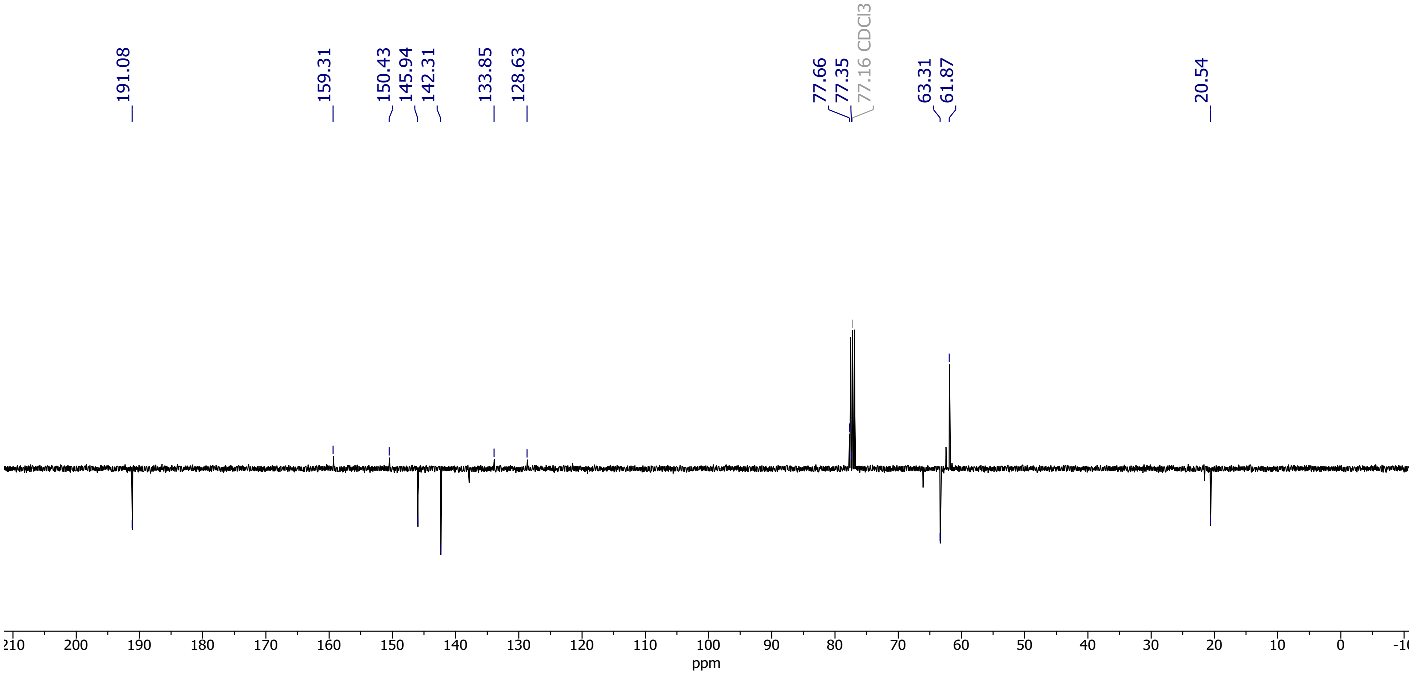

(*E*)-3-((2-bromobenzyl)oxy)-2-methyl-5-(*E*)-styryl)isonicotinaldehyde *O*-benzyl oxime (5a)

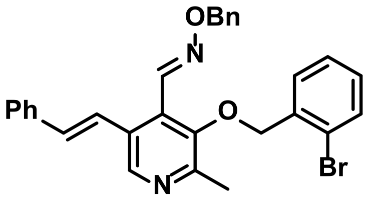

^{1}H NMR (400 MHz, CDCl$_3$)

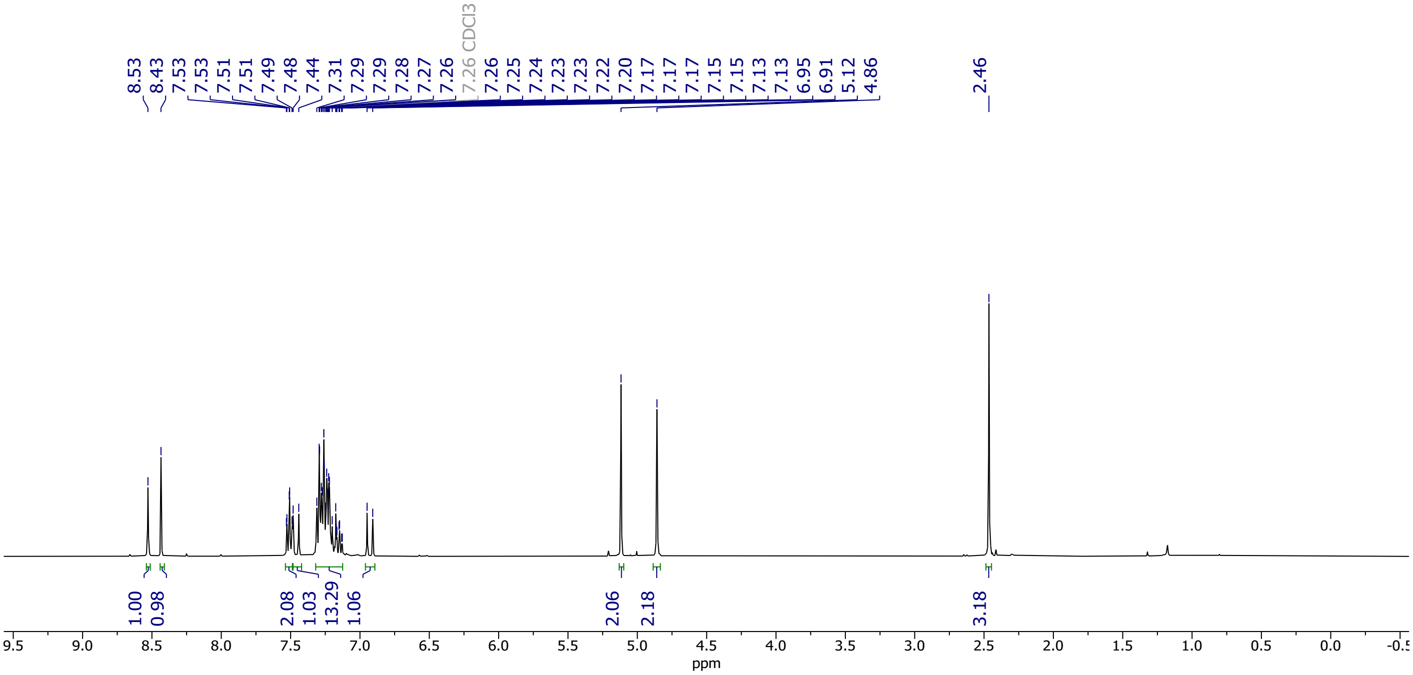

^{13}C NMR (101 MHz, CDCl$_3$)

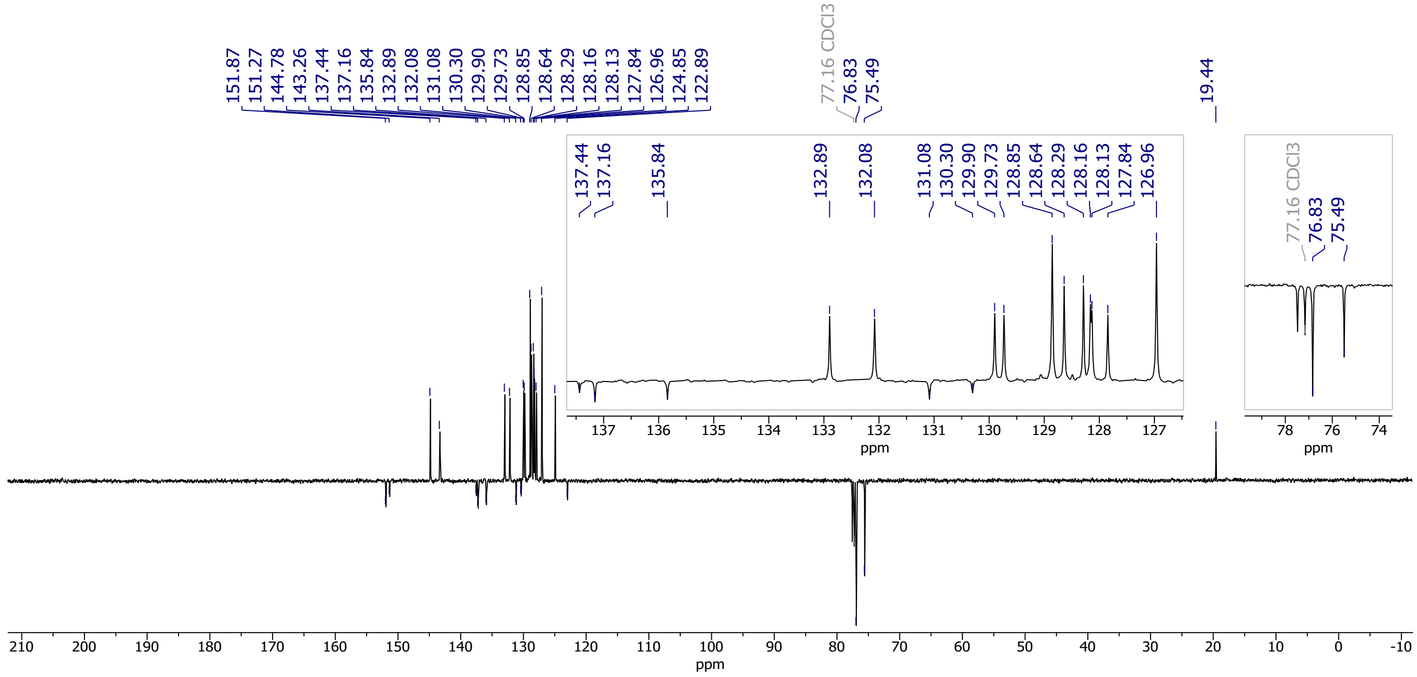

(*E*)-3-((2-bromobenzyl)oxy)-2-methyl-5-(*E*)-styryl)isonicotinaldehyde *O*-methyl oxime (5b)

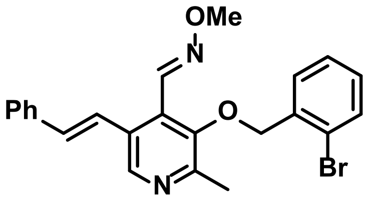

^{1}H NMR (300 MHz, CDCl$_3$)

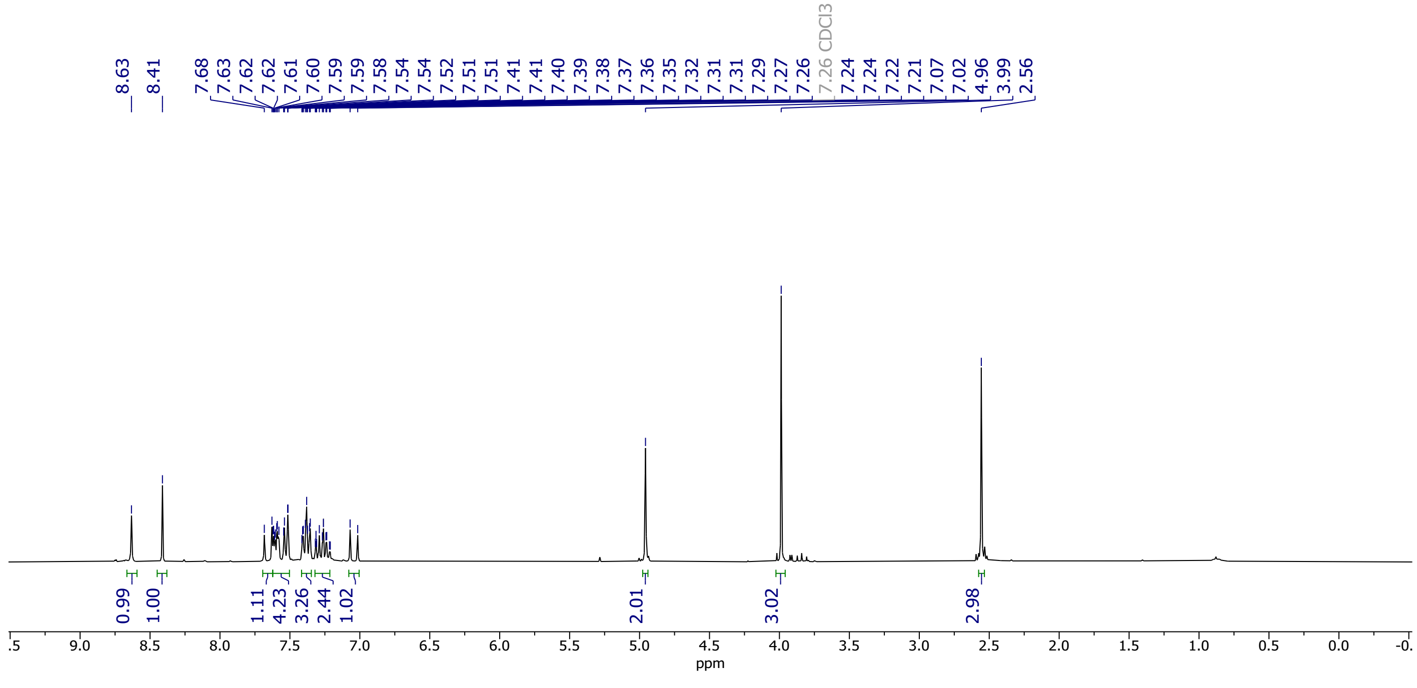

^{13}C NMR (75 MHz, $CDCl_3$)

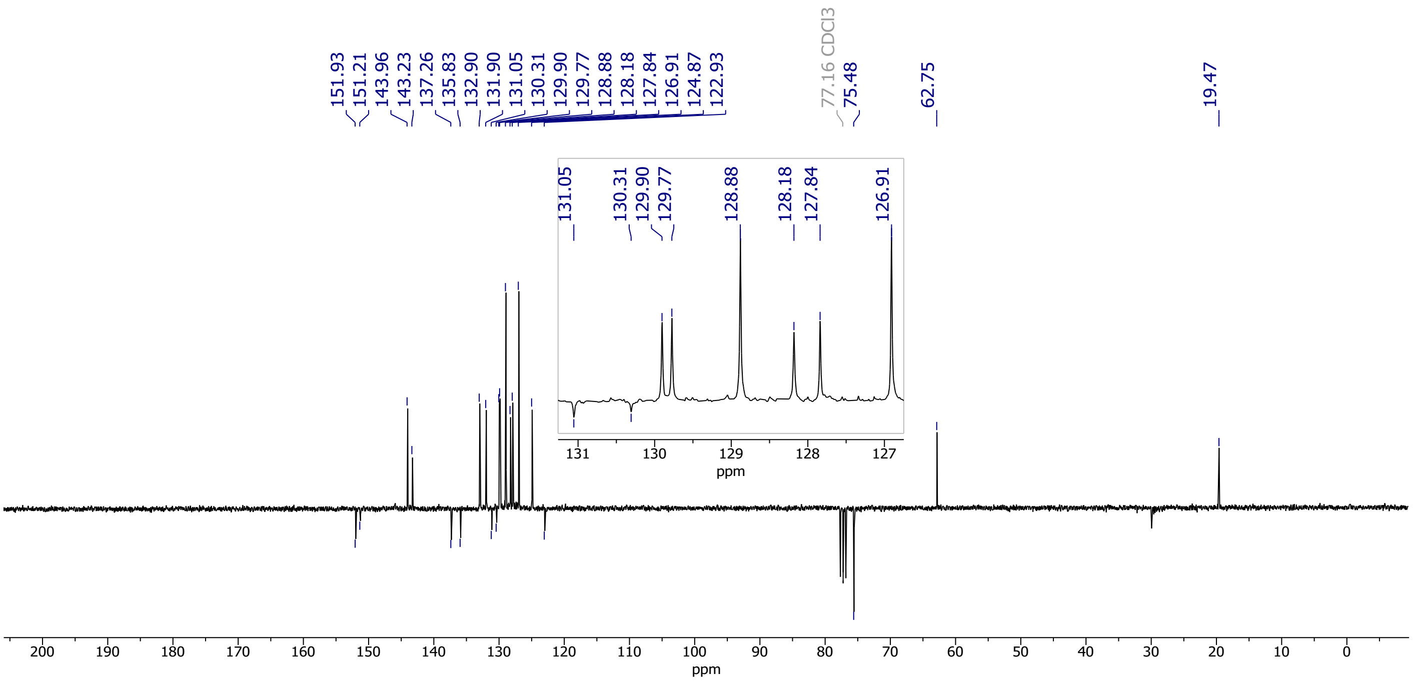

(*E*)-2-methyl-3-(prop-2-yn-1-yloxy)-5-styrylisonicotinaldehyde *O*-benzyl oxime (5c)

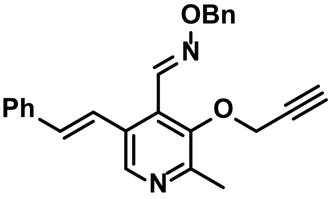

^{1}H NMR (300 MHz, $CDCl_3$)

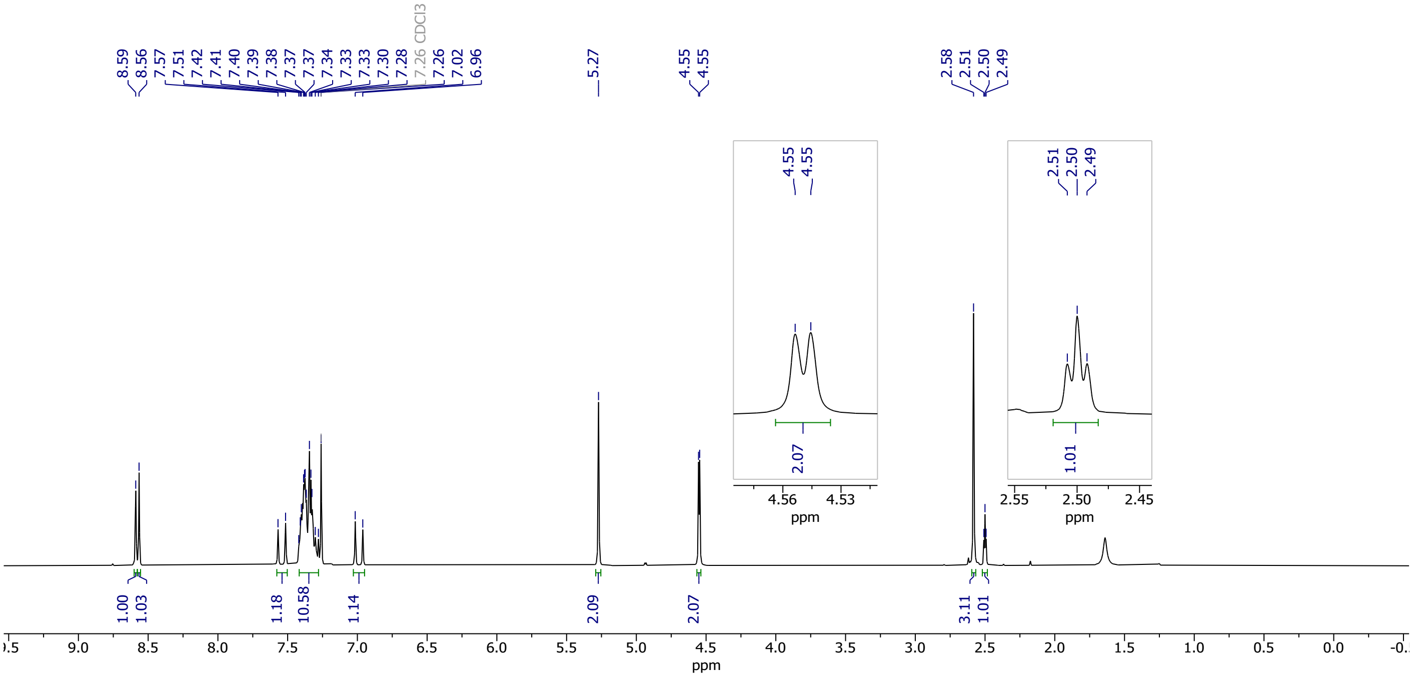

^{13}C NMR (75 MHz, $CDCl_3$)

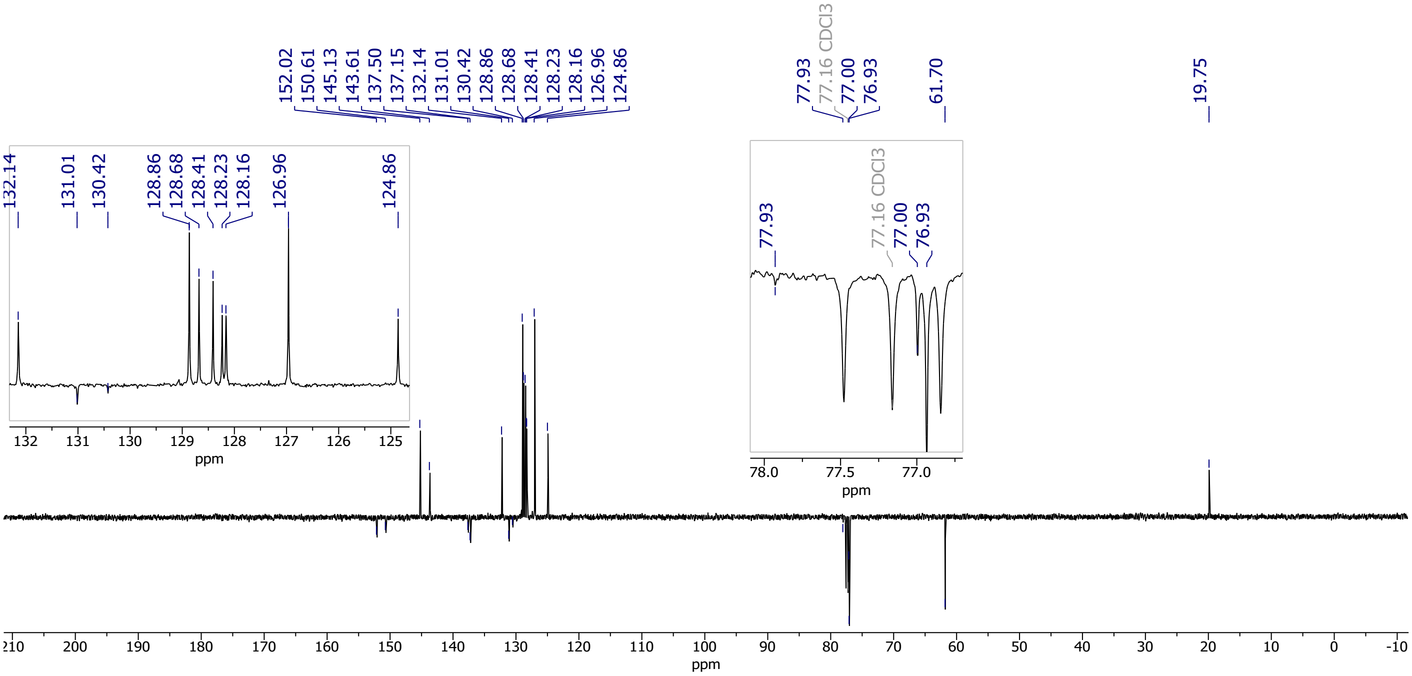

(5-((benzyloxy)amino)-1-methyl-5,10-dihydrobenzo[5,6]oxepino[2,3-c]pyridin-4-yl)methanol (6a)

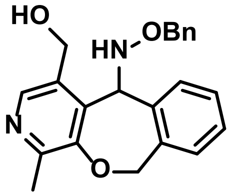

^{1}H NMR (300 MHz, $CDCl_3$)

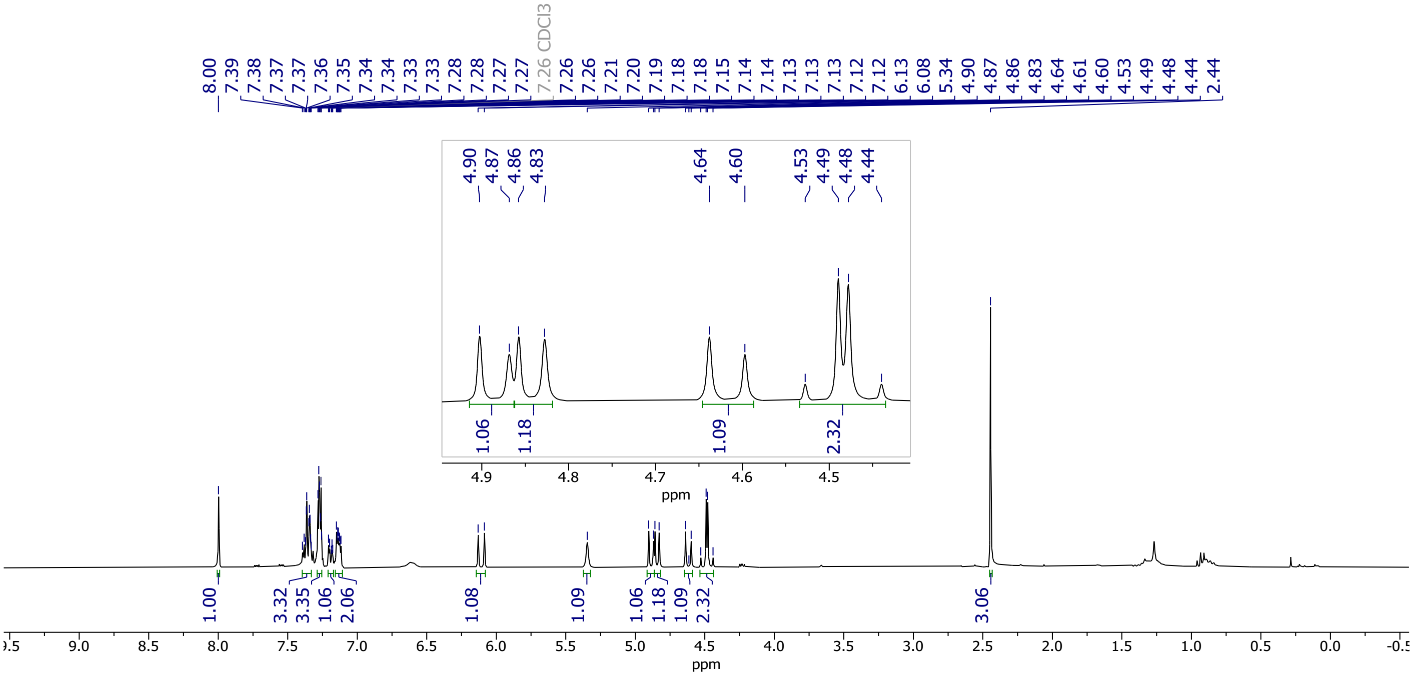

^{13}C NMR (75 MHz, $CDCl_3$)

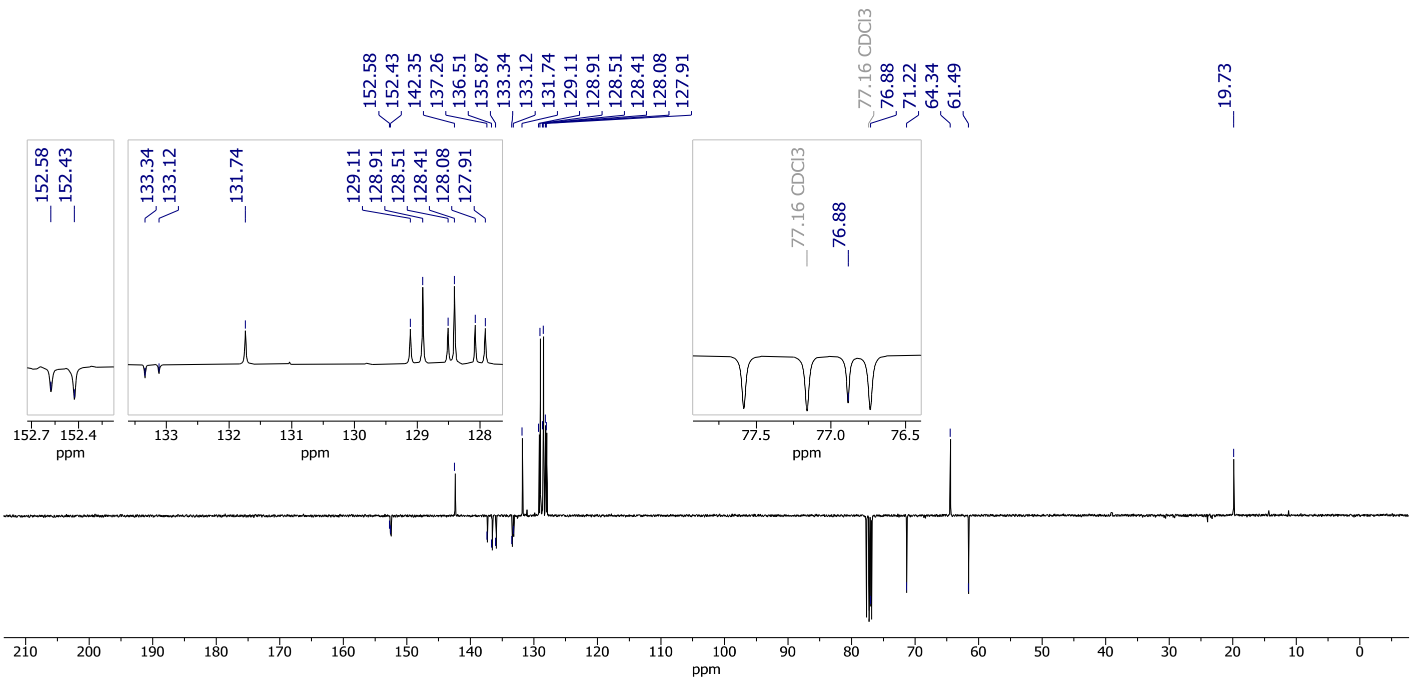

(5-(methoxyamino)-1-methyl-5,10-dihydrobenzo[5,6]oxepino[2,3-c]pyridin-4-yl)methanol (6b)

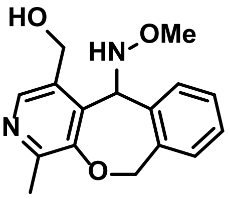

^{1}H NMR (300 MHz, $CDCl_3$)

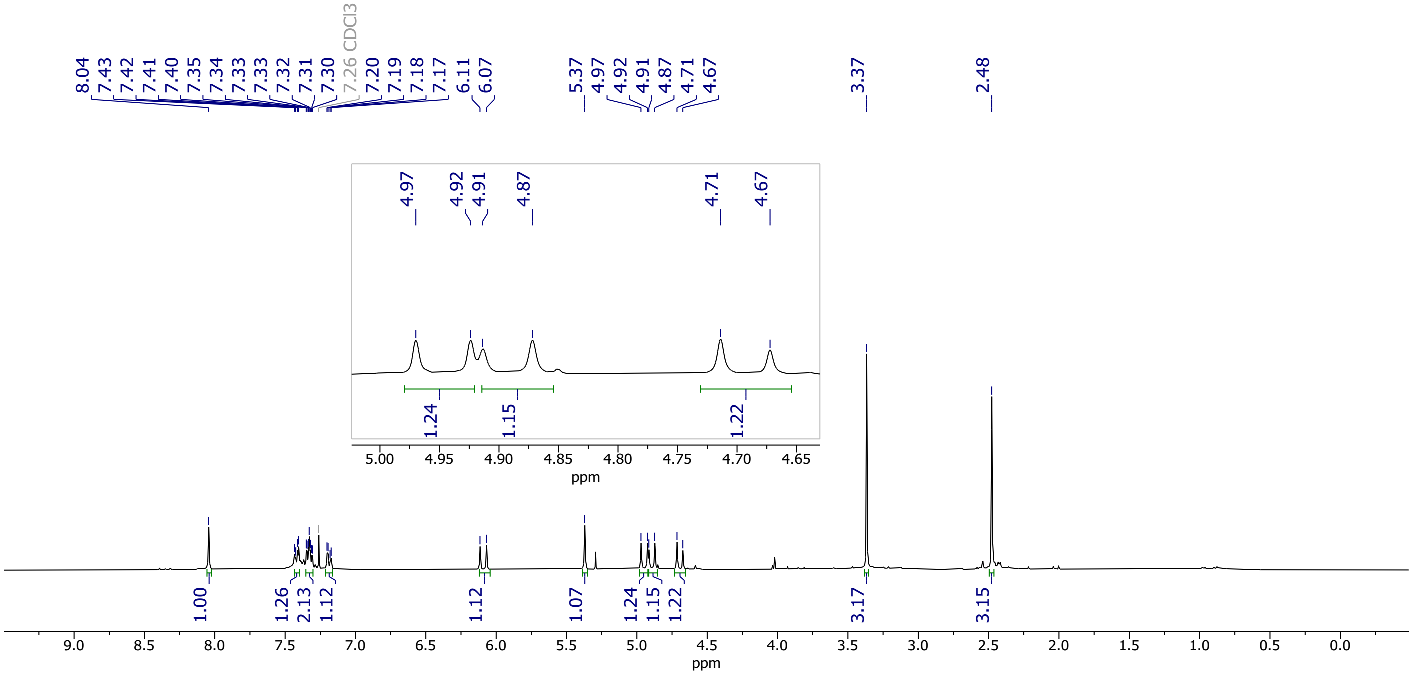

^{13}C NMR (75 MHz, CDCl$_3$)

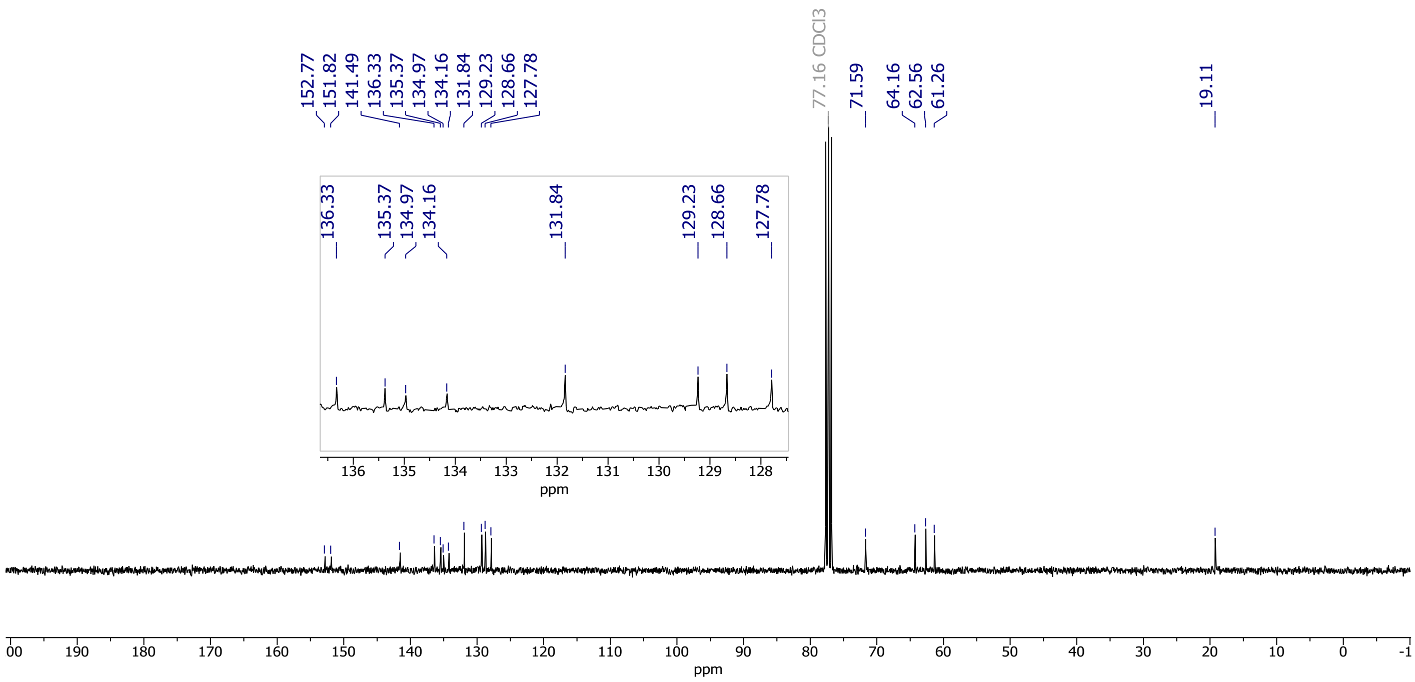

***(E)*-3-((1-(2-bromobenzyl)-1*H*-1,2,3-triazol-4-yl)methoxy)-5-(hydroxymethyl)-2-methylisonicotinaldehyde *O*-benzyl oxime (7a)**

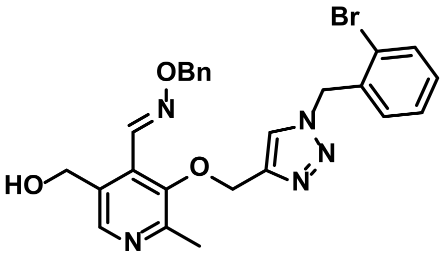

^{1}H NMR (300 MHz, CDCl$_3$)

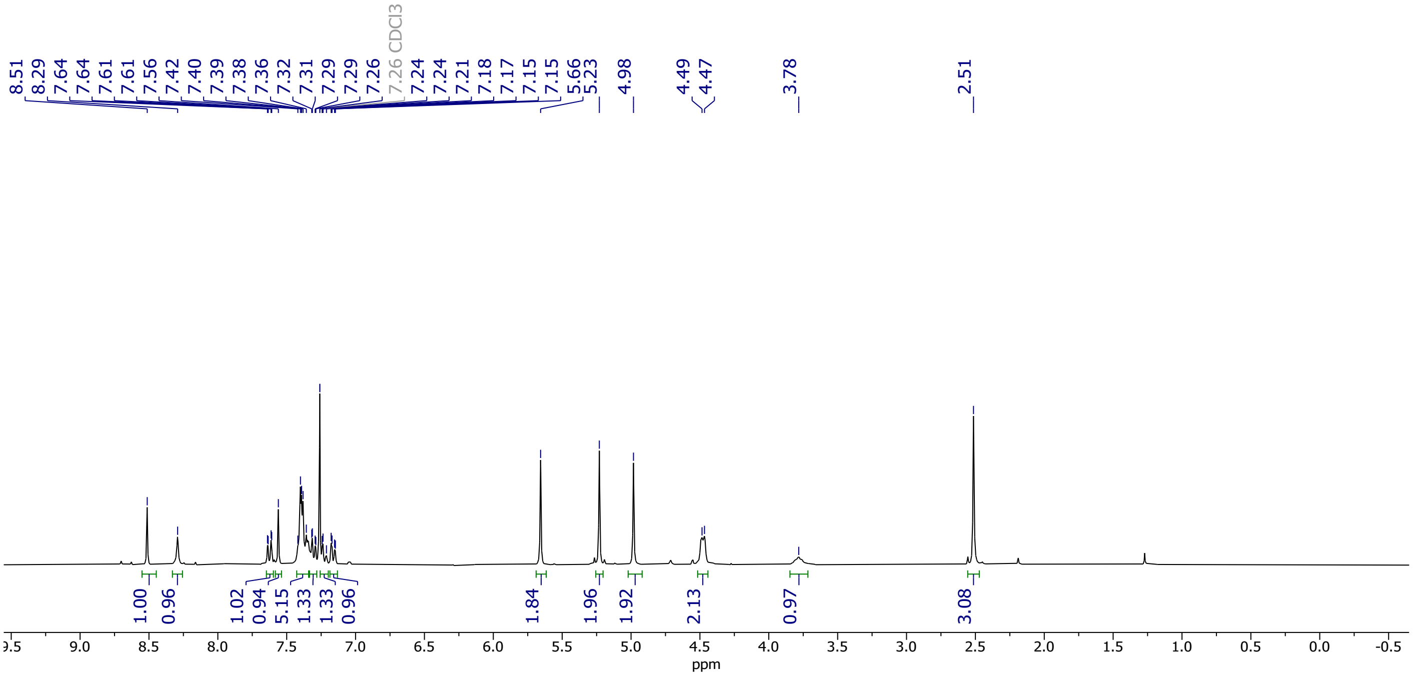

^{13}C NMR (75 MHz, CDCl$_3$)

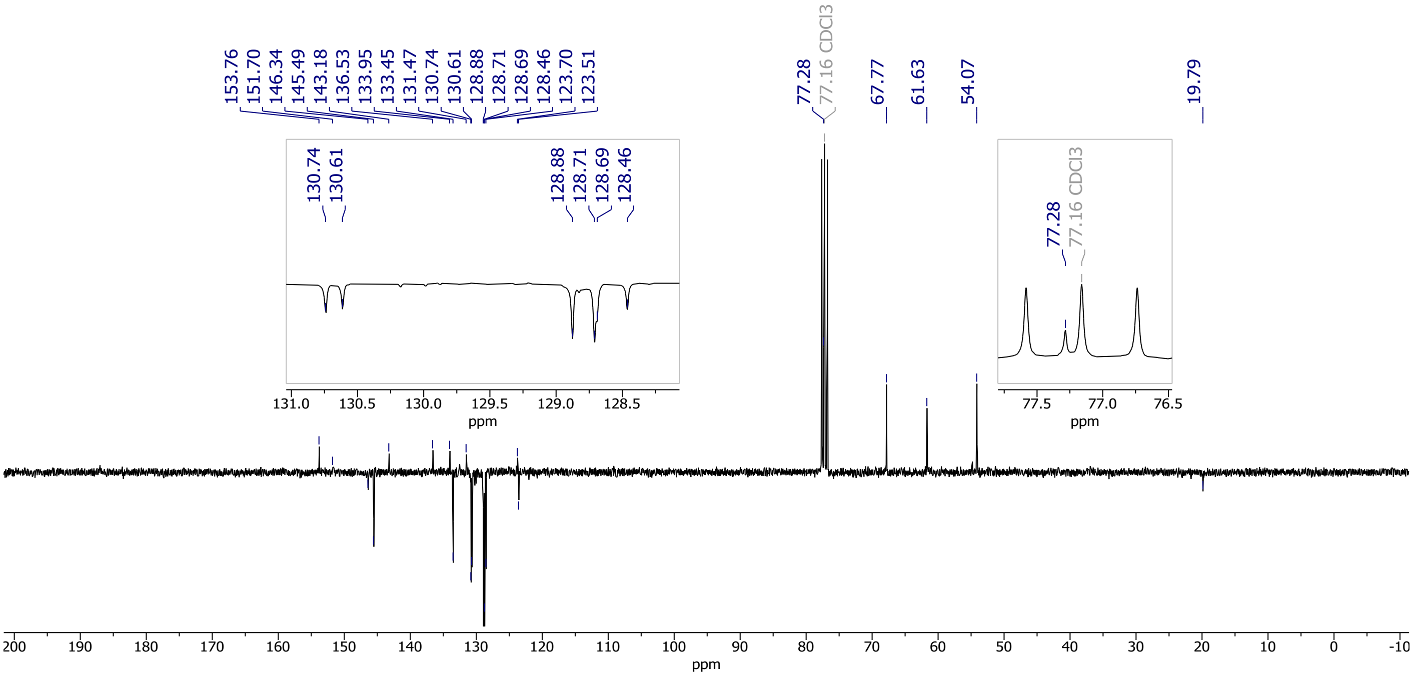

(*E*)-3-((1-(2-bromobenzyl)-1*H*-1,2,3-triazol-4-yl)methoxy)-5-(hydroxymethyl)-2-methylisonicotinaldehyde *O*-methyl oxime (7b)

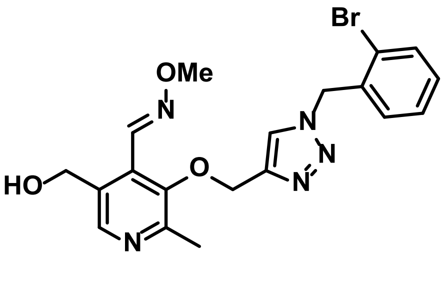

^{1}H NMR (300 MHz, CDCl$_3$)

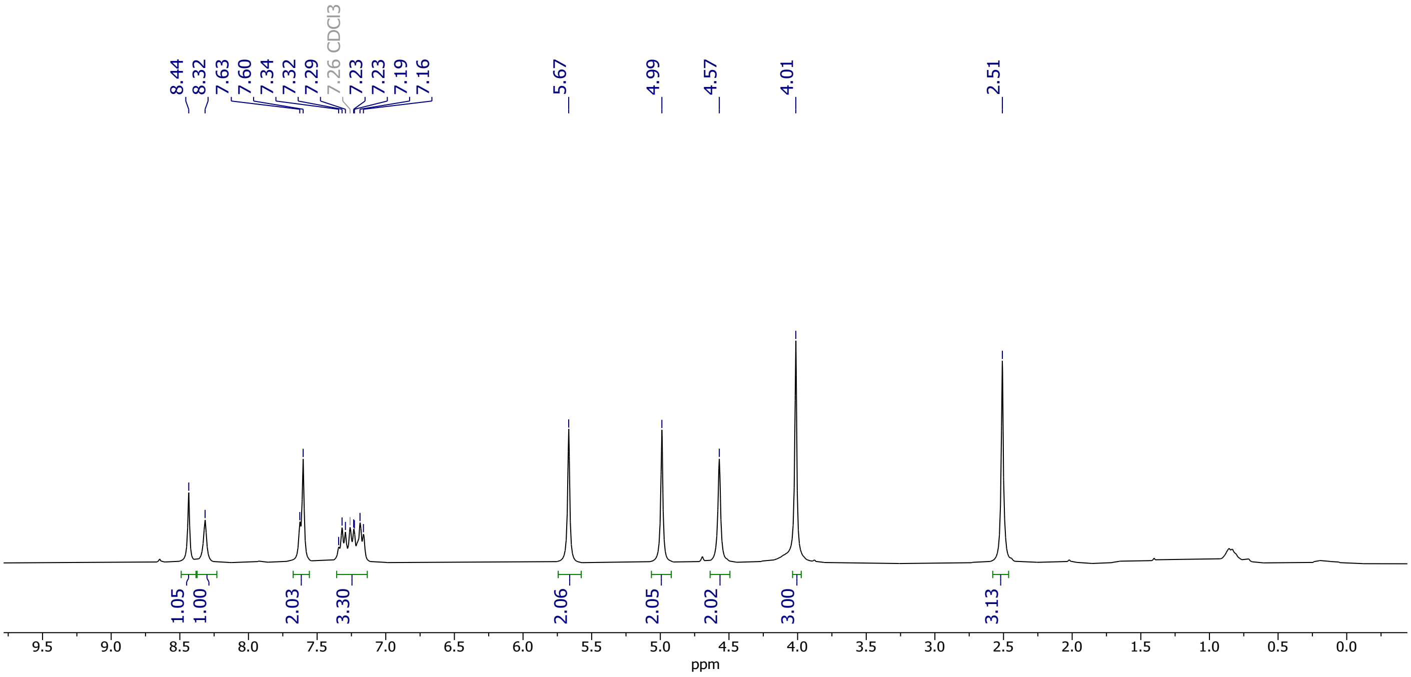

¹³C NMR (75 MHz, CDCl₃)

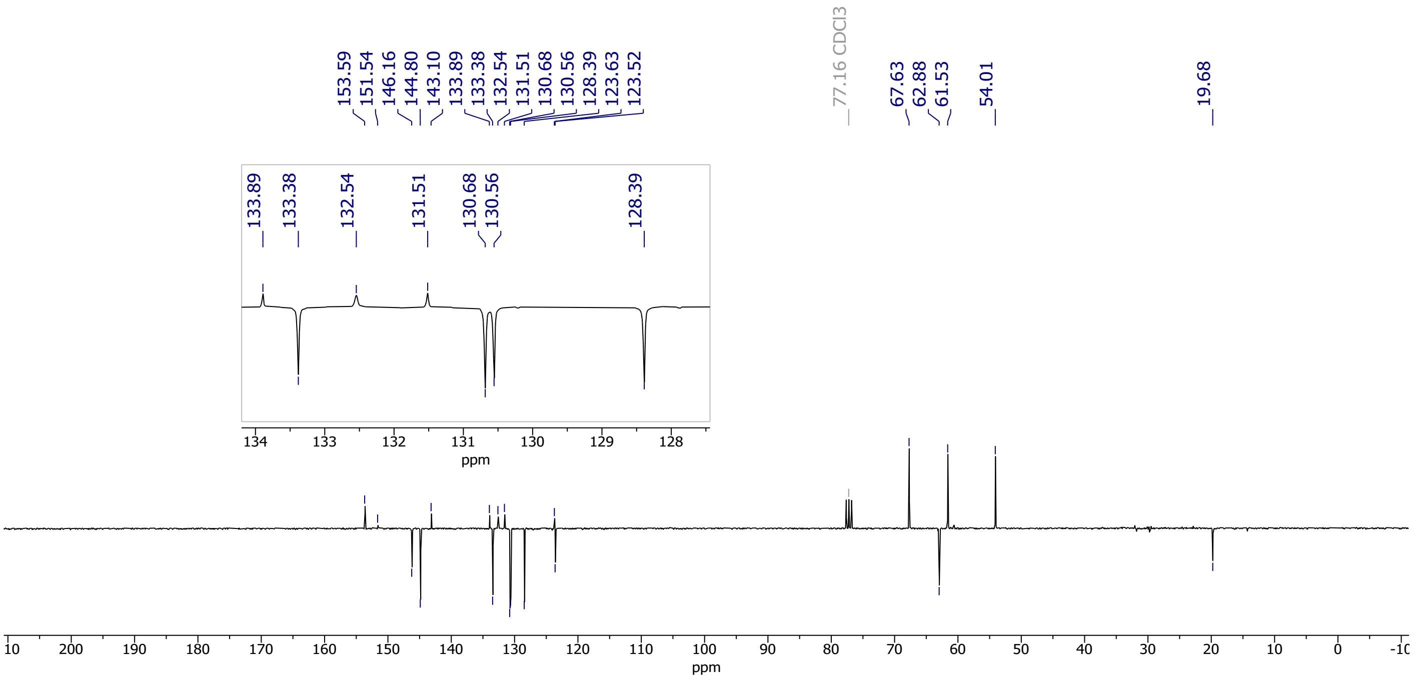

(*E*)-5-((1-(2-bromobenzyl)-1*H*-1,2,3-triazol-4-yl)methoxy)-4-((methoxyimino)methyl)-6-ethylnicotinaldehyde (7c)

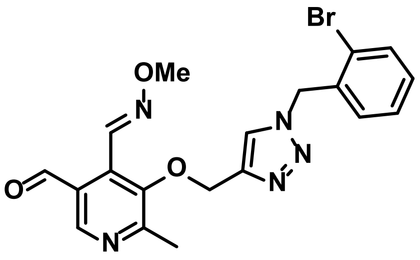

¹H NMR (300 MHz, CDCl₃)

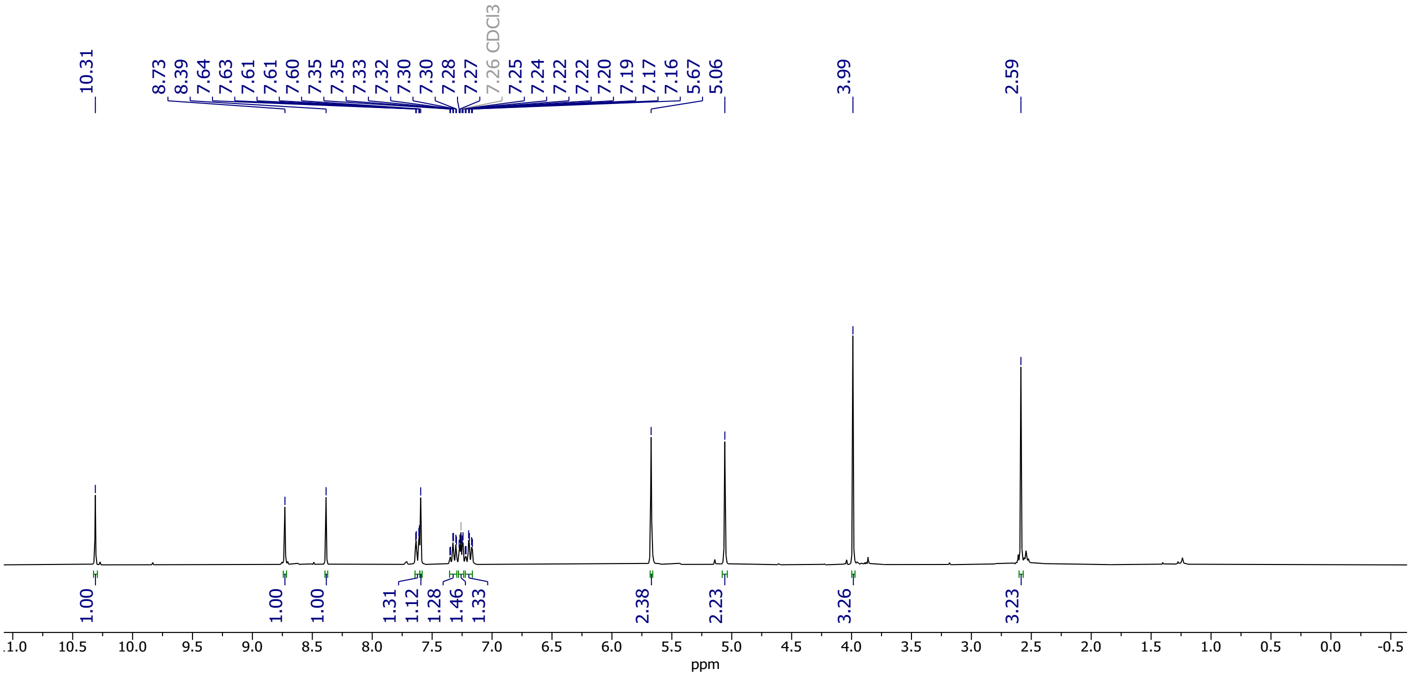

^{13}C NMR (75 MHz, $CDCl_3$)

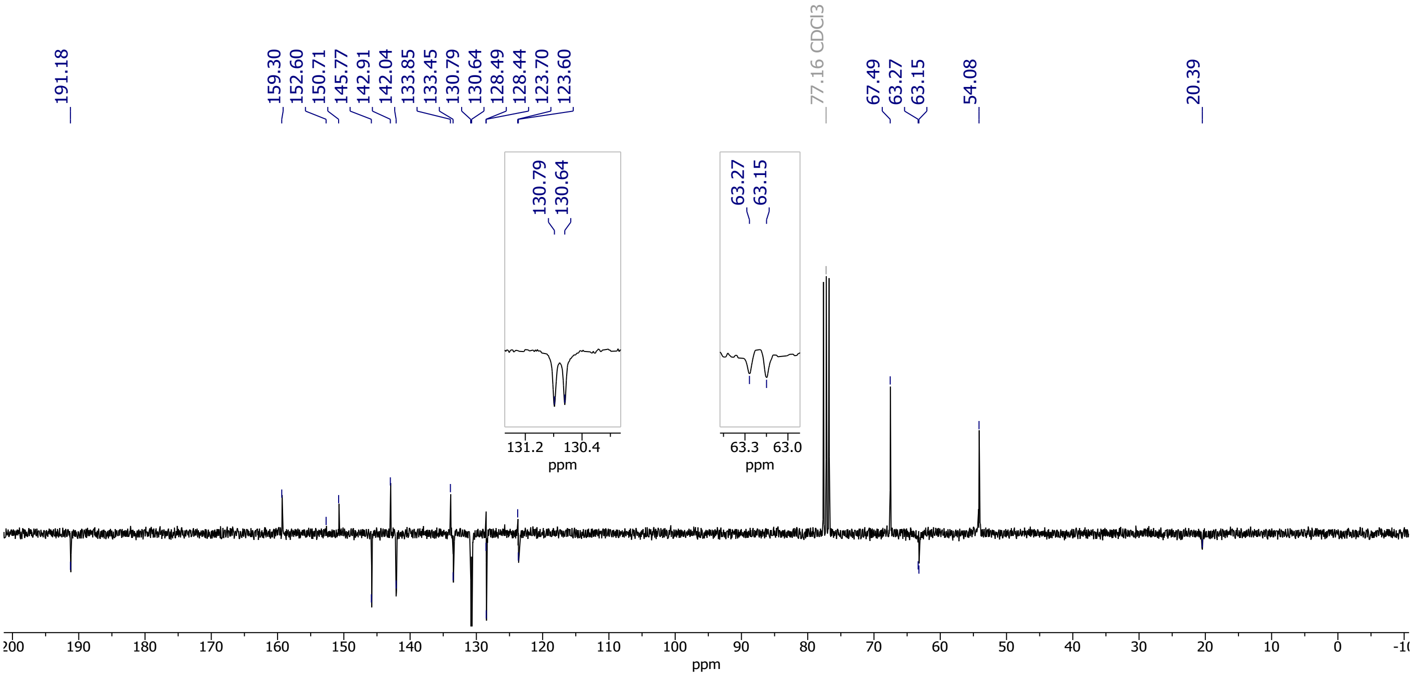

***(E)*-3-((1-(2-bromobenzyl)-1*H*-1,2,3-triazol-4-yl)methoxy)-2-methyl-5-((*Z*)-styryl)isonicotinaldehyde *O*-benzyl oxime (7d)**

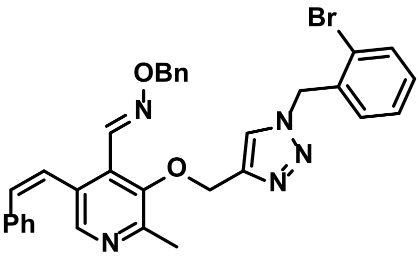

^{1}H NMR (300 MHz, $CDCl_3$)

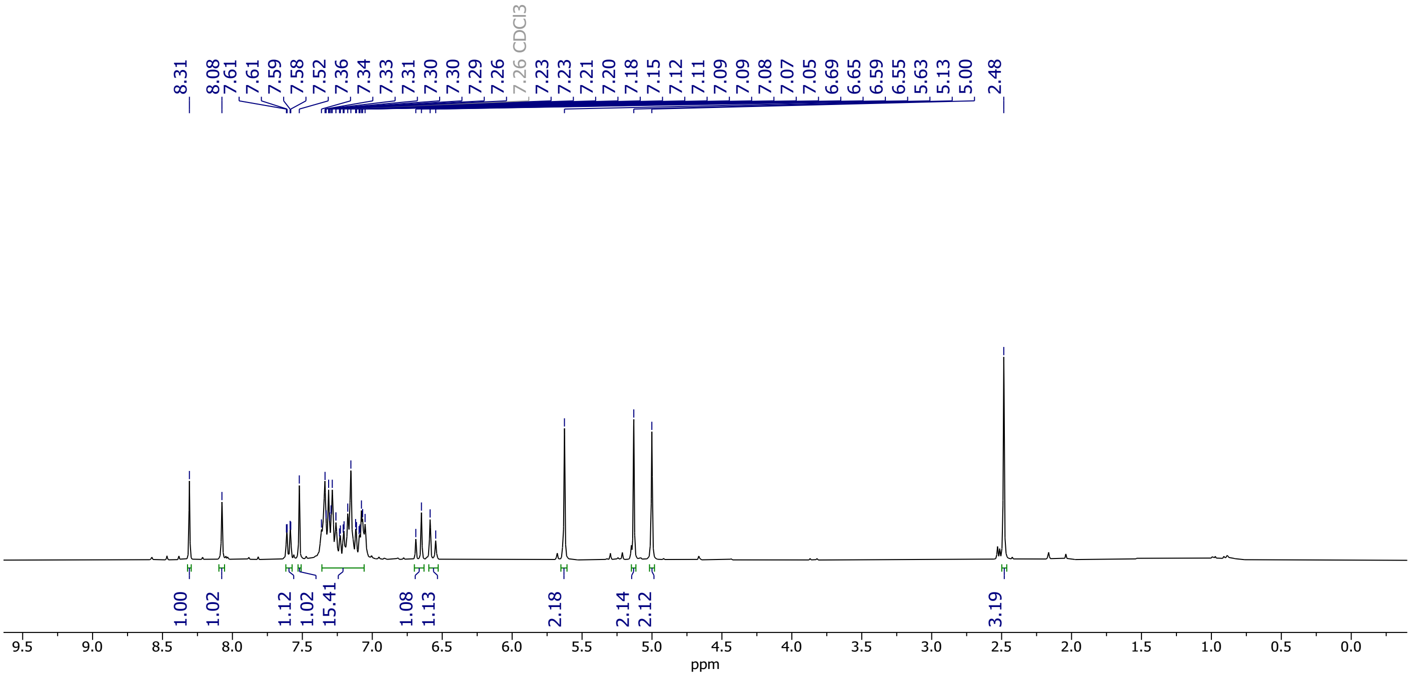

^{13}C NMR (75 MHz, CDCl$_3$)

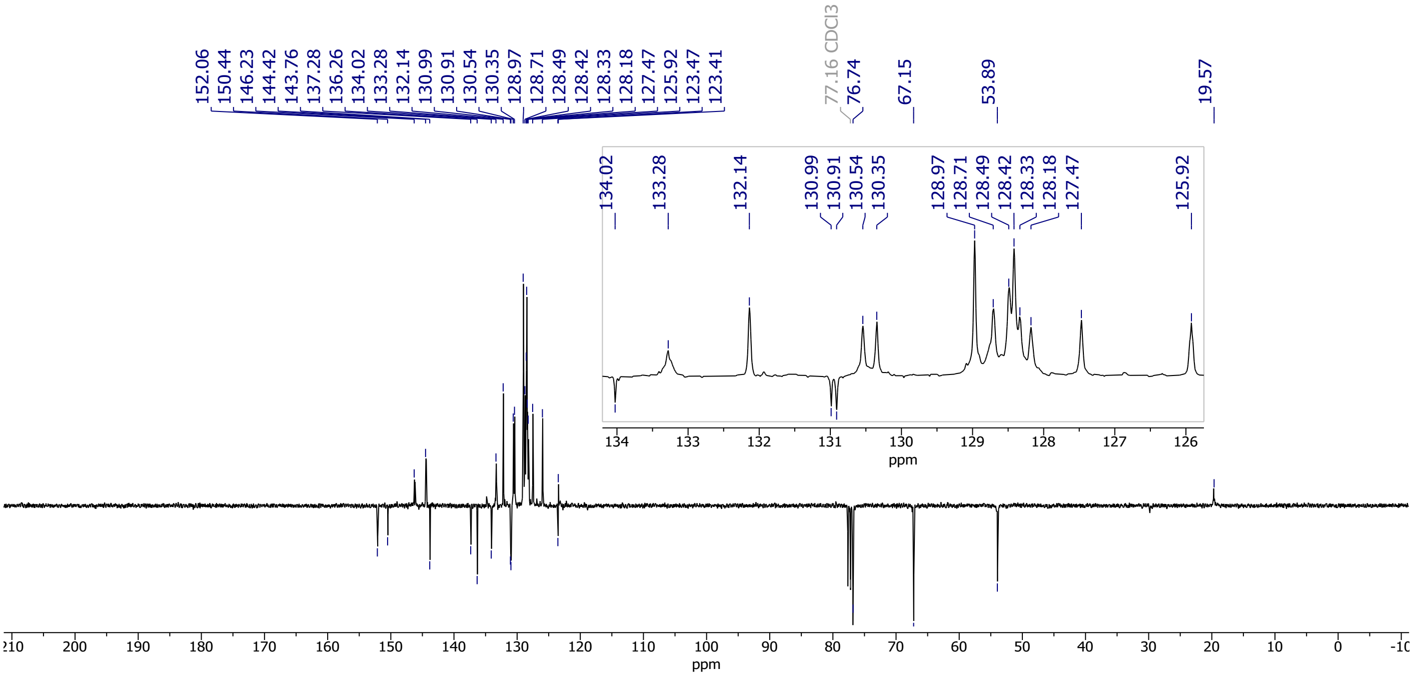

(*E*)-3-((1-cinnamyl-1*H*-1,2,3-triazol-4-yl)methoxy)-5-(hydroxymethyl)-2-methylisonicotinaldehyde *O*-benzyl oxime (7e)

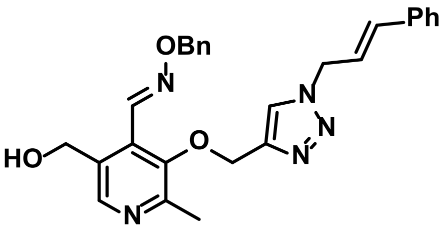

^{1}H NMR (300 MHz, CDCl$_3$)

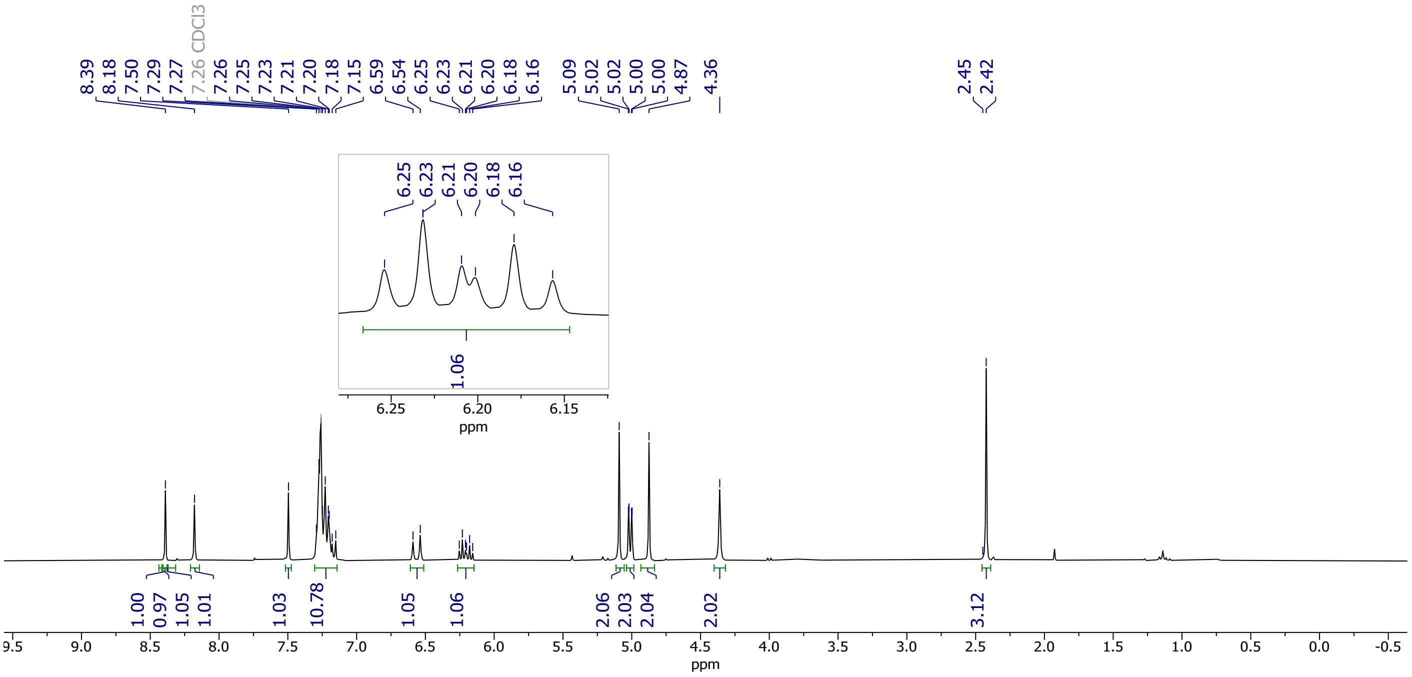

^{13}C NMR (75 MHz, CDCl$_3$)

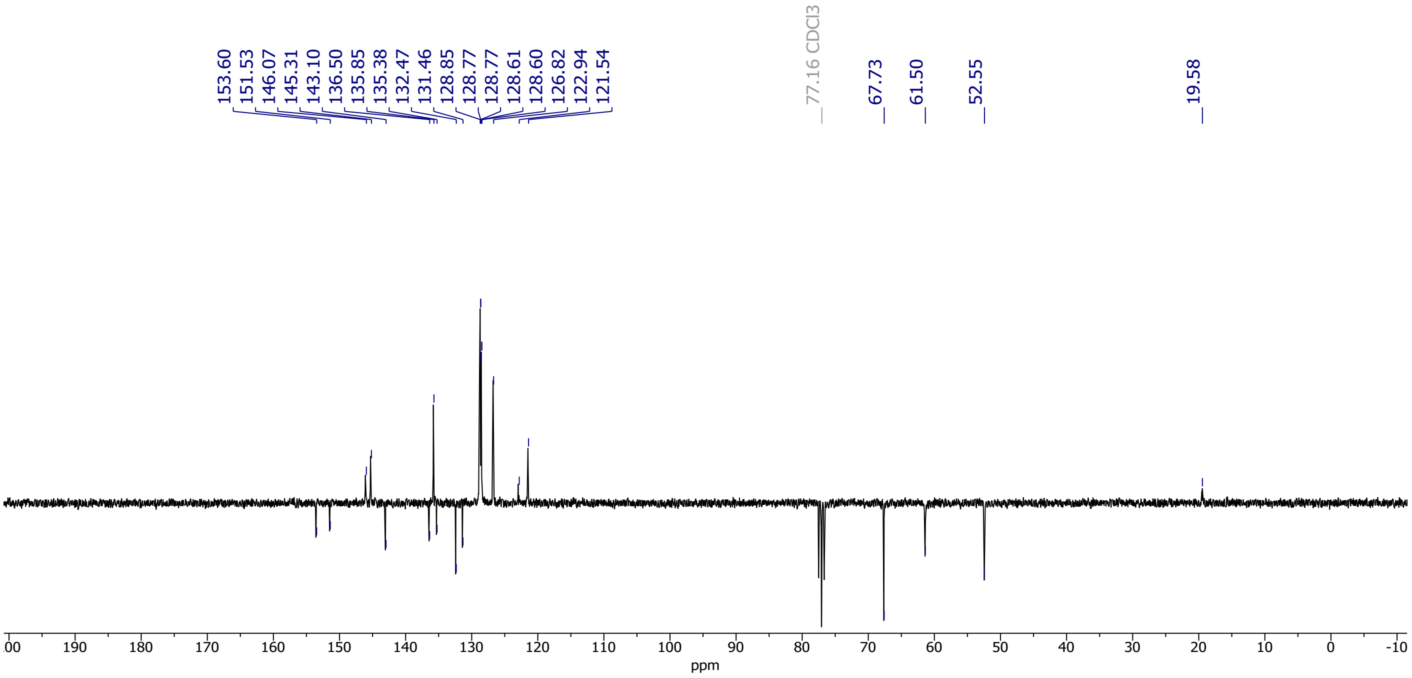

(*E*)-3-((1-cinnamyl-1*H*-1,2,3-triazol-4-yl)methoxy)-5-(hydroxymethyl)-2-methylisonicotinaldehyde *O*-methyl oxime (7f)

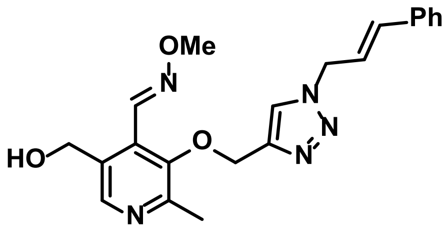

^{1}H NMR (300 MHz, CDCl$_3$)

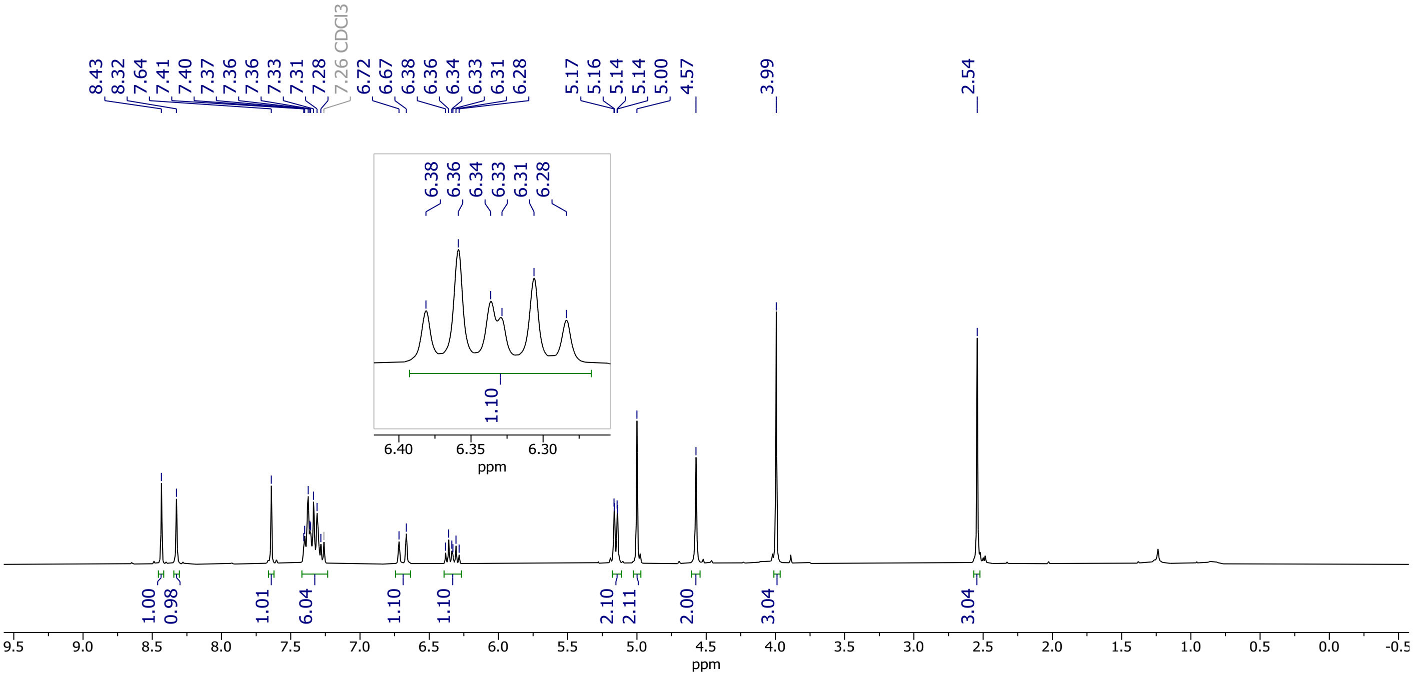

^{13}C NMR (75 MHz, CDCl₃)

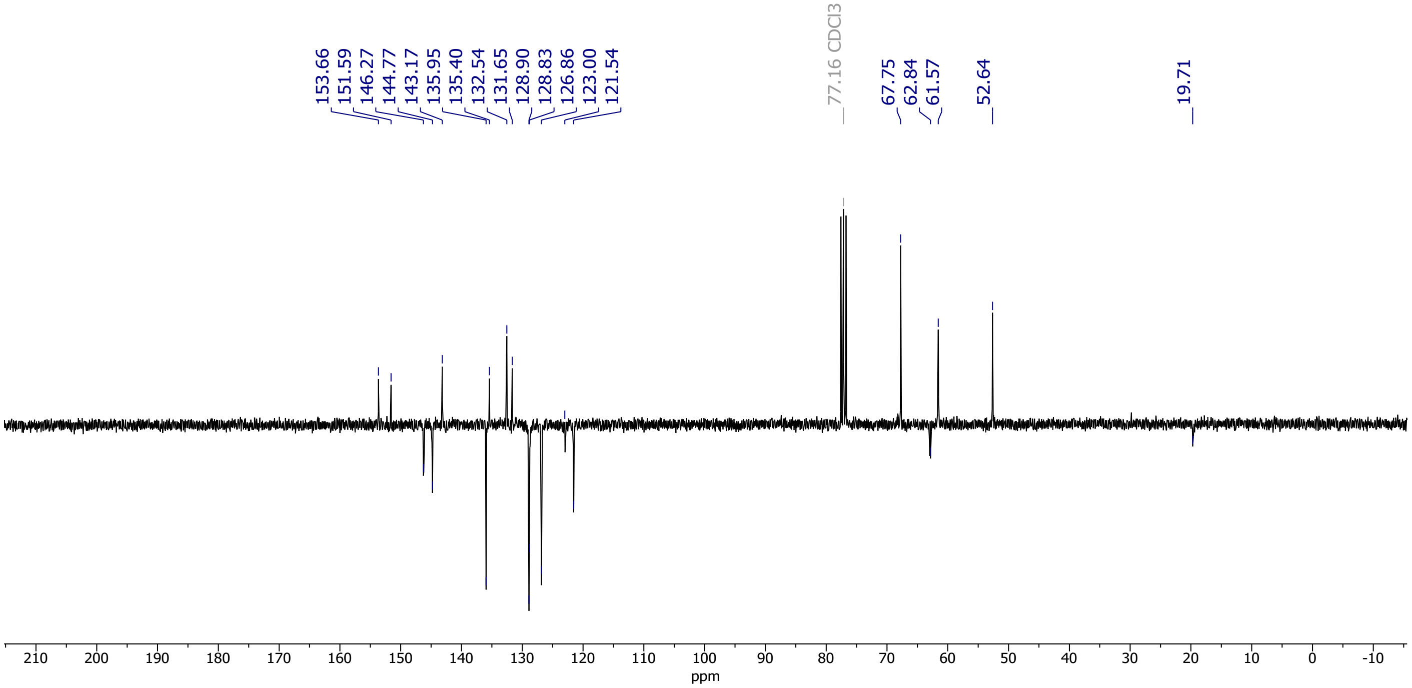

(*E*)-5-((1-cinnamyl-1*H*-1,2,3-triazol-4-yl)methoxy)-4-((methoxyimino)methyl)-6-methylnicotinaldehyde (7g)

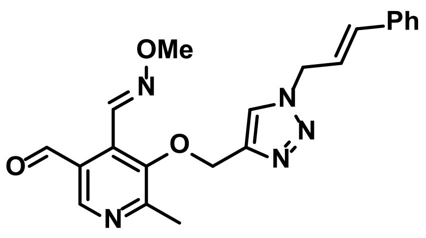

¹H NMR (300 MHz, CDCl₃)

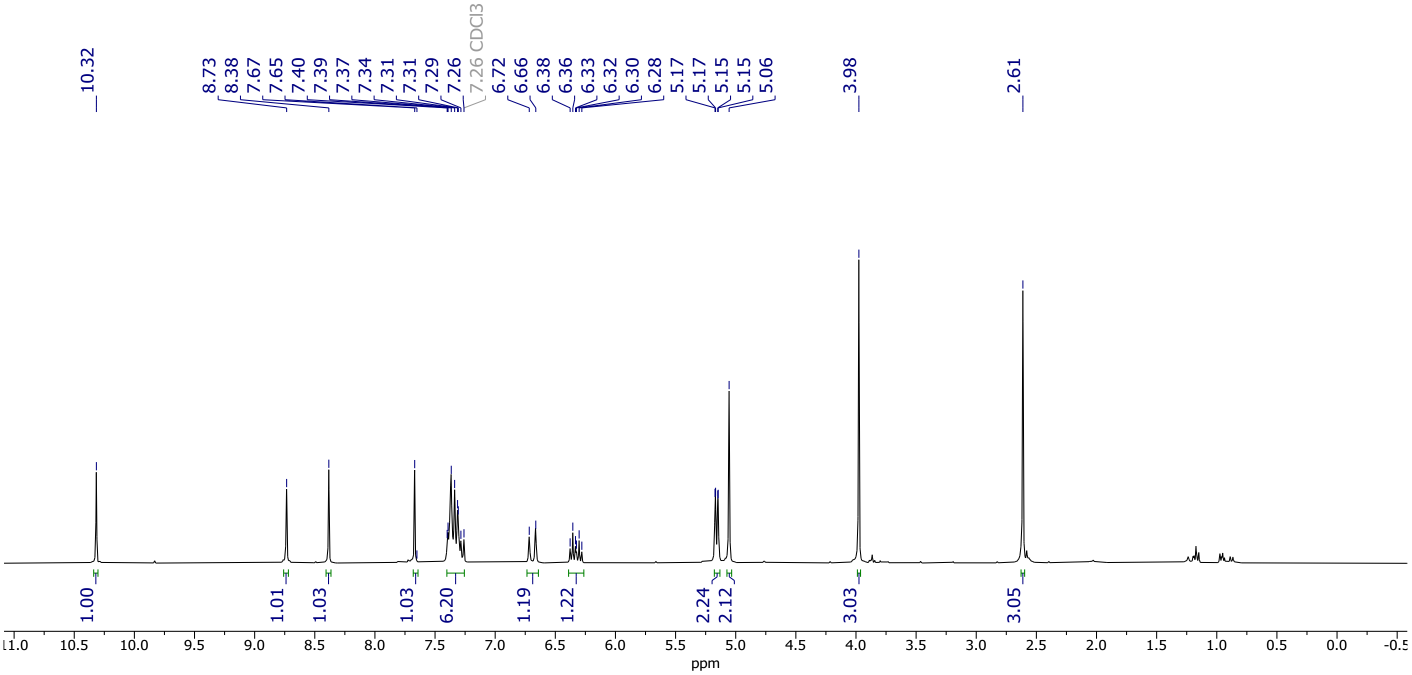

¹³C NMR (75 MHz, CDCl₃)

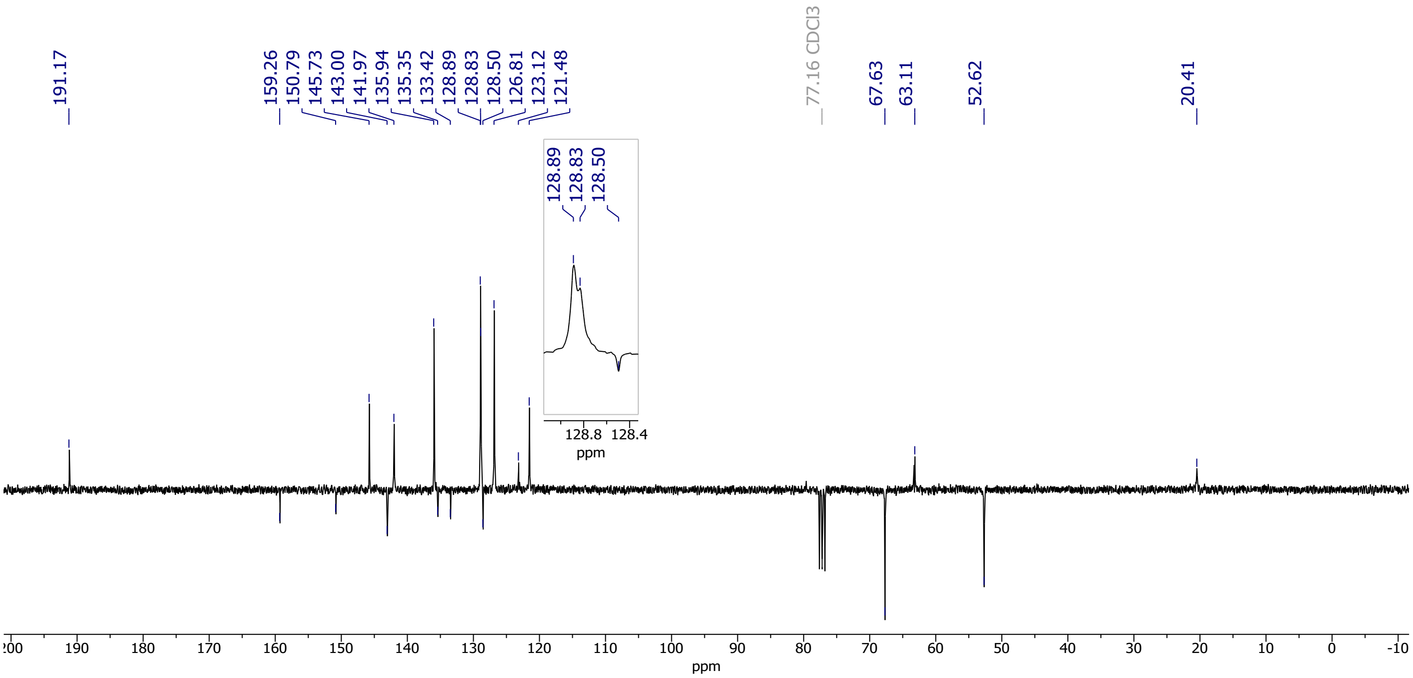

(*E*)-5-(hydroxymethyl)-2-methyl-3-(prop-2-yn-1-yloxy)isonicotinaldehyde-*O*-prop-2-yn-1-yl oxime (8a)

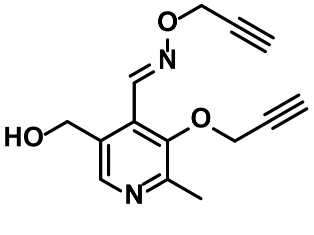

¹H NMR (400 MHz, CDCl₃)

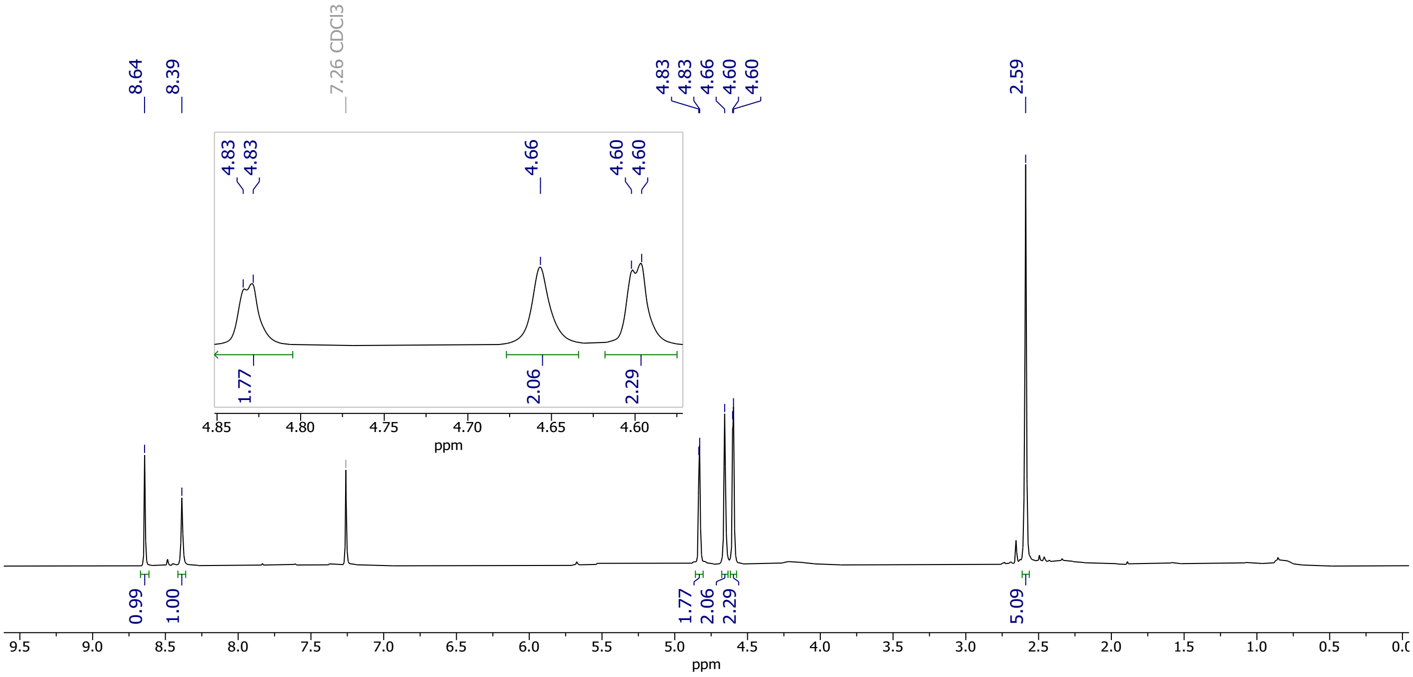

^{13}C NMR (101 MHz, $CDCl_3$)

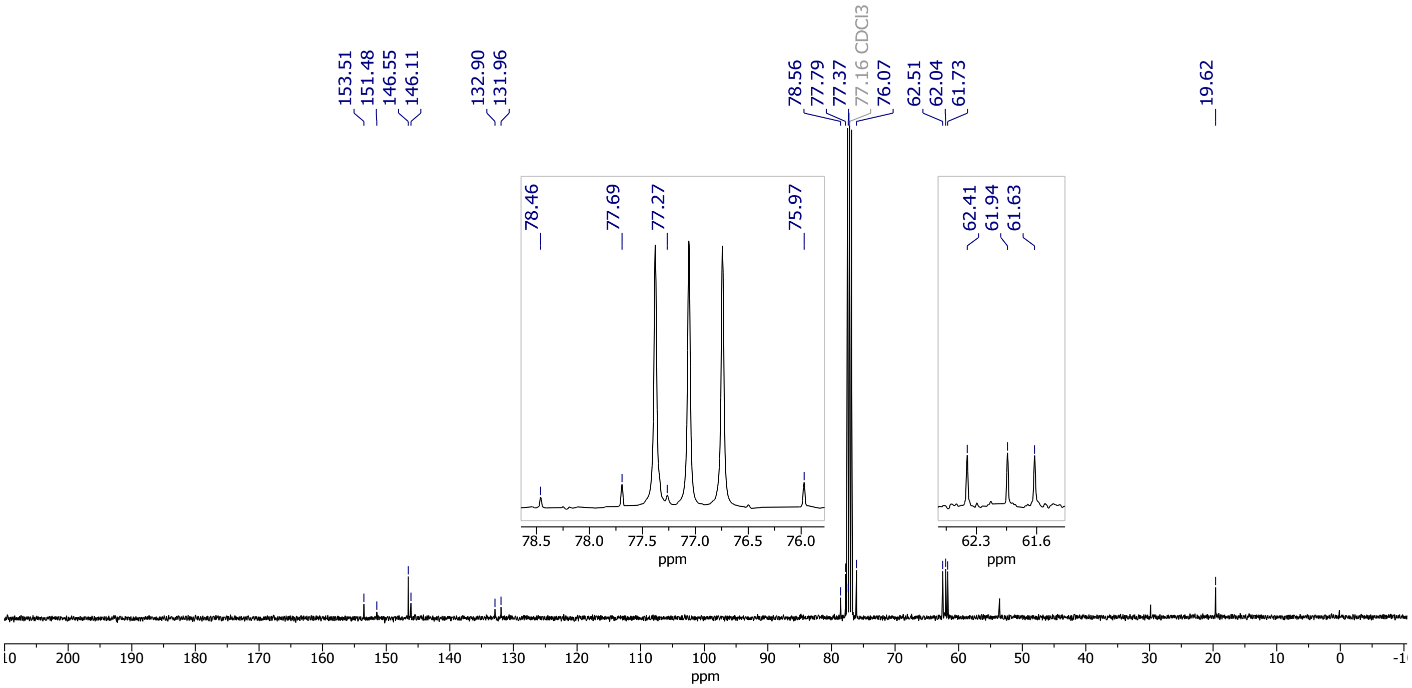

(*E*)-3-((1-(2-bromobenzyl)-1*H*-1,2,3-triazol-4-yl)methoxy)-5-(hydroxymethyl)-2-ethylisonicotionaldehyde *O*-((1-(2-bromobenzyl)-1*H*-1,2,3-triazol-4-yl)methyl) oxime (9a)

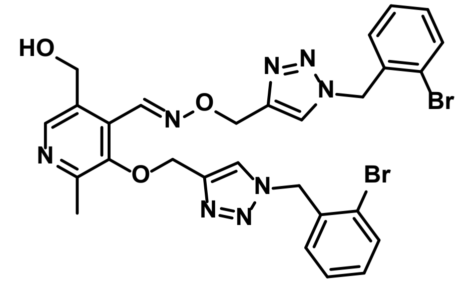

^{1}H NMR (400 MHz, CDCl₃)

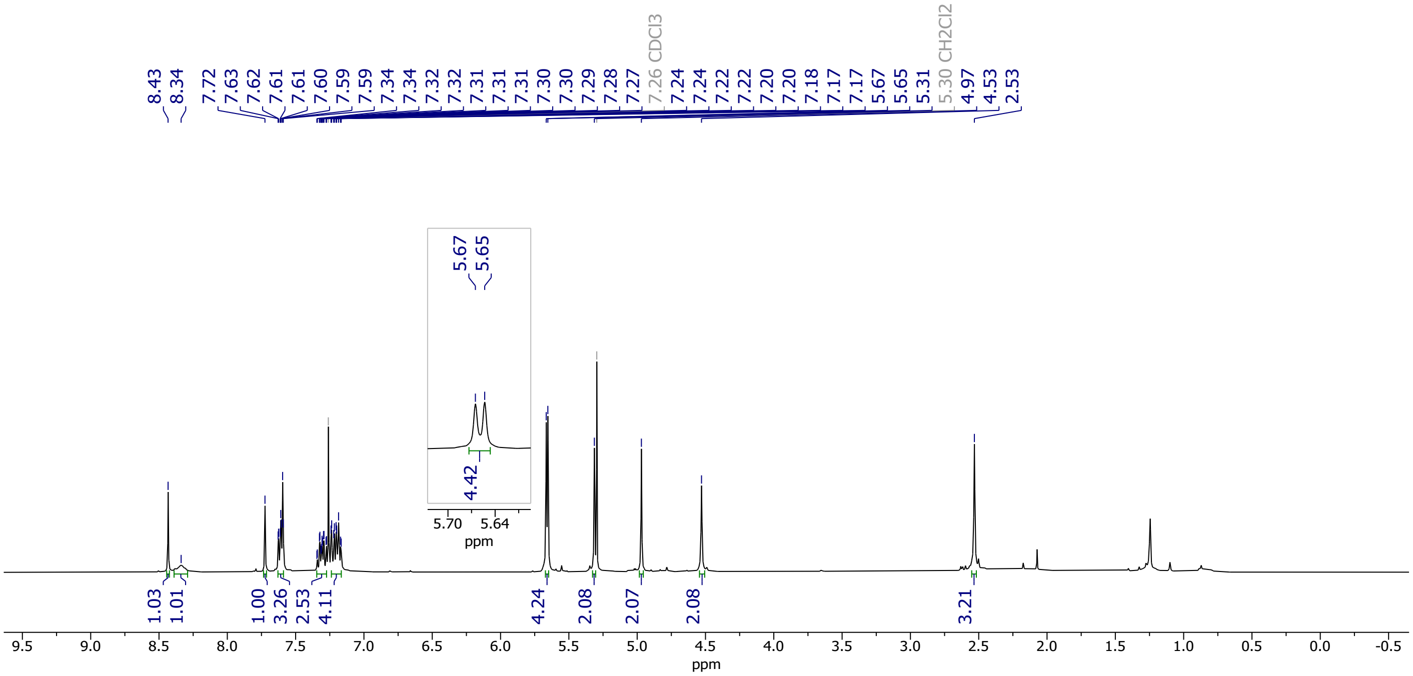

^{13}C NMR (101 MHz, CDCl₃)

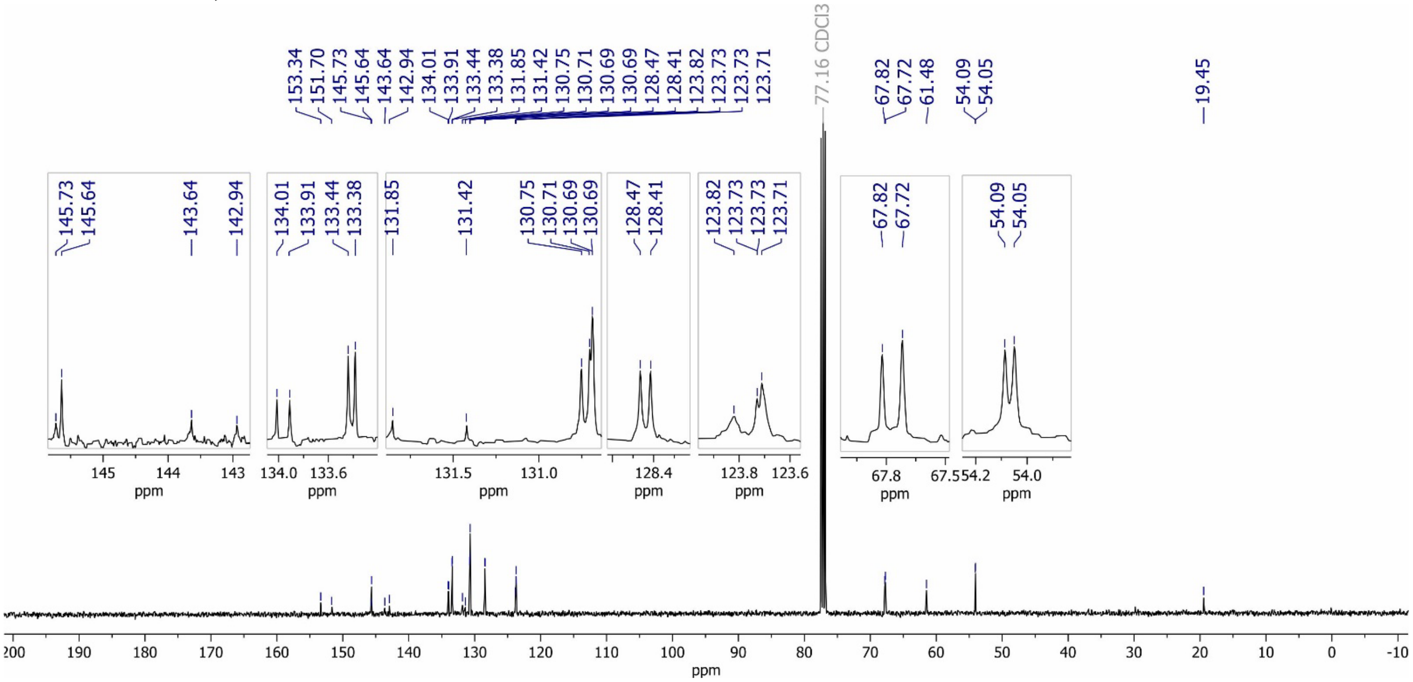

(*E*)-3-((1-cinnamyl-1*H*-1,2,3-triazol-4-yl)methoxy)-5-(hydroxymethyl)-2-ethylisonicotinaldehyde *O*-((1-cinnamyl-1*H*-1,2,3-triazol-4-yl)methyl) oxime (9b)

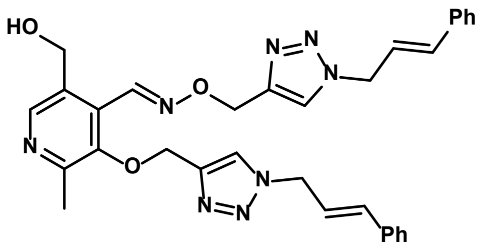

^{1}H NMR (400 MHz, CDCl3)

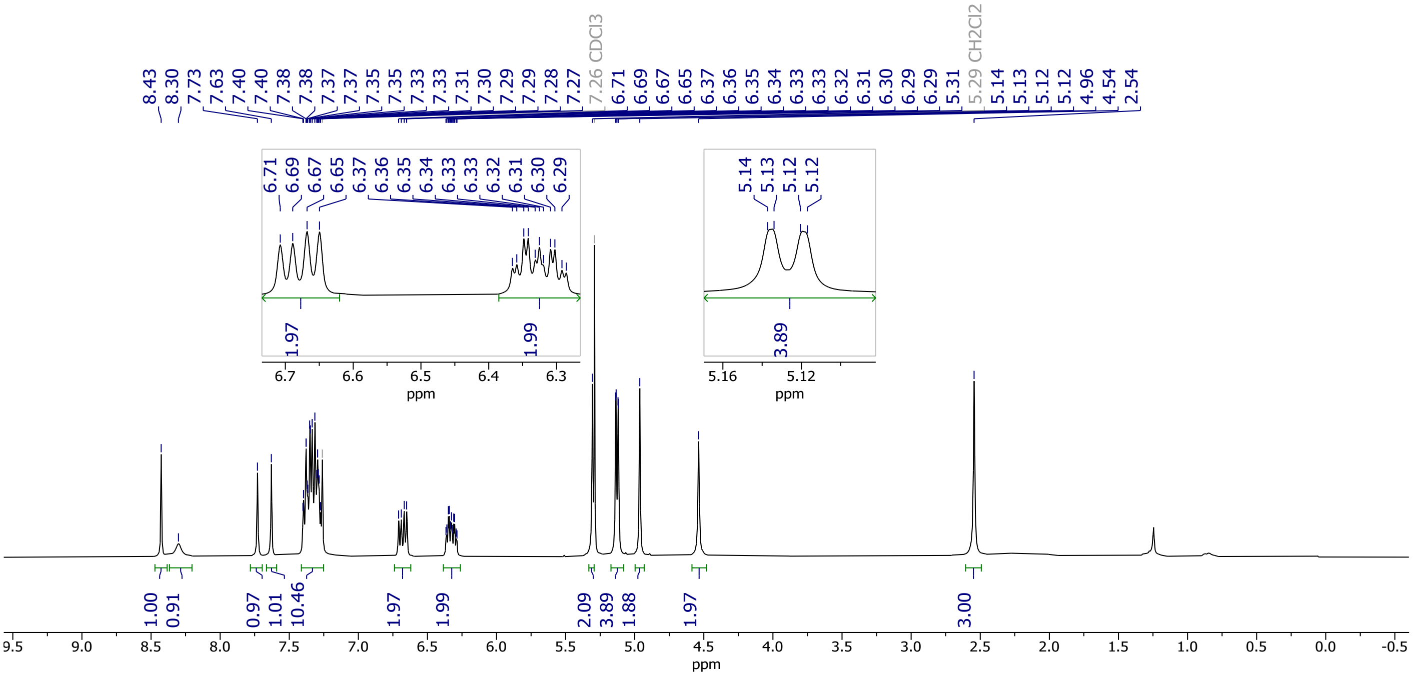

^{13}C NMR (101 MHz, CDCl3)

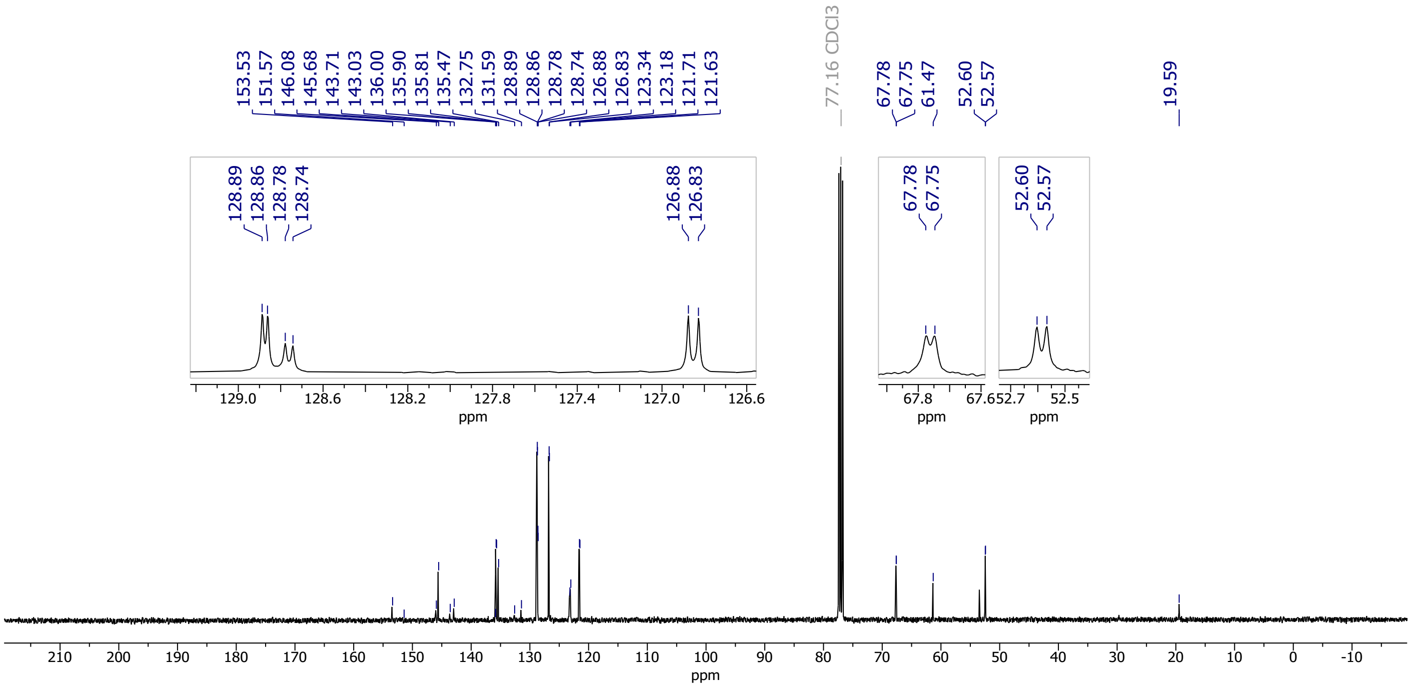

(*E*)-2-(prop-2-yn-1-yloxy)benzaldehyde *O*-benzyl oxime (21a)

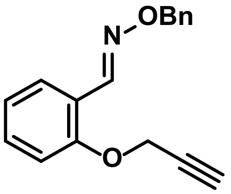

^{1}H NMR (400 MHz, CDCl$_3$)

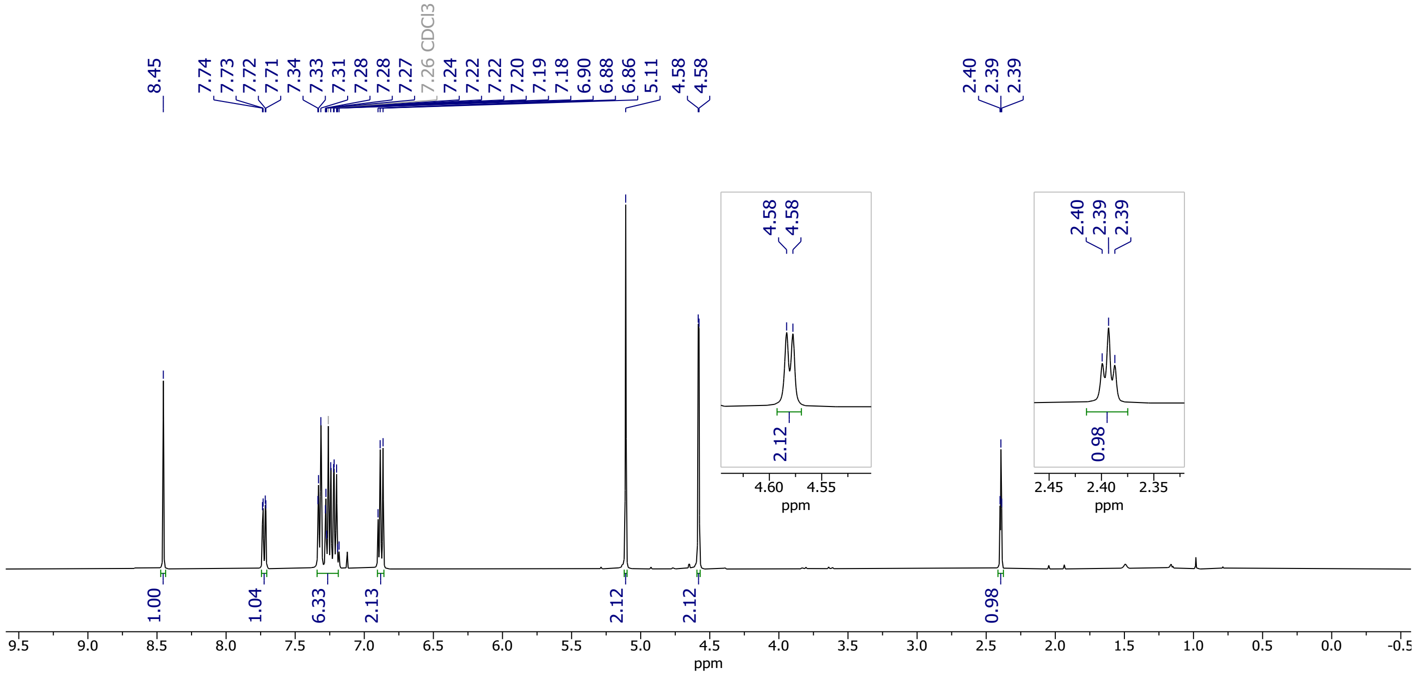

^{13}C NMR (101 MHz, CDCl$_3$)

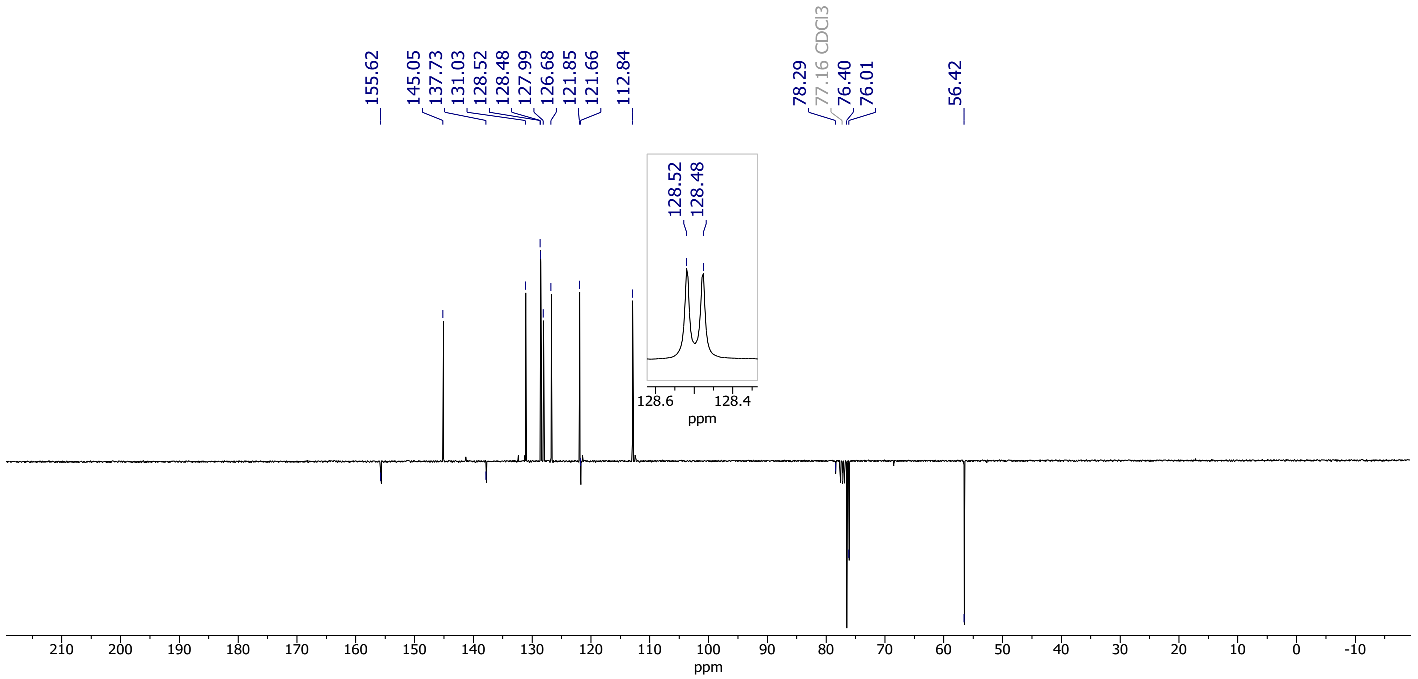

(*E*)-2-(prop-2-yn-1-yloxy)benzaldehyde *O*-methyl oxime (21b)

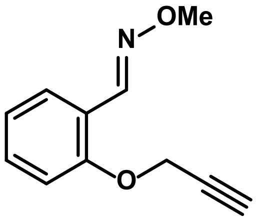

^{1}H NMR (400 MHz, CDCl[3])

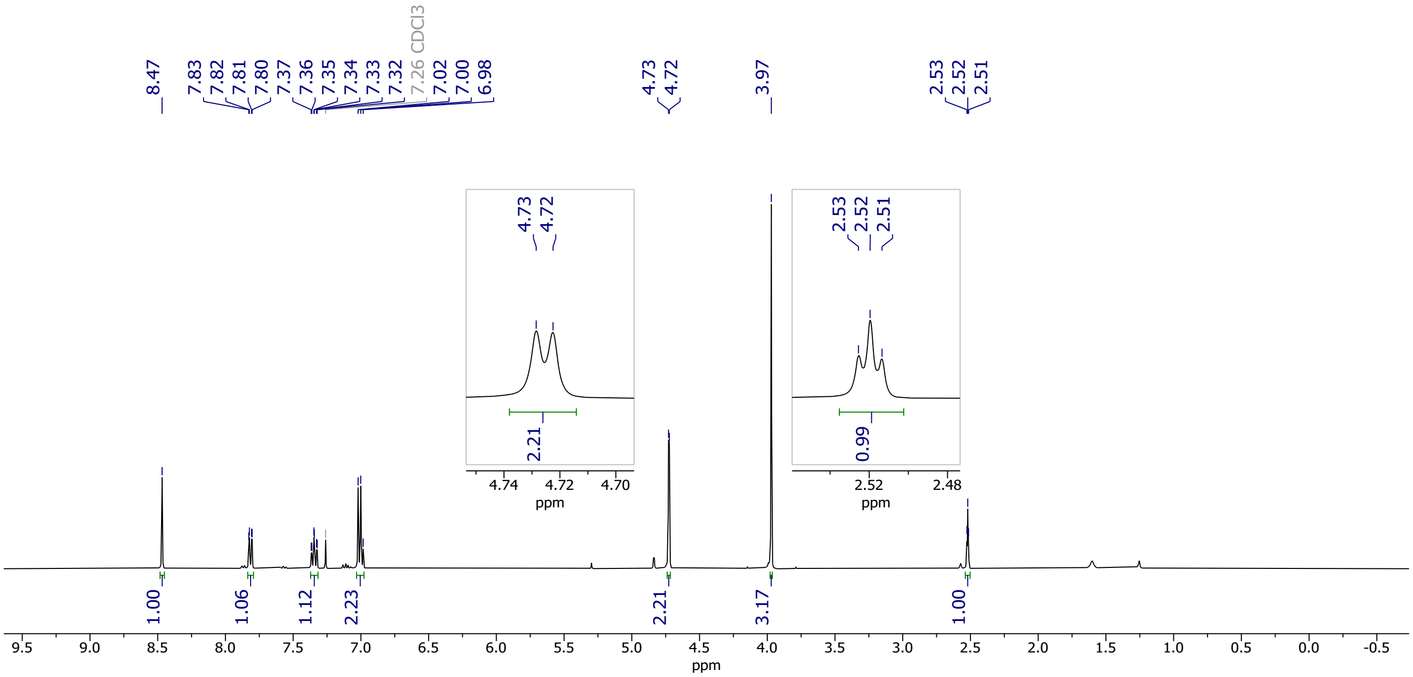

^{13}C NMR (101 MHz, CDCl[3])

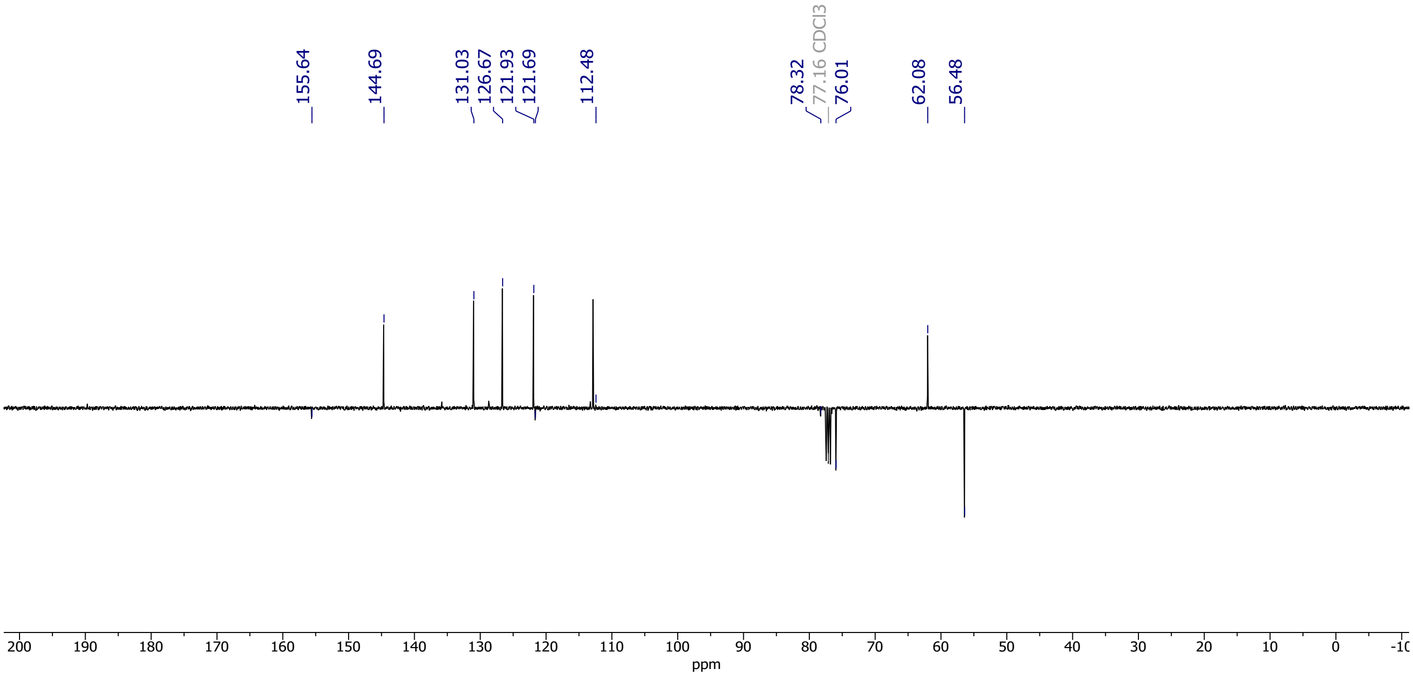

3. Antifungal activity assays

Table S1. *In vitro* antifungal activity of pyridoxal derivatives against *Candida* spp. and *C. neoformans*

Compound (μg/mL)	*C. albicans* SC5314		*C. albicans* HUSI-PUJ 256		*C. auris* HUSI-PUJ 435		*C. auris* HUSI-PUJ 537		*C. neoformans* H99		*C. neoformans* 2807	
	**MIC**	**MFC**	**MIC**	**MFC**	**MIC**	**MFC**	**MIC**	**MFC**	**MIC**	**MFC**	**MIC**	**MFC**
FLC	1	-	64	-	8	-	128	-	4	-	32	-
Pyridoxal	>300	>300	>300	>300	>300	>300	>300	>300	>300	>300	>300	>300
n-Bu$_3$SnH	<4,6	<4,6	9,3	9,3	<4,6	<4,6	<4,6	<4,6	<4,6	<4,6	<4,6	<4,6
**2a**	>300	>300	>300	>300	>300	>300	>300	>300	>300	>300	>300	>300
**2b**	>300	>300	>300	>300	>300	>300	>300	>300	>300	>300	>300	>300
**3a**	>300	>300	>300	>300	>300	>300	>300	>300	>300	>300	>300	>300
**3b**	>300	>300	>300	>300	>300	>300	>300	>300	>300	>300	>300	>300
**4a**	>300	>300	>300	>300	>300	>300	>300	>300	300	>300	>300	>300
**4b**	300	300	300	300	300	300	300	300	150	300	150	300
**4c**	>300	>300	>300	>300	>300	>300	>300	>300	>300	>300	>300	>300
**4d**	>300	>300	>300	>300	>300	>300	>300	>300	>300	>300	>300	>300
**5a**	>300	>300	>300	>300	>300	>300	>300	>300	>300	>300	>300	>300
**5b**	>300	>300	>300	>300	>300	>300	>300	>300	>300	>300	>300	>300
**5c**	>300	>300	>300	>300	>300	>300	>300	>300	>300	>300	>300	>300
**6a**	75	150	150	300	75	300	300	300	19	19	19	19
**6b**	9	19	75	150	75	75	75	150	19	38	19	38
**7a**	>300	>300	>300	>300	>300	>300	>300	>300	>300	>300	>300	>300
**7b**	>300	>300	>300	>300	>300	>300	>300	>300	>300	>300	300	300
**7c**	>300	>300	>300	>300	>300	>300	>300	>300	>300	>300	>300	>300
**7d**	>300	>300	>300	>300	>300	>300	>300	>300	>300	>300	>300	>300
**7e**	>300	>300	>300	>300	>300	>300	>300	>300	>300	>300	>300	>300
**7f**	>300	>300	>300	>300	>300	>300	>300	>300	>300	>300	>300	>300
**7g**	>300	>300	>300	>300	>300	>300	>300	>300	>300	>300	>300	>300
**8a**	>300	>300	>300	>300	>300	>300	>300	>300	>300	>300	>300	>300
**9a**	>300	>300	>300	>300	>300	>300	>300	>300	>300	>300	>300	>300
**9b**	>300	>300	>300	>300	>300	>300	>300	>300	>300	>300	>300	>300

Table S2. *In vitro* antifungal activity of salicylaldehyde derivatives against *Candida* spp. And *C. neoformans*

**Compound (μg/mL)**	***C. albicans* SC5314**		***C. albicans* HUSI-PUJ 256**		***C. auris* HUSI-PUJ 435**		***C. auris* HUSI-PUJ 537**		***C. neoformans* H99**		***C. neoformans* 2807**	
	**MIC**	**MFC**	**MIC**	**MFC**	**MIC**	**MFC**	**MIC**	**MFC**	**MIC**	**MFC**	**MIC**	**MFC**
**FLC**	1	-	64	-	8	-	128	-	4	-	32	-
**11a**	>300	>300	>300	>300	>300	>300	>300	>300	>300	>300	>300	>300
**11b**	>300	>300	>300	>300	>300	>300	>300	>300	>300	>300	>300	>300
**12a**	>300	>300	>300	>300	>300	>300	>300	>300	>300	>300	>300	>300
**12b**	38	75	75	150	300	300	150	300	150	300	150	>300
**15a**	>300	>300	>300	>300	>300	>300	>300	>300	300	>300	>300	>300
**15b**	150	150	300	300	150	300	150	300	75	150	75	150
**16a**	>300	>300	>300	>300	>300	>300	>300	>300	>300	>300	>300	>300
**19a**	150	>300	300	>300	>300	>300	>300	>300	300	>300	>300	>300
**19b**	150	300	150	>300	75	150	150	300	38	75	38	75
**21a**	>300	>300	>300	>300	150	300	150	300	150	300	150	300
**21b**	>300	>300	>300	>300	>300	>300	>300	>300	>300	>300	>300	>300

4. ADMET analysis

Figure S1. Bioavailability radars for the compounds that showed activity

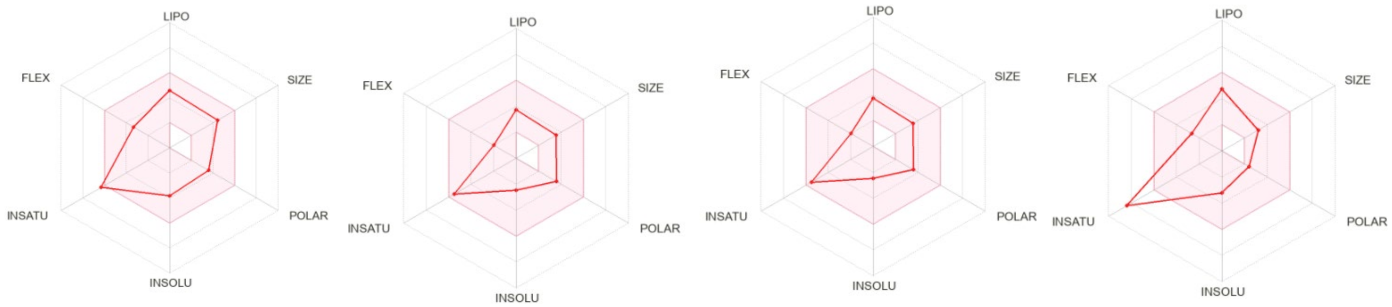

6a **6b** **12b** **19b**

Figure S2. The BOILED-EGG chart for the studied compounds

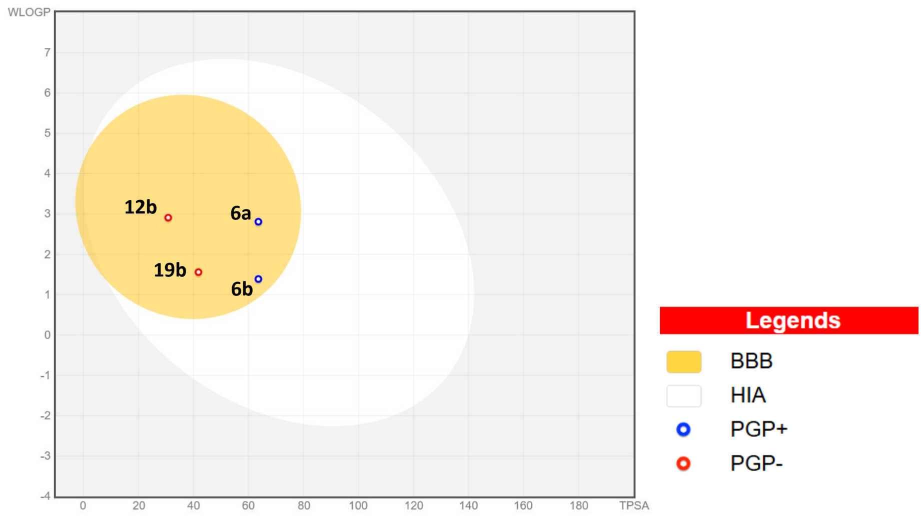

References

1. C. A. Bejarano *et al.*, "Study of the Mechanism of 7-exo-trig Cyclizations of Aryl, Vinyl, and Alkyl Radicals on Oxime Ethers," *European J Org Chem*, vol. 27, no. 7, Feb. 2024, doi: 10.1002/ejoc.202301148.
2. S. E. Booth, P. R. Jenkins, C. J. Swainb, and J. Sweeney, "Intramolecular Addition of Vinyl and Aryl Radicals to Oxime Ethers in the Synthesis of Five-, Six-and Seven-membered Ring Systems," *J. Chem. Soc., Perkin Trans. 1,* vol. 23, pp. 3499–3508, 1994, doi: org/10.1039/P19940003499.
